# Material composition and mechanical properties of the venom-injecting forcipules in centipedes

**DOI:** 10.1186/s12983-024-00543-1

**Published:** 2024-08-23

**Authors:** Simon Züger, Wencke Krings, Stanislav N. Gorb, Thies H. Büscher, Andy Sombke

**Affiliations:** 1 Department of Evolutionary Biology, Integrative Zoology, University of Vienna, Djerassiplatz 1, 1030 Vienna, Austria; 2 Department of Functional Morphology and Biomechanics, Kiel University, Am Botanischen Garten 1-9, 24118 Kiel, Germany; 3 Department of Cariology, Endodontology and Periodontology, Universität Leipzig, Liebigstrasse 12, 04103 Leipzig, Germany; 4 Center for Anatomy and Cell Biology, Cell and Developmental Biology, Medical University of Vienna, Schwarzspanierstrasse 17, 1090 Vienna, Austria

**Keywords:** Arthropoda, Chilopoda, Tarsungulum, Sclerotization, Elemental incorporation, Nanoindentation, Breaking stress

## Abstract

**Background:**

Centipedes are terrestrial and predatory arthropods that possess an evolutionary transformed pair of appendages used for venom injection—the forcipules. Many arthropods incorporate reinforcing elements into the cuticle of their piercing or biting structures to enhance hardness, elasticity or resistance to wear and structural failure. Given their frequent exposure to high mechanical stress, we hypothesise that the cuticle of the centipede forcipule might be mechanically reinforced. With a combination of imaging, analytical techniques and mechanical testing, we explore the centipede forcipule in detail to shed light on its morphology and performance. Additionally, we compare these data to characteristics of the locomotory leg to infer evolutionary processes.

**Results:**

We examined sclerotization patterns using confocal laser-scanning microscopy based on autofluorescence properties of the cuticle (forcipule and leg) and elemental composition by energy-dispersive X-ray spectroscopy in representative species from all five centipede lineages. These experiments revealed gradually increasing sclerotization towards the forcipular tarsungulum and a stronger sclerotization of joints in taxa with condensed podomeres. Depending on the species, calcium, zinc or chlorine are present with a higher concentration towards the distal tarsungulum. Interestingly, these characteristics are more or less mirrored in the locomotory leg’s pretarsal claw in Epimorpha. To understand how incorporated elements affect mechanical properties, we tested resistance to structural failure, hardness (*H*) and Young’s modulus (*E*) in two representative species, one with high zinc and one with high calcium content. Both species, however, exhibit similar properties and no differences in mechanical stress the forcipule can withstand.

**Conclusions:**

Our study reveals similarities in the material composition and properties of the forcipules in centipedes. The forcipules transformed from an elongated leg-like appearance into rigid piercing structures. Our data supports their serial homology to the locomotory leg and that the forcipule’s tarsungulum is a fusion of tarsus and pretarsal claw. Calcium or zinc incorporation leads to comparable mechanical properties like in piercing structures of chelicerates and insects, but the elemental incorporation does not increase *H* and *E* in centipedes, suggesting that centipedes followed their own pathways in the evolutionary transformation of piercing tools.

**Supplementary Information:**

The online version contains supplementary material available at 10.1186/s12983-024-00543-1.

## Background

Arthropods use specific structures as tools to interact directly with their environment [1–3]. Such tools are e.g., mandibles, leg claws, ovipositors, stings or chelicerae that often exhibit higher concentrations of transition and alkaline earth metals with colocalised halogens at their cuticular cutting edges or piercing tips [4, 5]. Venom-injecting structures or stings of arthropods are mostly derived from appendages or their parts (with some exceptions such as the scorpion stinger). These structures share a common characteristic, as they must withstand considerable stress when puncturing cutaneous tissue of their prey. After puncture, venom is injected whose chemical compounds induce effects from minor anaesthesia to the death of the prey [6]. The incorporation of metals in the cuticle of stings is thought to ensure an efficient performance of these structures as injection needles [7] that allows animals to sustain prolonged use of these structures for environmental interactions [8]. Generally, metal enriched arthropod cuticles may contain alkaline earth metals as calcium (Ca) or magnesium (Mg) and/ or transition metals as copper (Cu), iron (Fe), manganese (Mn) or zinc (Zn). For instance, leaf-cutter ants incorporate Zn into their mandibular teeth cuticle during early adulthood, resulting in a three-fold increase in hardness directly related to the Zn content [1, 9]. In pseudoscorpions aluminium (Al) is incorporated into the tip of their venom-injecting pedipalps, while Zn is present around the venom gland opening [4]. Other chelicerates feature Fe, Cu, Mn, and Zn at the tips of their chelicerae, while Mn is found in the scorpion stinger [7, 10]. The cuticle of cicada ovipositors is not just enriched by a few inorganic elements, but a wide array of element incorporation is present (Cu, Fe, Mn, Zn, zirconium (Zr), Al, Ca, Mg, K, Na, Si, P and S) [11]. Metal enriched cuticles are, however, observed across all habitats and feeding behaviours in arthropods, with no particular tendencies towards herbivory or carnivory [2, 12], although a relationship with diet is suggested [13]. Another important aspect of reinforcing the arthropod cuticle involves sclerotization or tanning, a process that modifies mechanical properties, such as stiffness, hardness, breaking stress, and other characteristics by altering the molecular structure of the chitin-protein matrix of the exocuticle through chemical reactions [14, 15]. Transition and alkaline earth metals can bind to this matrix and increase thereby the cross-linking density [16–18]. The incorporation of elements was previously found to correlate with higher values of the mechanical properties Young’s modulus (*E*) and hardness (*H*), which relate to higher resistances to wear and structural failure in these areas [2, 3, 14, 19, 20], as metal ions increase the degree of cross-linking of chitin fibres [16, 21]. By analysing the cuticular autofluorescence properties using CLSM, reliable information on material composition and interpretation on the degree of sclerotization can be obtained [22–26]. Lower excitation wavelengths (405 and 458 nm) result in autofluorescence in weakly sclerotized and protein-rich cuticles. Excitations at 488, 514 or 561 nm can visualise moderately sclerotized cuticles, while excitation at 633 nm visualizes strongly sclerotized cuticles.

Centipedes (Chilopoda) possess a remarkable apomorphy in the modification of their first trunk appendages into a paired venom-delivering and piercing weapon—the forcipule [27–29]. This unique evolutionary transformation involved both internal and external transformations. Externally, the size and shape of the podomeres changed drastically in comparison to the locomotory legs [30, 31]. Internally, a massive venom gland evolved featuring a common duct [27, 32]. The serial homology of the forcipular podomeres with those of the locomotory legs is still debated [33]. Typically, the centipede locomotory leg consists of six podomeres: coxa, trochanter, prefemur, femur, tibia, and tarsus (the latter can be further subdivided), and a pretarsal claw [29, 33]. In the forcipule, the coxae are fused with the trunk sternite, forming a prominent coxosternite that partially overlaps with the head ventrally [33]. The telopodite is composed of only four podomeres: trochanteroprefemur, femur, tibia, and tarsungulum. Near its tip, the tarsungulum houses the venom gland opening [28].

Centipedes are exclusively terrestrial predators living in or on soil habitats, seeking shelter under stones, bark, or in leaf litter [30, 34]. They exhibit a wide food spectrum, including earthworms, spiders, and various insects, depending on the animal’s body size and life cycle stage [35]. Prey choice is influenced by the habitat structure and the prey-to-body-size ratio [36]. Larger centipedes may even consume small vertebrates [30, 35, 36]. Some species, such as *Scolopendra gigantea* Linnaeus, 1758, have been observed actively preying on bats in caves, while *Strigamia maritima* (Leach, 1817) in coastal regions feeds on barnacles and periwinkles [37, 38]. Centipedes have evolved two distinct strategies for prey capture, actively foraging when in need of food or switching to a sit-and-wait strategy when satiated [39]. Prey capture is realised in using the forcipules and the upper third of the anterior trunk. Scolopendromorphs have been observed using locomotory legs to grasp and retain large prey, and in the case of *Scutigera coleoptrata* (Linnaeus, 1758), the long tarsi with multiple annuli are used like a lasso to capture and hold prey [30, 39, 40]. Once the prey is captured, the forcipules pierce the prey's cuticle or skin and inject venom to immobilize and kill it. Centipede venom is one of the oldest extant venom systems among terrestrial animals and contains neurotoxic peptides similar to those found in scorpions and spiders [41, 42]. However, the majority of centipede toxins remain uncharacterised, and their mechanism of action is not well understood [42]. Similar to spiders, it is suggested that centipedes might use venom optimisation, modulating the venom content and amount depending on the difficulty of overpowering different types of prey [43].

While there is some information on the cuticle composition, presence of sclerotization patterns and incorporation of reinforcing elements in piercing and venom-delivering structures within Chelicerata [4, 10, 44] and aculeate Hymenoptera [45], no such information is available for Chilopoda. Our study aims to investigate the morphological characteristics and material composition of centipede forcipules by examining seven species, representing all five extant higher taxa. Prior research by Haug et al. [28] provided an initial overview on the forcipular morphology in representative species focusing on joint positions and general size differences, but their analysis of cuticular autofluorescence was limited to 488 nm excitation. In our study, we build upon this research and broaden the investigation by incorporating and combining several techniques. Specifically, we employed scanning electron microscopy (SEM) to achieve higher resolution images in combination with evaluations of the elemental composition by energy-dispersive X-ray spectroscopy (EDX) to gain insights into the material composition of the cuticle. We used excitations at six different wavelengths by confocal laser scanning microscopy (CLSM) to gain a more comprehensive understanding of the degree of sclerotization in the forcipular cuticle based on its autofluorescence properties. Furthermore, we utilised microCT analyses to study the thickness and radiodensity of the forcipular cuticle. Additionally, we compared the morphology of the centipede forcipule with that of an ordinary locomotory leg to explore whether evolutionary transformations can be traced [29, 46]. As we observed potential diverging evolutionary pathways in forcipular reinforcements, we have chosen two species with different cuticle-enriching elements for further analyses. By examining *Lithobius forficatus* (Linnaeus, 1758) and *Cryptops hortensis* (Donovan, 1810) with breaking stress and nanoindentation experiments, our aim was to compare mechanical properties, such as hardness (*H*) and elasticity modulus (*E)* and the structures’ resistance to breakage. This combination of imaging, analytical techniques and mechanical testing allowed us to explore the forcipular morphology in greater detail and shed light on its functional significance, to uncover deeper insights into the evolutionary processes shaping the performance of this unique venom-delivering structure in arthropods.

## Material and methods

### Examined species

Species used in this study cover all five extant centipede taxa. They were taken from ethanol collections, sampled in the field, or purchased (see Table 1). At least one specimen per species was investigated (see detailed information in respective method sections). The sex was not determined in this study.

**Table 1:** Examined species and used methods

Species	Taxon	Location/collection	Methods
*Scutigera coleoptrata* (Linnaeus, 1758)	Scutigeromorpha	coll. A. Sombke, Bisamberg, near Vienna, Austria, August 2022	SEM, EDX, CLSM, µCT
*Lithobius forficatus* (Linnaeus, 1758)	Lithobiomorpha	coll. A. Sombke, Augarten, Vienna, Austria, August 2022	SEM, EDX, CLSM, µCT
*Craterostigmus tasmanianus* Pocock, 1902	Craterostigmomorpha	coll. R. Mesibov, Goderich Road, NW Tasmania, October 1991, Queen Victoria Museum 23: 5327	SEM, EDX, CLSM, µCT
*Scolopendra morsitans* Linnaeus, 1758	Scolopendromorpha	pet shop	µCT
*Scolopendra oraniensis* Lucas, 1846	Scolopendromorpha	coll. A. Sombke, Ibiza, Spain, September 2018	SEM, EDX, CLSM
*Cryptops hortensis* (Donovan, 1810)	Scolopendromorpha	coll. A. Sombke & S. Züger, Augarten, Vienna, Austria, August 2022, Botanical Garden, Kiel, Germany, July 2023	SEM, EDX, CLSM, µCT
*Strigamia maritima* (Leach, 1817)	Geophilomorpha	coll. J. Rosenberg, southeast of Luc-sur-Mer, France, July 1978	SEM, EDX, CLSM, µCT
*Haplophilus subterraneus* (Shaw, 1789)	Geophilomorpha	coll. A. Sombke, Greifswald, Germany, August 2020	SEM, EDX, CLSM

### Scanning *electron* microscopy (SEM) and energy dispersive X-ray spectroscopy (EDX)

**Fig. 1:**
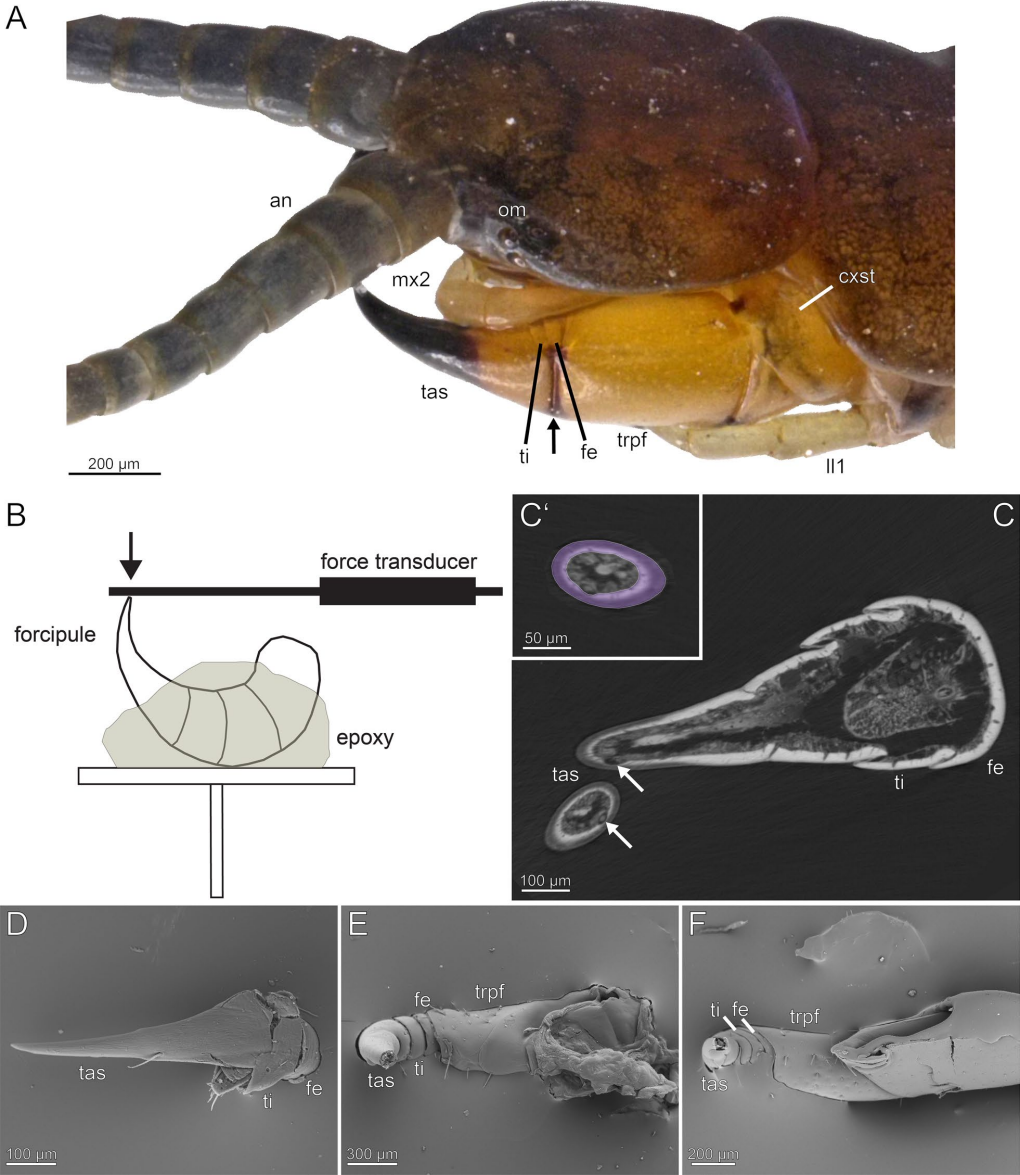
*Scolopendra oraniensis* and experimental setup and results from breaking force/stress experiments on *Scutigera coleoptrata, Lithobius forficatus* and *Cryptops hortensis.* **A** Head and anterior trunk of *Scolopendra oraniensis* in dorsolateral view. The forcipule is composed of coxosternite, trochanteroprefemur, femur, tibia and tarsungulum (proximal to distal). Arrow points to shared joints of trochanteroprefemur, femur, tibia and tarsungulum. **B** Experimental setup. The forcipule is embedded in epoxy and the tarsungulum is in a straight, free-standing position. The force transducer is lowered onto the tip of the tarsungulum. **C** Longitudinal-section through the forcipule of *L. forficatus* (microCT). The arrows point to the venom channel. **C’** Cross-section of the tarsungulum of *L. forficatus* at the point of breakage. Purple area indicates the measured cuticle area used for stress calculations. **D** Exemplary forcipule of *S. coleoptrata* after the breaking force/stress measurement. The tarsungulum is not broken, but plastically deformed. **E** Exemplary forcipule of *L. forficatus* after measurement. **F** Exemplary forcipule of *C. hortensis* after measurement. **an** antenna. **cxst** coxosternite. **fe** femur. **ll1** locomotory leg 1. **mx2** maxilla 2. **om** ommatidia. **tas** tarsungulum. **ti** tibia. **trpfe** trochanteroprefemur

The external morphology of the forcipules (Fig. 1A) and locomotory legs was examined using scanning electron microscopy (SEM). In addition, energy dispersive X-ray spectroscopy (EDX) was employed to analyse the presence or absence of specific elements in the cuticle. For each species, at least one specimen was used to examine the forcipule and the locomotory leg 10. In the case of *S. coleoptrata*, an additional specimen kept for several months in captivity was used. For *S. oraniensis*, two specimens were used—one kept in captivity for over a year and one caught in the wild without being fed between capture and dissection. The purpose was to compare whether differences in elemental distribution and concentration occurred depending on whether the specimens were kept in ethanol for a long period of time or were freshly fixed after being kept in captivity or caught in nature. The freshly prepared locomotory leg 10 and forcipule were not fixed, but air-dried for three days and then mounted on adhesive carbon-conducted tabs. The specimens stored in ethanol were dehydrated in a graded series of ethanol concentrations (70%, 80%, 90%, 96%) for 20 min in each concentration. They were then kept on a shaker in 100% ethanol overnight. The samples were cleaned in an ultrasonic bath for two rounds of 20 s each and air-dried for one hour before being mounted on carbon-conducted tabs.

EDX mapping analyses were performed using a JEOL IT 300 LV scanning electron microscope (JEOL Ltd., Tokyo, Japan) with an Octane Plus detector (EDAX Ametek) and the APEX software at the Core Facility Cell Imaging and Ultrastructure Research (University of Vienna). The measurement time was 12 min using the mapping setting. The analysis detected the presence of several elements, and their proportions (atomic % and mass %) were measured. These elements included C (carbon), N (nitrogen), O (oxygen), Na (sodium), Mg (magnesium), Al (aluminium), Si (silicon), P (phosphorus), S (sulphur), Cl (chlorine), K (potassium), Ca (calcium), Mn (manganese), Fe (iron), Co (cobalt), Ni (nickel), Cu (copper), Zn (zinc), and, in sputter-coated samples, Au (gold). The given percentages are semi-quantitative, as the detector was calibrated using Cu. This leads to a high confidence on the results for heavier elements but a lower confidence for lighter elements. The elemental proportions of the locomotory leg and the forcipule were tested for each species. C, N, and O were excluded from interpretation, since they are present in all organic samples. Mn, Co, and Ni appeared with negligible percentages and often stayed below the minimum detection level (MDL). Cu and Fe were also excluded from interpretation due to either staying below the MDL or lacking clear localisation, likely due to random noise. All measured atomic % are shown in detail in Additional file 1.

Following EDX measurements, all samples were sputter-coated with gold and examined using detectors for secondary and backscatter electrons. To further analyse whether the detected elements were present throughout the cuticle, the tip of the forcipule was cut off in *Craterostigmus tasmanianus*, *Cryptops hortensis*, *Strigamia maritima* and *Haplophilus subterraneus*.

To gain insights into potential gradients with regard to metals, one forcipule of *Lithobius forficatus* and *Cryptops hortensis* was examined by point-measurements [47]. For this, the dried forcipules were attached to glass slides with double-sided adhesive carbon tape. The forcipules were then surrounded by a metallic ring and immersed in epoxy resin (Reckli Epoxy WST, RECKLI GmbH, Herne, Germany). After polymerisation at room temperature lasting 3 days, the glass slide and carbon tape were removed and the samples were polished with sandpapers of different roughness until the longitudinal sections of the forcipules were on display. The samples were then smoothened with aluminium oxide polishing powder suspension of 0.3 µm grain size (PRESI GmbH, Hagen, Germany) using a polishing machine (Minitech 233/333, PRESI GmbH, Hagen, Germany), cleaned in an ultrasonic bath for 3 min, mounted on SEM sample holders and coated with platinum (5 nm layer). The samples were then tested with the SEM Zeiss LEO 1525 (Carl Zeiss MicroImaging GmbH, Wetzlar, Germany) equipped with an Octane Silicon Drift Detector (SDD) (micro analysis system TEAM; EDAX Inc.). For both samples, the same settings were used (working distance 15 mm, lens aperture 60 µm, measurement time for each measurement point 30 s, resolution 137.6 eV). The following elements were detected and analysed: Al, C, Ca, Cl, Cu, F, Fe, K, Mg, Mn, N, Na, O, P, Pt, S, Si, Zn. However, we only considered measurements with peaks of the respective elements that were higher than the noise. Some elements were not discussed, as they are either a part of chitin and the associated proteins (C, H, N, O) or could be artefacts from the polishing paste (Al, O). Because the single peak of P (phosphorus) overlaps with one of the peaks of Pt (platinum), the software is not able to discriminate between these two elements, and therefore, P and Pt are discussed together (P + Pt).

### Confocal laser scanning microscopy (CLSM)

CLSM (confocal laser scanning microscopy) was utilised to visualise the cuticular autofluorescence of the forcipules and locomotory legs. The intensity of autofluorescence was used to infer varying degrees of sclerotization. Lesser sclerotized cuticle emits shorter wavelengths (e.g., 405 nm), while stronger sclerotized cuticle emits longer wavelengths (> 555 nm) [22, 23]. Additionally, resilin, when excited, emits autofluorescence below 405 nm [23].

For each fixed specimen, a locomotory leg 10 and a forcipule were rehydrated, immersed in decreasing concentrations of ethanol (60%, 50%, 40%, 30%) for 20 min each and then subjected to increasing concentrations of glycerine (30%, 40%, 50%, 60%, 70%, 80%, 90%, 100%) 2 times for 20 min. Subsequently, the samples were mounted in 100% glycerine on a concave glass slide with a cover slip and analysed using a Leica TCS SP5 II confocal laser scanning microscope (Leica Microsystems GmbH, Wetzlar, Germany). The initial settings were adjusted based on the methodology described by Michels and Gorb [23], and laser powers, gains, and offsets were fine-tuned according to the cuticle thickness. Six laser lines with different wavelengths (405 nm, 458 nm, 488 nm, 514 nm, 561 nm, and 633 nm) were used. A line average of 2 and frame average of 4 were set, with a resolution of either 512 × 512 pixels or 1024 × 1024 pixels.

Additionally, a cross-section of the tarsungulum of *L. forficatus* was visualised using a Zeiss LSM 700 confocal laser scanning microscope (Carl Zeiss Microscopy GmbH, Jena, Germany) with four different laser lines (405, 488, 555 and 639 nm).

The resulting image stacks, were analysed using Fiji [48] and further processed using Photopea (Ivan Kutskir, Prague, Czech Republic). Each wavelength was assigned a specific colour following the guidelines provided by Michels et al. [49]: 405 nm (blue), 458 nm (magenta), 488 nm (cyan), 514 nm (green), 561 nm (yellow), and 633 nm (red). The degree of sclerotization was assigned using the terms ‘weak’ (autofluorescence at low wavelengths), ‘moderate’ (autofluorescence at medium wavelengths), and ‘strong’ (autofluorescence at high wavelengths). Maximum-intensity projections were used for creating superimposed colour images.

### MicroCT analysis

For microCT analyses, specimens were prepared and examined according to Sombke et al. [50]. Scans were performed using a Xradia MicroXCT-200 (Carl Zeiss Microscopy GmbH, Jena, Germany) at specific settings (see Table 2). Tomography projections were reconstructed using XMReconstructor (Carl Zeiss Microscopy GmbH, Jena, Germany). All scans were performed using Binning 2 to reduce noise, and subsequently processed using Binning 1 to retain full resolution and avoid information loss. An additional scan of *Cryptops hortensis* was conducted using a Skyscan 1172 X-Ray microtomograph (Micro Photonics Inc., Allentown, USA). The tomography projections were reconstructed using Nrecon 1.0.7.4 (Bruker Corporation, Billerica, USA) software and further processed in Amira 6.4 (Thermo Fisher Scientific, Massachusetts, USA).

**Table 2:** Parameters of microCT scans

	Objective	Source settings	Exposure time	Pixel size
*Scutigera coleoptrata*	20x	40 kV, 200 µA	25 s	1.26 µm
*Lithobius forficatus*	10x	30 kV, 200 µA	3 s	1.98 µm
*Craterostigmus tasmanianus*	10x	40 kV, 200 µA	3 s	2.12 µm
*Scolopendra morsitans*	10x	40 kV, 200 µA	2 s	2.11 µm
*Cryptops hortensis*	–	59 kV, 167 µA	2 s	0.87 µm
*Strigamia maritima*	20x	40 kV, 200 µA	3 s	1.00 µm

Image stacks were used to measure cuticle thickness and radiodensity (greyscale values) of the forcipules in Fiji [48]. Radiodensity describes the property of a tissue to inhibit the passage of electromagnetic radiation.

### Nanoindentation and breaking stress analyses

The force required for breaking of a single tarsungulum was measured with a Biopac MP100 and a Biopac TCI-102 system (BIOPAC Systems, Inc., Goleta, USA). Freshly dissected forcipules were mounted on aluminium SEM stubs using epoxy resin (Reckli Epoxy WST, RECKLI GmbH, Herne, Germany) embedding the coxosternite, trochanteroprefemur, femur, and tibia to achieve a straight, free standing conformation of the tarsungulum (Fig. 1B, D–F). A force transducer (100 g capacity; FORT100, World Precision Instruments Inc., Sarasota, USA) was mounted to a motorized linear stepper motor (Physik Instrumente GmbH & Co. KG, Karlsruhe, Germany) and used to load the tip of the forcipule with a speed of 100 µm/s until material failure occurred (Fig. 1D–F). Loading was monitored via a stereomicroscope and immediately stopped upon breaking of the structure. The force–time-curves of the breaking process were recorded with AcqKnowledge 3.7.0 (BIOPAC Systems, Inc., Goleta, USA) and the breaking force (the highest force needed to break the structure) was extracted. Seven forcipules of freshly prepared specimens of *Lithobius forficatus* and six forcipules of freshly prepared *Cryptops hortensis* were used. Additionally, five forcipules of *Scutigera coleoptrata*, which were stored in 70% ethanol, were measured. These forcipules were rehydrated before the measurements with a droplet of distilled water and excess water was absorbed with filter paper. To calculate the breaking stress, the cross-sectional area of each tarsungulum at the point of breakage was determined. For this purpose, the samples were sputter coated with a 10 nm layer of gold–palladium and SEM micrographs of the broken sections were obtained with a Hitachi TM3000 SEM (Hitachi High-technologies Corp., Tokyo, Japan) at 15 kV. The cuticular area was measured using Fiji [48]. As the broken cuticle was not perfectly flat in all samples, the percentage area at the corresponding cross-section of the tarsungulum was additionally measured from microCT scans and used to calculate the actual cuticular area in the exposed cross-section after the experiment (Fig. 1C, C’). The breaking stress each sample endured was calculated with:

1 $$\sigma = \frac{F}{A},$$

where *F* is the loading force, *A* is the calculated cross-sectional area of the cuticle and *σ* is the resulting compressive stress the sample endured before damage.

Nanoindentation experiments were performed on forcipules of two specimens of *Lithobius forficatus* and *Cryptops hortensis*, respectively. The forcipules were attached to object glass slides using double-sided adhesive carbon tape and surrounded by a small metallic ring following the protocols of Krings et al. [51] and Gorb & Krings [52]. Each ring was filled with epoxy resin (Reckli Epoxy WST, RECKLI GmbH, Herne, Germany) covering the forcipules completely. The samples were kept at room temperature until polymerisation finished (~ 3 days). The glass slides with the carbon tape were removed and each sample was polished with sandpapers of different roughness until the cuticle section of the forcipule was on display. Finally, the surface was smoothed with aluminium oxide polishing powder suspension of 0.3 μm grain size (PRESI GmbH, Hagen, Germany) on a polishing machine (Minitech 233/333, PRESI GmbH, Hagen, Germany). As different regions of interest were at different levels of the sample, the sample was further polished until the next region of interest emerged after one round of nanoindentation.

For nanoindentation, a nanoindenter SA2 (MTS Nano Instruments Inc., Tennessee, USA), equipped with a Berkovich indenter tip and a dynamic contact module (DCM) head, was used. The mechanical properties, hardness (*H*) and Young’s modulus (*E*), were determined from force-distance curves by applying the continuous stiffness mode. Each indent and corresponding curve were manually controlled.

### Statistical analyses

Both breaking stress and the force required for breaking the samples were statistically compared in SigmaPlot 12.0 (Systat Software Inc., Delaware, USA) using two-tailed t-tests (α = 0.05). The data were tested for normality (Shapiro–Wilk-test) and homoscedasticity (Levenes’ test) beforehand and were both normally distributed (breaking stress: *p* = 0.819; breaking force: *p* = 0.241) and showed equal variance (breaking stress: *p* = 0.890; breaking force: *p* = 0.057). Hardness (*H*) and Young’s modulus (*E*) obtained from nanoindentation measurements were compared using Mann–Whitney rank sum tests in different combinations (α = 0.05), as the data were not normally distributed (Shapiro–Wilk-test) (*H*: *p* < 0.050; *E*: *p* < 0.050). We compared *H* and *E* between tarsungulum and the remaining podomeres for each species separately, as well as both *H* and *E* of the tarsungulum between the species. Correlation between *H* and *E* were tested for both species using Spearman’s rank correlation.

## Results

### Morphology and material composition of centipede forcipules and locomotory legs

#### *Scutigera* coleoptrata (*Scutigeromorpha*)

**Fig. 2:**
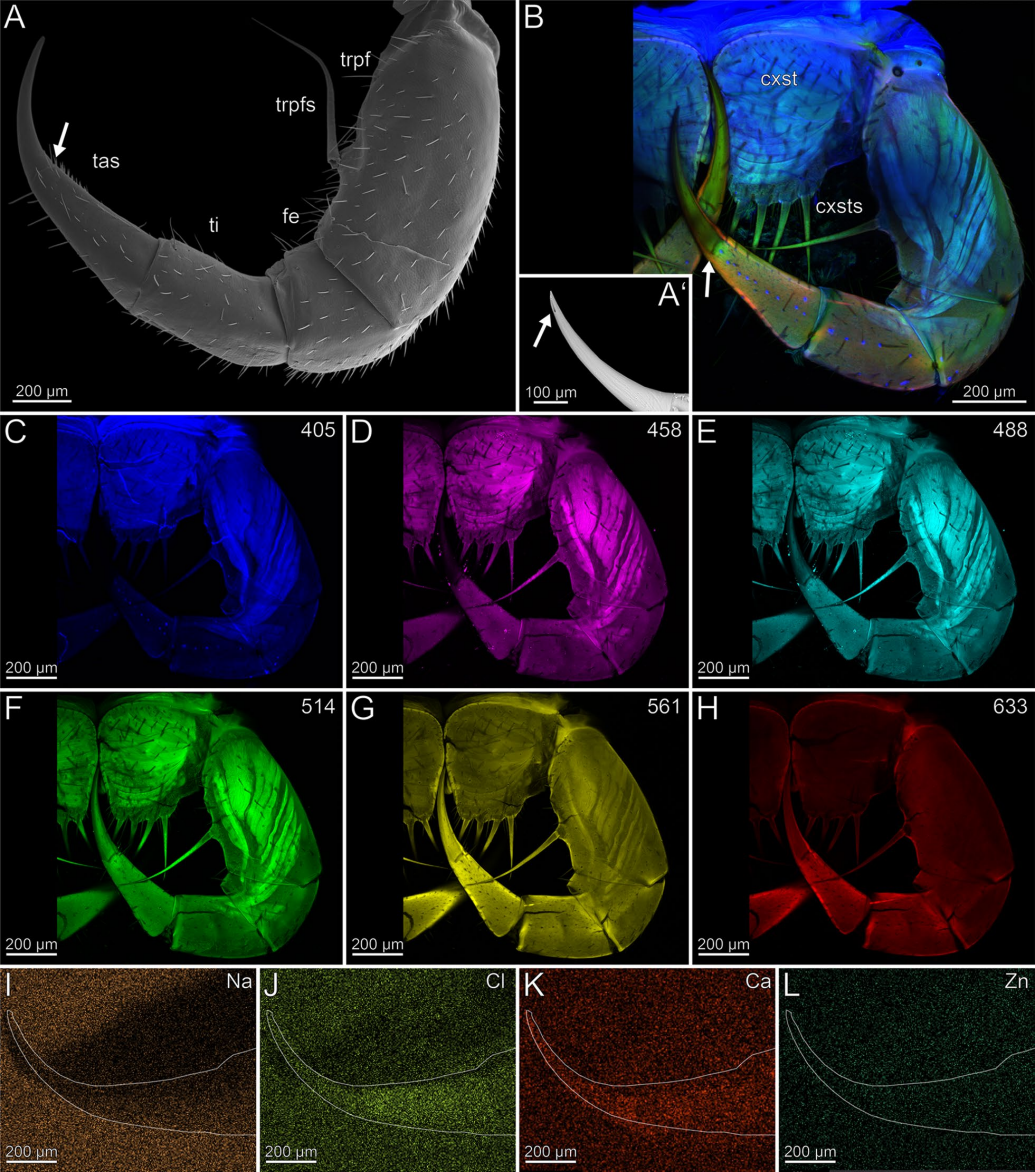
The forcipule of *Scutigera coleoptrata*. **A** Right forcipule in ventral view (SEM). Note the transverse suture of the tarsungulum (arrow). Trochanteroprefemoral spine is broken. **A’** Dorsolateral view of the tarsungulum. Arrow points at venom gland opening. Sensilla coeloconica present. **B** Maximum intensity projection overlay (CLSM) with excitations at 405 nm (blue), 514 nm (green) and 633 nm (red). Ventral view. The arrow points to the transverse suture. **C-H** Single channels of individual excitations. **C** Excitation at 405 nm. **D** Excitation at 458 nm. **E** Excitation at 488 nm. **F** Excitation at 514 nm.** G** Excitation at 561 nm.** H** Excitation at 633 nm.** I** EDX mapping of Na in the right tarsungulum in ventral view. The outline resembles the shape of the tarsungulum. **J** EDX mapping of Cl in the tarsungulum.** K** EDX mapping of Ca in the tarsungulum.** L** EDX mapping of Zn in the tarsungulum. **cxst** coxosternite. **cxsts** coxosternal spines. **fe** femur. **tas** tarsungulum. **ti** tibia. **trpfe** trochanteroprefemur. **trpfs** trochanteroprefemoral spine

The coxosternites of the forcipules are medially unfused (Fig. 2B–H). The proportions of the podomeres are approximately 3: 1: 1.5: 3 (trochanteroprefemur: femur: tibia: tarsungulum). All telopodal podomeres are slender and have greater length than width (except for the femur), with their joints situated more medially along the dorsoventral axis (Fig. 2A, B). A transverse suture is present around the middle of the tarsungulum (Fig. 2A–H). The forcipule is covered with longer trichoid sensilla, as well as short sensilla coeloconica at the distal tarsungulum (Fig. 2A). The venom gland opening is located at the tip of the tarsungulum, positioned subterminally on the dorsal side (Fig. 2A’). Among the podomeres, the femur has the thinnest cuticle (9.3 ± 2.7 µm). The tibia exhibits significant thickness variability, with the base (40.9 ± 10.4 µm) being considerably thicker than the distal region (15.2 ± 4.2 µm). The thickness of the cuticle of the tarsungulum gradually decreases towards its tip, ranging from a thicker cuticle at the base (14.8 ± 1.6 µm), to a thinner cuticle (10.0 ± 2.8 µm) at the tip. The cuticle of the podomeres has no distinct difference in radiodensity.

The forcipule exhibits a slight gradient of autofluorescence, increasing towards the distal podomeres and the tarsungulum, with the strongest autofluorescence present at the distal tip of the tarsungulum (Fig. 2B). The coxosternal and trochanteroprefemoral spines exhibit moderate autofluorescence without any gradient at different excitations. When excited at 405 nm, the coxosternite, trochanteroprefemur, and femur mostly emit moderate autofluorescence of the musculature and the pores of sensilla sockets in the cuticle; the tarsungulum does not emit any autofluorescence (Fig. 2C). Excitations at 458 and 488 nm result in weak autofluorescence in the tarsungulum (Fig. 2D, E). The musculature exhibits moderate autofluorescence at excitations at 458, 488 and 514 nm (Fig. 2D–F). The autofluorescence of the musculature is weak, when excited at 561 nm, and not detectable when excited at 633 nm (Fig. 2G, H). Excitation at 633 nm reveals moderate autofluorescence in the coxosternite, trochanteroprefemur, and femur, while the cuticle of the tibia and the tarsungulum emits strong autofluorescence. The joints exhibit moderate autofluorescence (Fig. 2B). In comparison to an ethanol-preserved specimen, the cuticle of a freshly fixed specimen exhibits more distinctive S, Cl, and P intensities.

In both specimens, S and P are more concentrated at the joint membranes, while Na, Cl and Ca are present without any concentration hotspots or gradients (Fig. 2I–K). Zn is absent in the forcipule (Fig. 2L).

**Fig. 3:**
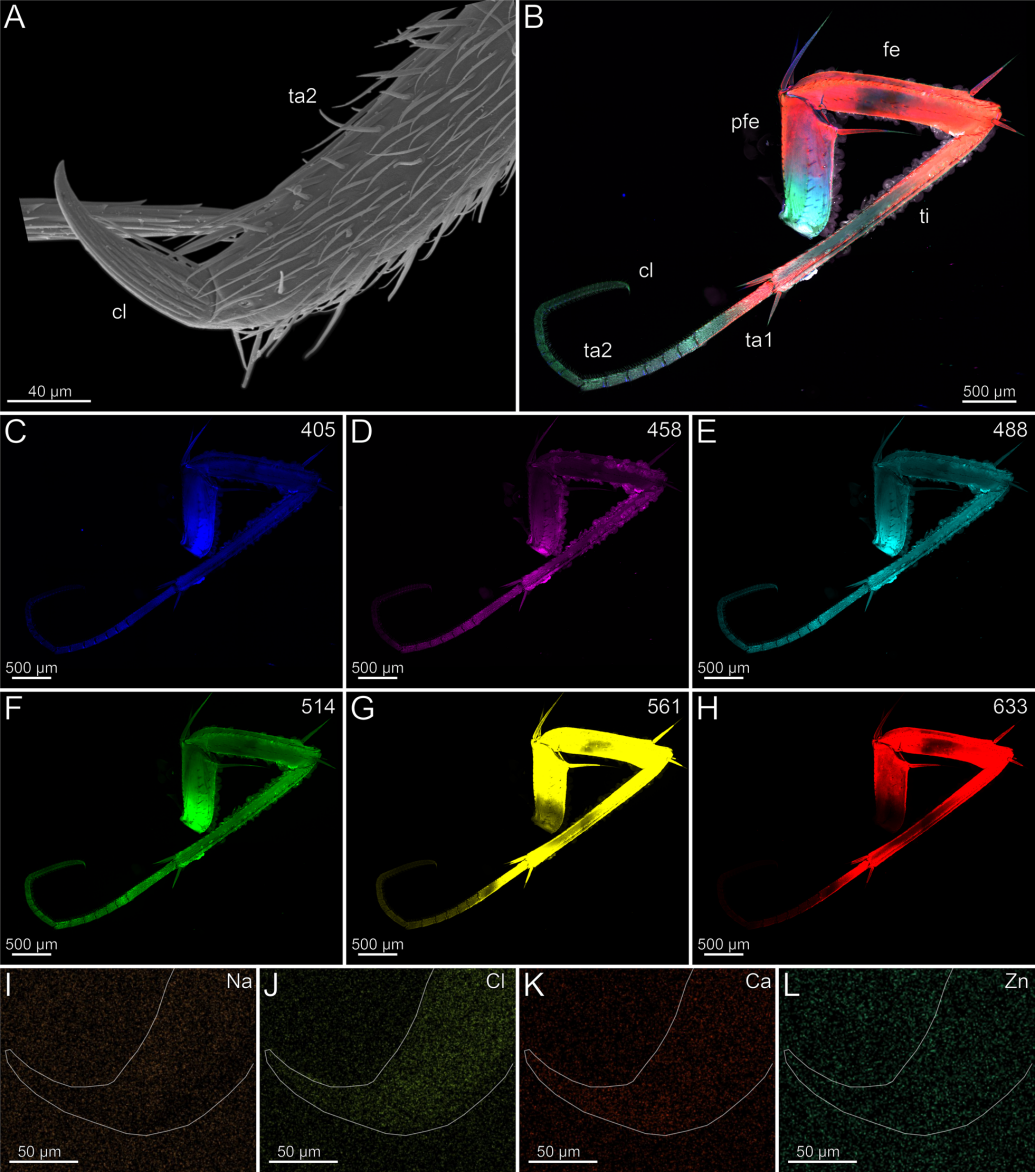
The locomotory leg 10 of *Scutigera coleoptrata*. **A** Distal tip of tarsus 2 and pretarsal claw in ventrolateral view (SEM). **B** Maximum intensity projection overlay (CLSM) with excitations at 405 nm (blue), 458 nm (purple), 488 nm (cyan), 561 nm (yellow) and 633 nm (red). **C-H** Single channels for individual excitations. **C** Excitation at 405 nm. **D** Excitation at 458 nm. **E** Excitation at 488 nm. **F** Excitation at 514 nm.** G** Excitation at 561 nm.** H** Excitation at 633 nm.** I** EDX mapping of Na in tarsus 2 and the pretarsal claw. The outline resembles the shape of the structure. **J** EDX mapping of Cl in tarsus 2 and the pretarsal claw.** K** EDX mapping of Ca in tarsus 2 and the pretarsal claw.** L** EDX mapping of Zn in tarsus 2 and the pretarsal claw. **cl** pretarsal claw. **fe** femur. **pfe** prefemur. **ta1** tarsus 1. **ta2** tarsus 2. **ti** tibia

In the locomotory leg 10, the tarsi are elongated and composed of multiple annuli (Fig. 3B). They exhibit a high density of trichoid sensilla, with prominent resilient sole-hairs found distomedially along the tarsus 2. The distal tip of tarsus 2 is associated with the pretarsal claw (Fig. 3A). The locomotory leg does not exhibit an autofluorescence gradient, but rather autofluorescence spots (Fig. 3B–H). When excited at 405 nm, the proximal part of the prefemur emits weaker autofluorescence compared to the stronger autofluorescence from femur, tibia, and the proximal tarsus 1 (Fig. 3C). Podomeres excited at 458, 488 and 514 nm exhibit an almost uniform moderate autofluorescence (Fig. 3D–F). When excited at 488 and 514 nm, the pretarsal claw and tarsal annuli emit moderate autofluorescence. The tarsus 2 emits less autofluorescence from proximal to distal. Excitations at 561 and 633 nm result in strong autofluorescence near the joints and moderate autofluorescence in the middle of the femur and tibia (Fig. 3G, H). Ca and Cl are present at low concentrations and without a concentration gradient. Zn is absent (Fig. 3I–L). Na, Cl and Ca are present throughout the sample with no localisation hotspot (Fig. 3I–K). The concentration of Na and Cl is higher in a freshly fixed locomotory leg 10 compared to an ethanol-preserved specimen. There are no distinct differences in elemental concentrations between locomotory leg 10 and the forcipule.

#### *Lithobius* forficatus (*Lithobiomorpha*)

**Fig. 4:**
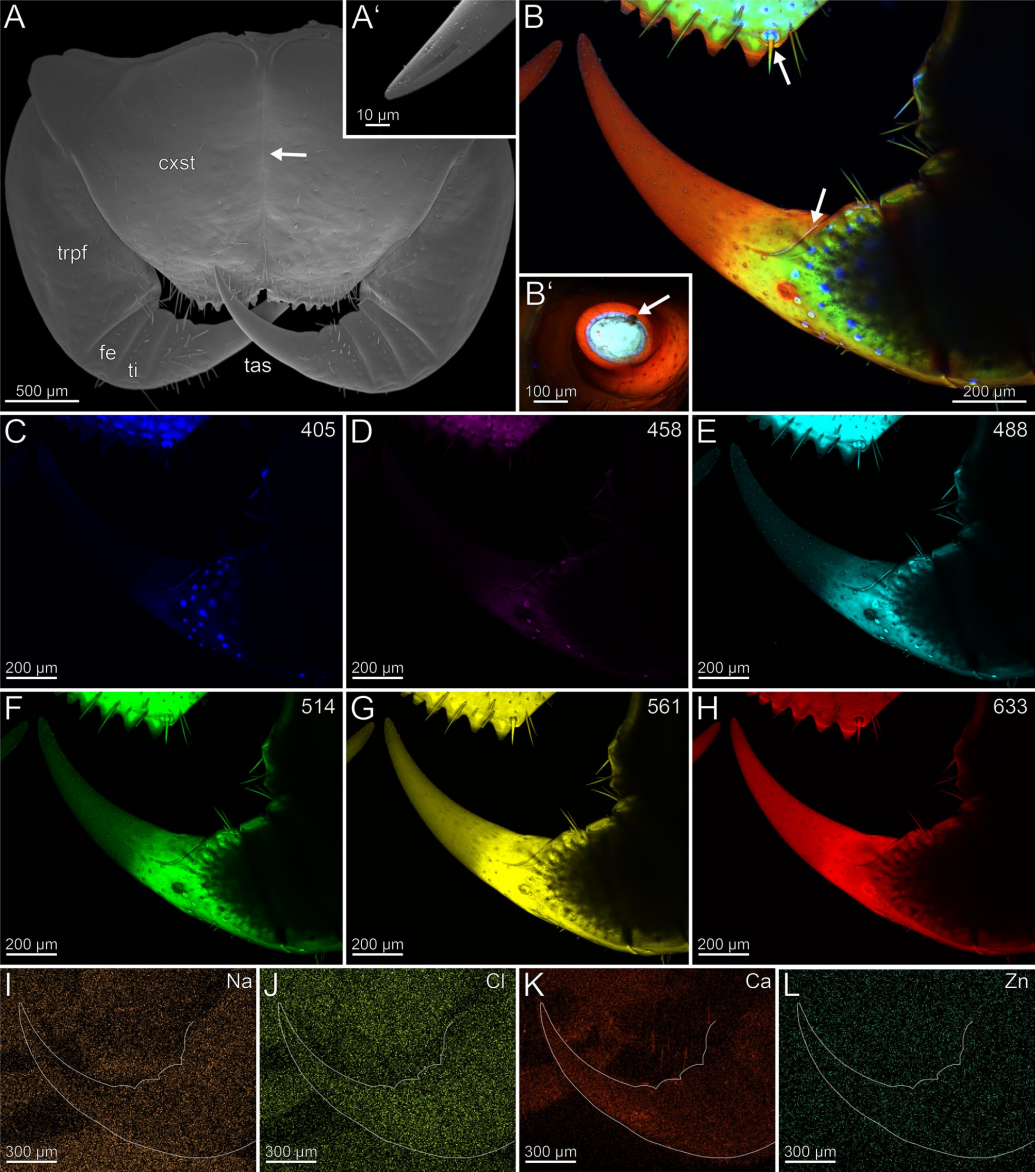
Forcipule of *Lithobius forficatus*. **A** Forcipule in ventral view (SEM). The arrow points to the coxosternal suture. **A’** Tip of the tarsungulum with venom gland opening. Dorsolateral view. **B** Maximum intensity projection overlay (CLSM) excited at 405 nm, 458 nm, 514 nm and 633 nm. Ventral view. Sensilla coeloconica present. One arrow points to the tarsungular suture and one to the porodont at the forcipular dental margin. **B’** Maximum intensity projection overlay (CLSM) of a cut tarsungulum excited at 405 nm, 488 nm, 555 nm and 639 nm. Frontal view. The arrow points to the venom channel. **C** Excitation at 405 nm. **D** Excitation at 458 nm. **E** Excitation at 488 nm. **F** Excitation at 514 nm.** G** Excitation at 561 nm.** H** Excitation at 633 nm.** I** EDX mapping of Na in the right forcipule in ventral view. The outline resembles the shape of the structure. **J** EDX mapping of Cl in the forcipule.** K** EDX mapping of Ca in the forcipule.** L** EDX mapping of Zn the forcipule. **cxst** coxosternite. **fe** femur. **tas** tarsungulum. **ti** tibia. **trpfe** trochanteroprefemur

The coxosternites of the forcipule are fused featuring a longitudinal median sutural ridge (Fig. 4A). The proportions of the podomeres are approximately 5: 1: 1: 3.3 (trochanteroprefemur: femur: tibia: tarsungulum). The trochanteroprefemur and tarsungulum are longer than wide, while the femur and tibia are wider than long. Each podomere is connected to a joint at both the anterior and posterior ends, positioned distolaterally. The forcipule is sparsely covered with sensilla trichodea. Only the distal tarsungulum possesses sensilla coeloconica. The venom gland opening is located at the tip of the tarsungulum, positioned subterminally on the dorsal side (Fig. 4A, A’). The radiodensity of the tarsungulum’s cuticle varies due to the presence of an inner and outer cuticular layer in the tip area (Fig. 1C, C’). The inner layer is twice as radiodense as the outer, although slightly less radiodense than the cuticle of the other podomeres. The outer layer is slightly less radiodense than the interpodomeric membranes, which are less less radiodense than the cuticle of the podomeres. The cuticle of coxosternite, trochanteroprefemur, femur, and tibia exhibit no distinct differences in radiodensity. There are no noticeable differences in cuticle thickness between trochanteroprefemur (28.4 ± 5.8 µm), femur (30.5 ± 7.2 µm), tibia (25.6 ± 2.5 µm), and tarsungulum (32.2 ± 3.8 µm). The cuticle of the tip of the tarsungulum (33.6 ± 9.1 µm) is not distinctly thicker than at its base.

**Fig. 5:**
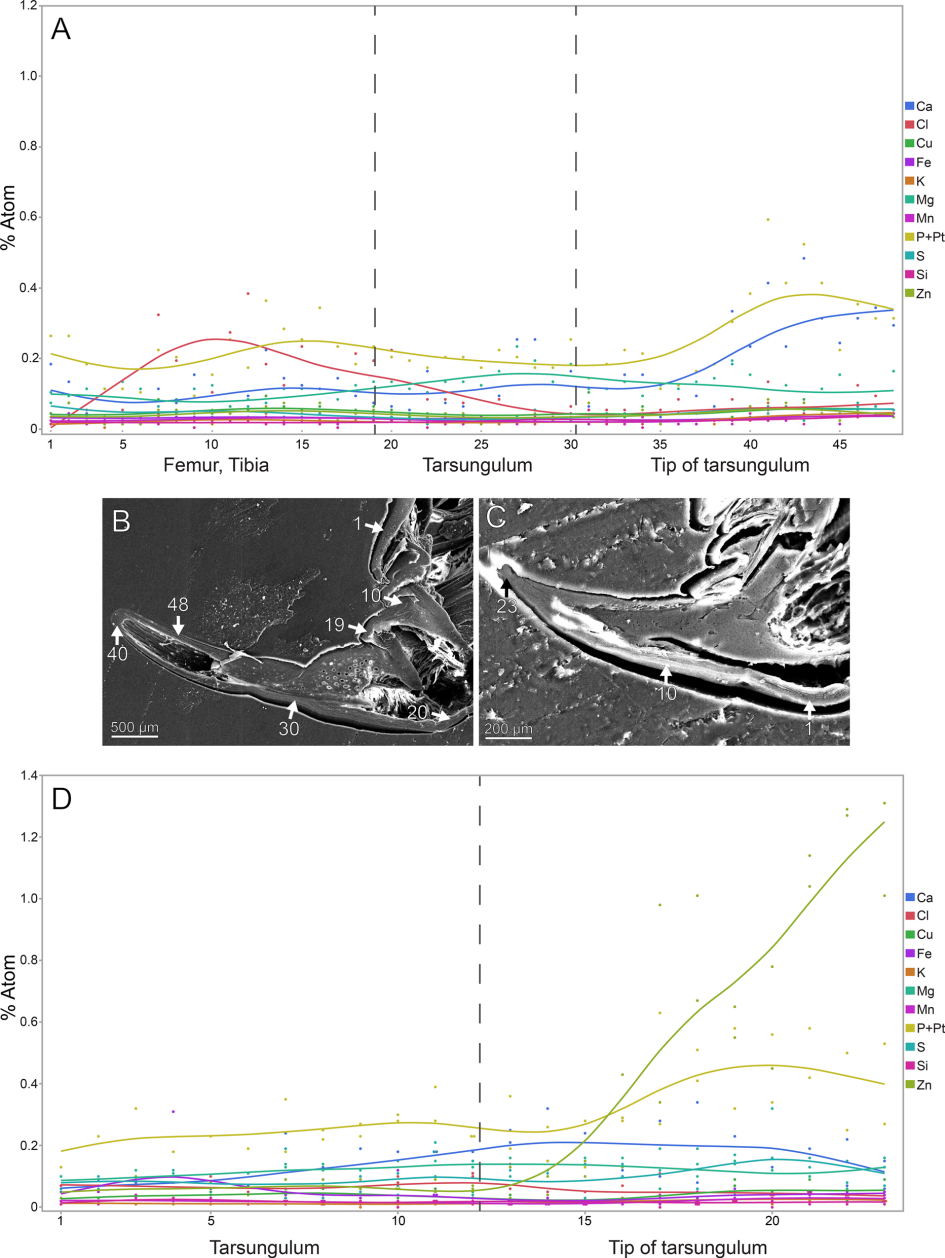
Results from EDX point measurements of the forcipular cuticle of *Lithobius forficatus* and *Cryptops hortensis*. **A** EDX results from the femur, tibia and tarsungulum of *L. forficatus* in atomic %. **B** Polished and smoothened longitudinal section of the forcipule of *L. forficatus* (SEM). Arrows and numbers indicate the locality of point measurements. **C** Polished and smoothened longitudinal section of the forcipule of *C. hortensis* (SEM). Arrows and numbers indicate the locality of point measurements. **D** EDX results from the tarsungulum of *C. hortensis* in atomic %

The forcipule exhibits an autofluorescence gradient, increasing towards the tarsungulum, with the strongest autofluorescence in the distal half of the tarsungulum (Fig. 4B). The cuticle of the lateral coxosternite, median trochanteroprefemur, and femur does not emit autofluorescence when excited (Fig. 4B–H). When excited at 405, 488, 555 and 639 nm, a cross-section of the tarsungulum, the venom channel and the cuticle emit strong autofluorescence, indicating a strong sclerotization (Fig. 4B’). When excited at 405 nm, the coxosternal membrane, the longitudinal median sutural ridge, the interpodomeric membrane between the trochanteroprefemur and femur, and the pores of sensilla sockets on the proximal tarsungulum and anterior coxosternite emit weak autofluorescence (Fig. 4C). When excited at 458, 488 and 514 nm, the coxosternite and the proximal part of the tarsungulum emit moderate autofluorescence (Fig. 4D–F). Excitation at 561 and 633 nm leads to strong autofluorescence in the tarsungulum (Fig. 4G, H). In the whole forcipule, Ca is present with a gradient of increasing concentration towards the tip of the tarsungulum (Fig. 4K). EDX point measurements additionally support a Ca gradient on the tarsungulum and a corresponding gradient of P + Pt (Fig. 5A, B). Additionally, an increased Cl concentration is present in some cuticle parts of femur and tibia. Other elements with concentration spots or gradients are absent (Fig. 4I–L).

**Fig. 6:**
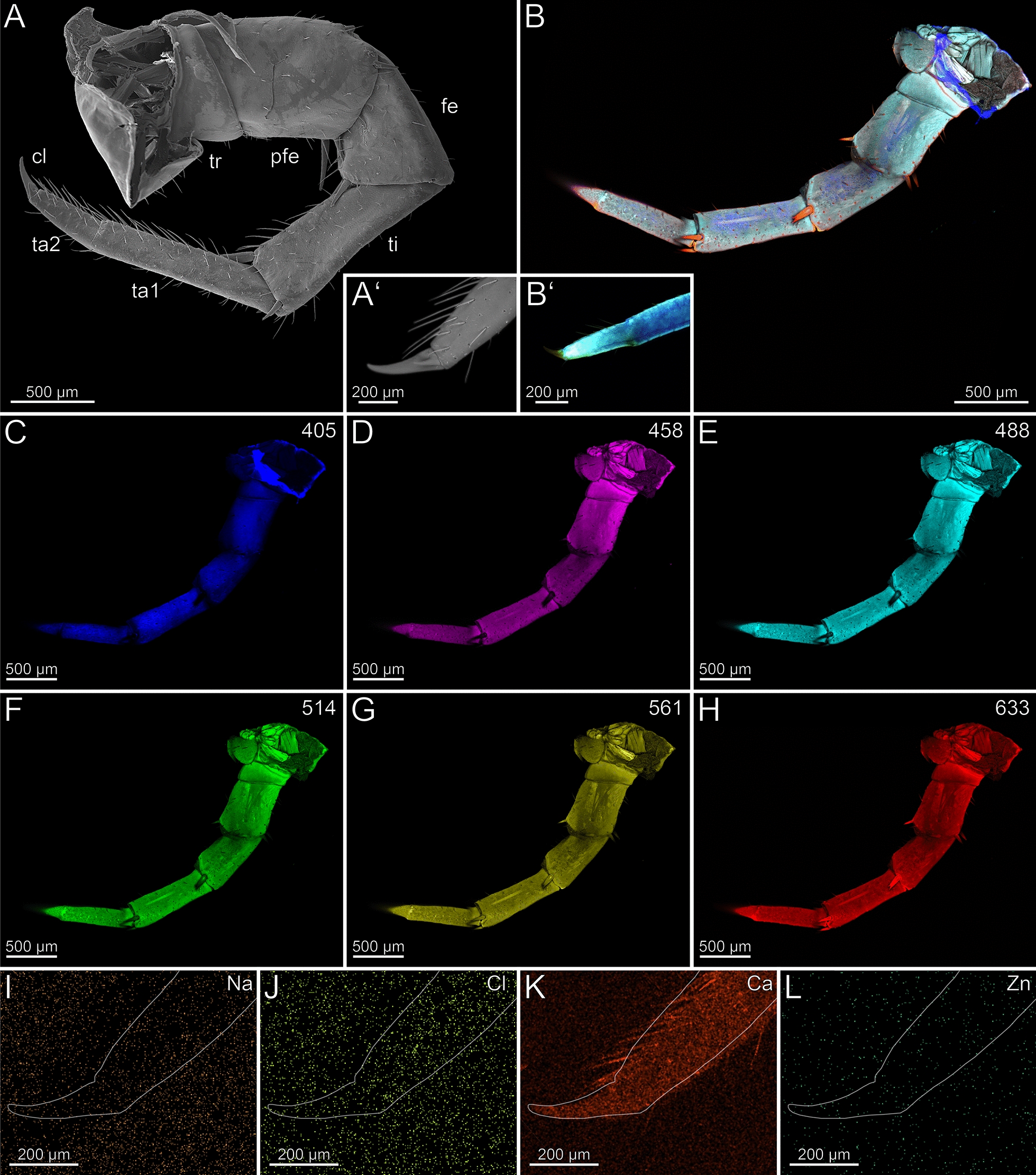
Locomotory leg 10 of *Lithobius forficatus*. **A** Locomotory leg 10 in posterior view (SEM). **A’** Pretarsal claw and tarsus 2 in posterior view. **B** Maximum intensity projection overlay (CLSM) excited at 405 nm, 488 nm, 514 nm, 561 nm and 633 nm. Lateral view. **B’** Maximum intensity projection overlay of the tarsus 1, tarsus 2 and pretarsal claw excited at 405 nm, 458 nm, 488 nm, 514 nm, 561 nm and 633 nm. Ventrolateral view. **C** Excitation at 405 nm. **D** Excitation at 458 nm. **E** Excitation at 488 nm. **F** Excitation at 514 nm.** G** Excitation at 561 nm.** H** Excitation at 633 nm.** I** EDX mapping of Na in tarsus 1, tarsus 2 and pretarsal claw in ventrolateral view. The outline resembles the shape of the structure. **J** EDX mapping of Cl in tarsus 1, tarsus 2 and pretarsal claw.** K** EDX mapping of Ca in tarsus 1, tarsus 2 and pretarsal claw.** L** EDX mapping of Zn in tarsus 1, tarsus 2 and pretarsal claw. **cl** pretarsal claw. **fe** femur. **pfe** prefemur. **ta1** tarsus 1. **ta2** tarsus 2. **ti** tibia. **tr** trochanter

In the locomotory leg 10, sensilla trichodea are present on all podomeres, except the pretarsal claw, where sensilla coeloconica are present (Fig. 6A, A’). The locomotory leg does not exhibit autofluorescence gradients (Fig. 6B, B’). Excitation at 405, 458, 488 and 514 nm results in moderate autofluorescence throughout the podomeres, excluding the pretarsal claw (Fig. 6C–F). When excited at 405, 458, 488 and 514 nm, the pores of sensilla sockets in the cuticle emit autofluorescence, which indicates that these regions are weakly to moderately sclerotized (Fig. 6C–F). When excited at 561 and 633 nm, the pretarsal claw emits strong autofluorescence (Fig. 6G, H); the spurs of the prefemur, femur, and tibia emit moderate autofluorescence (Fig. 6B, B’). Na, Cl and Zn are absent in the locomotory leg 10 (Fig. 6I, J, L). Compared to other elements, Ca is present in a higher concentration throughout the cuticle (Fig. 6K). There are no distinct differences in elemental concentrations between locomotory leg 10 and the forcipule.

#### *Craterostigmus* tasmanianus (*Craterostigmomorpha*)

**Fig. 7:**
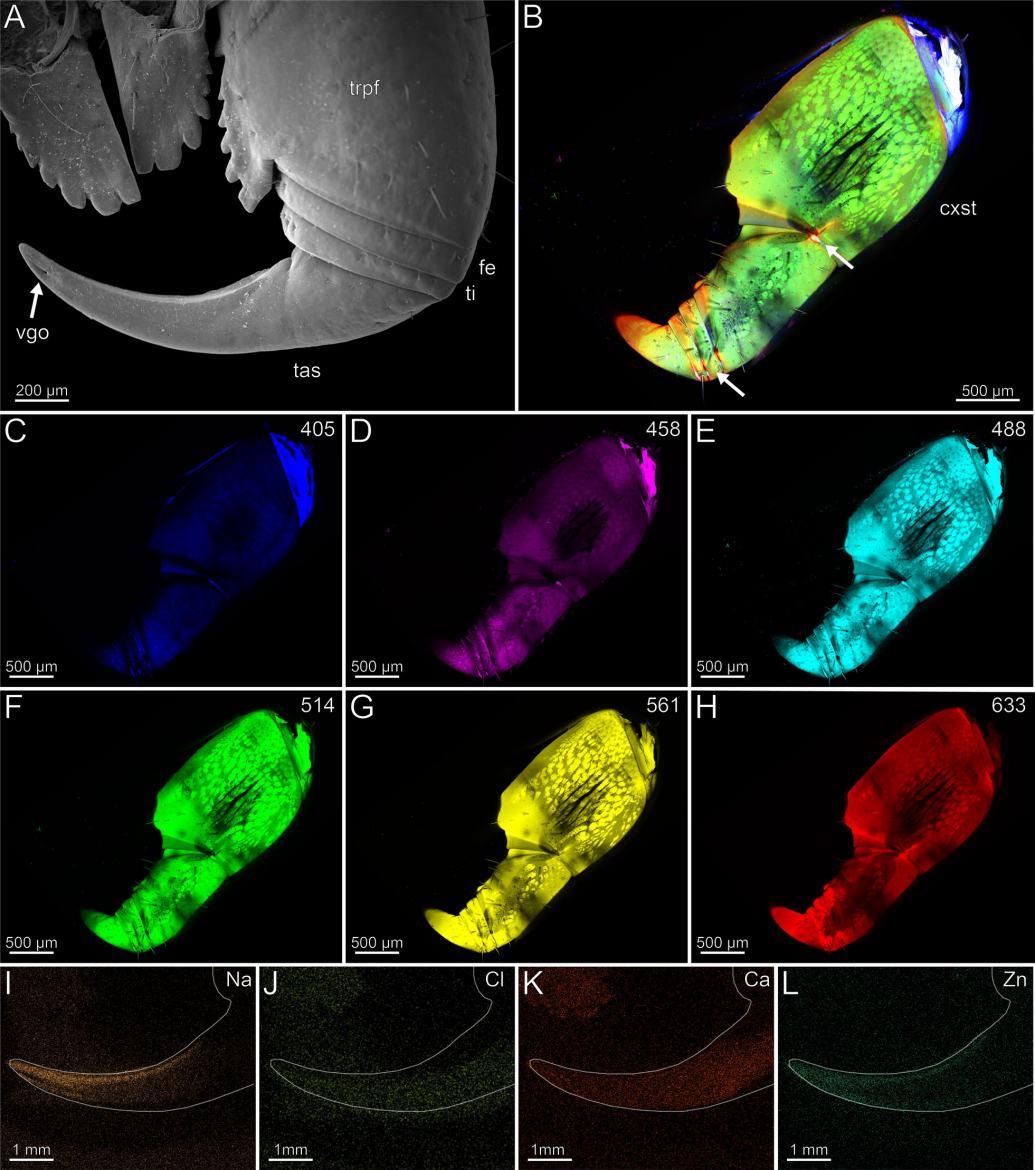
Forcipule of *Craterostigmus tasmanianus*. **A** Right forcipule in dorsal view (SEM). Sensilla coeloconica present. **B** Maximum intensity projection overlay (CLSM) excited at 405 nm, 458 nm, 514 nm and 633 nm. Ventral view. The arrows point to the joints. **C** Excitation at 405 nm. **D** Excitation at 458 nm. **E** Excitation at 488 nm. **F** Excitation at 514 nm.** G** Excitation at 561 nm.** H** Excitation at 633 nm.** I** EDX mapping of Na in the right tarsungulum in dorsal view. The outline resembles the shape of the structure. **J** EDX mapping of Cl in the right tarsungulum.** K** EDX mapping of Ca in the right tarsungulum.** L** EDX mapping of Zn in the right tarsungulum. **cxst** coxosternite. **fe** femur. **tas** tarsungulum. **ti** tibia. **trpfe** trochanteroprefemur. **vgo** venom gland opening

The coxosternite is characterised by an anteromedian serrated protrusion with seven teeth that increase in size distally, as well as by the presence of a median suture (Fig. 7A). The proportions of the podomeres are approximately 10: 1: 1: 10 (trochanteroprefemur: femur: tibia: tarsungulum). The podomeres possess far lateral joints, while the joints between coxosternite and trochanteroprefemur are positioned centrally (Fig. 7A). The trochanteroprefemur is equipped with a medial serrated protrusion bearing flat teeth (Fig. 7A). The forcipule is sparsely covered with sensilla trichodea. Only at the distal tarsungulum sensilla coeloconica are present. The venom gland opening is located dorsally and subterminally on the tarsungulum (Fig. 7A). The cuticle of the trochanteroprefemur (32.7 ± 7.8 µm) and tibia (36.1 ± 8.3 µm) are thinner than the cuticle of the tarsungulum (46.6 ± 7.4 µm) The femur (56.5 ± 21 µm) has the thickest cuticle among the podomeres and gets thinner towards adjacent podomeres. The tip of the tarsungulum (43.5 ± 10.3 µm) is thinner than its base. There is no distinct difference in radiodensity between the cuticle of trochanteroprefemur, femur, tibia and the proximal tarsungulum. The cuticle of the tip of the tarsungulum is, however, less radiodense than the rest of the tarsungulum.

**Fig. 8:**
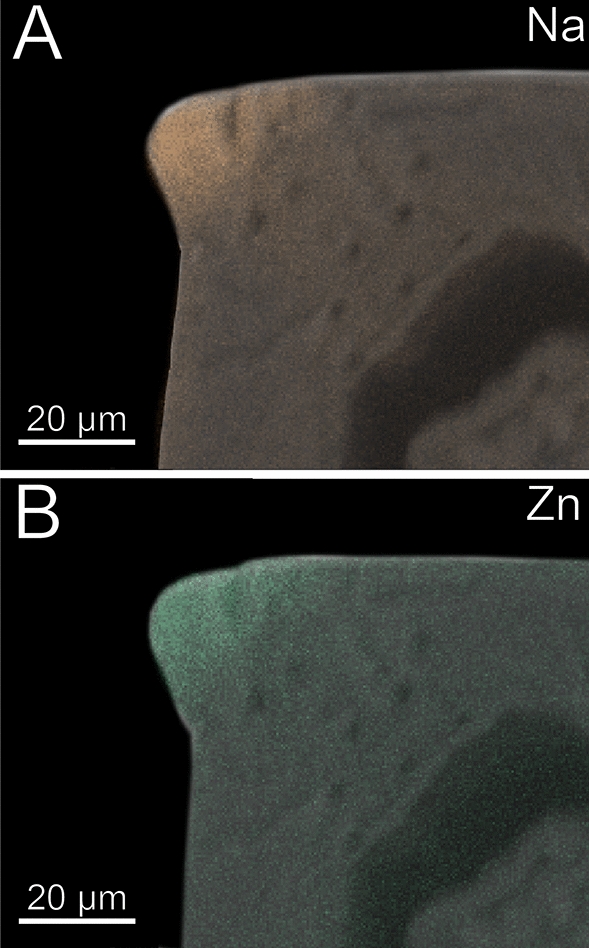
Cross-section of the tarsungulum of *Craterostigmus tasmanianus*. Frontal view. **A** EDX mapping of Na. **B** EDX mapping of Zn

The forcipule exhibits an autofluorescence gradient, increasing towards the distal podomeres and the tarsungulum (Fig. 7B). The distal part of the tarsungulum does not emit any autofluorescence when excited. The membrane of the coxosternite emits weak autofluorescence, when excited at 405, 458, 488 and 514 nm (Fig. 7C–F) and is thus weakly to moderately sclerotized. Parts of the interpodomeric membranes between the coxosternite and trochanteroprefemur, femur and tibia, and tibia and tarsungulum emit autofluorescence when excited at 405, 458 and 488 nm (Fig. 7C–E). When excited at 405, 458 and 488 nm, the pores of sensilla sockets in the cuticle emit moderate autofluorescence as well. All podomeres emit moderate autofluorescence when excited at 488, 514 and 561 nm, which points to a moderate sclerotization (Fig. 7E–G). When excited at 488, 514 and 561 nm, the proximal tarsungulum emits weak autofluorescence. When excited at 488 nm, parts of the musculature within the coxosternite and trochanteroprefemur emit moderate autofluorescence. The median joint between the coxosternite and trochanteroprefemur, the distal parts of the trochanteroprefemur, femur, and tibia, as well as the mediodistal part of the tarsungulum, emit strong autofluorescence when excited at 633 nm (Fig. 7H). In the forcipule, Ca and Cl is present throughout the cuticle, but no gradient was detected (Fig. 7K). In the tarsungulum, Na and Zn gradients are present on the ventral margin of the tarsungulum, starting at two-thirds of its length (Fig. 7I, L). In cross-section of the tarsungulum, Na and Zn are concentrated at both the median and inner margins, reaching a depth of 20 μm inside the cuticle (Fig. 8A, B).

**Fig. 9:**
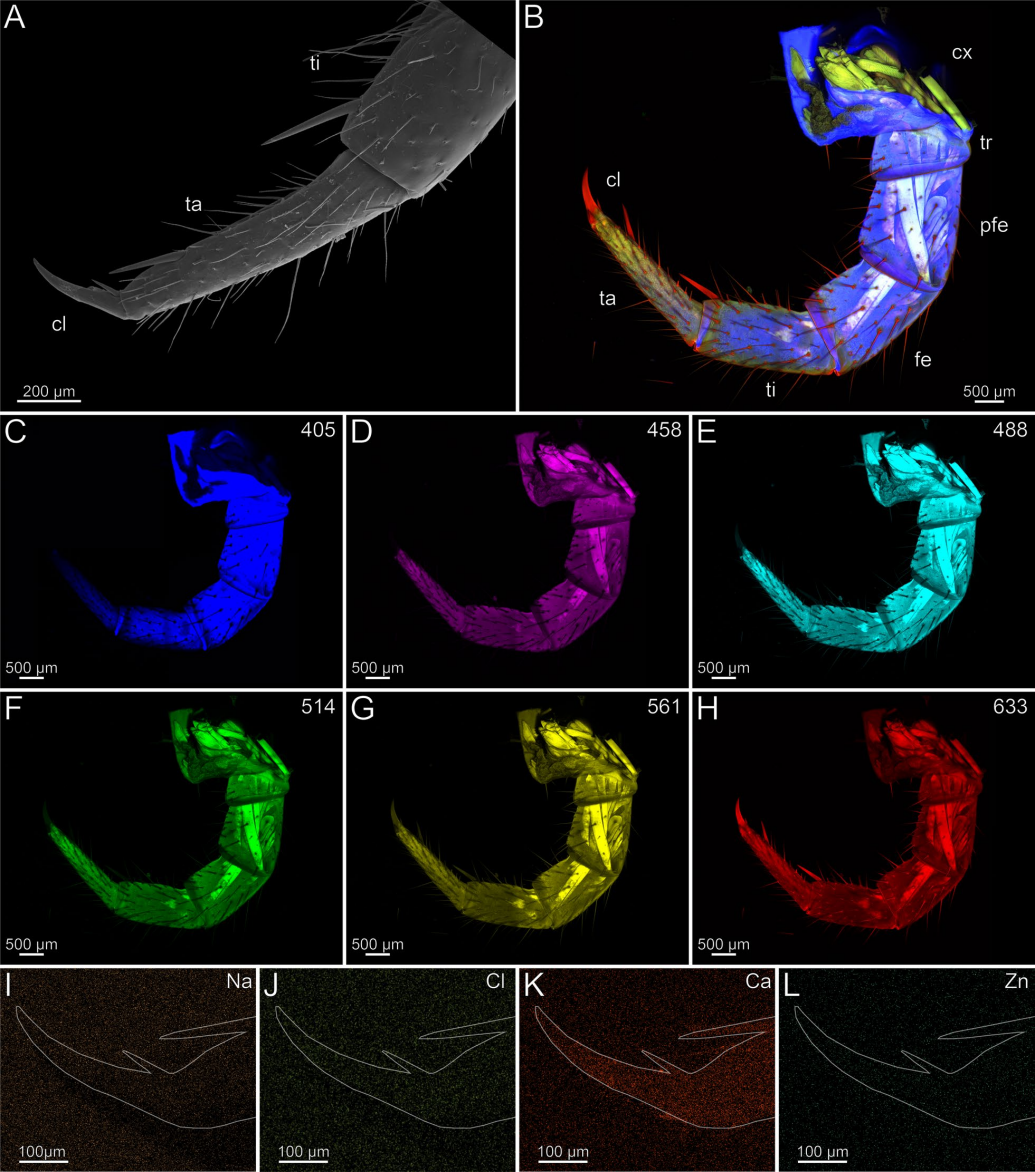
Locomotory leg 10 of *Craterostigmus tasmanianus*. **A** Locomotory leg 10 in lateral view (SEM). **B** Maximum intensity projection overlay (CLSM) excited at 405 nm, 458 nm, 488 nm, 514 nm, 561 nm and 633 nm. Lateral view. **C** Excitation at 405 nm. **D** Excitation at 458 nm. **E** Excitation at 488 nm. **F** Excitation at 514 nm.** G** Excitation at 561 nm.** H** Excitation at 633 nm.** I** EDX mapping of Na in tarsus and pretarsal claw in lateral view. The outline resembles the shape of the structure. **J** EDX mapping of Cl in tarsus and pretarsal claw.** K** EDX mapping of Ca in tarsus and pretarsal claw.** L** EDX mapping of Zn in tarsus and pretarsal claw. **cl** pretarsal claw. **cx** coxa. **fe** femur. **pfe** prefemur. **ta** tarsus. **ti** tibia. **tr** trochanter

In the locomotory leg 10, sensilla trichodea are present on all podomeres, except the pretarsal claw, where sensilla coeloconica are present (Fig. 9A). Leg 10 exhibits a moderate autofluorescence gradient from the tibia towards the distal part of the tarsus (Fig. 9B). When excited at 405 nm, strong autofluorescence in the coxa, trochanter, prefemur, femur, tibia, and the interpodomeric membranes is present (Fig. 9C). Excitation at 458, 488, 514 and 561 nm results in moderate autofluorescence (Fig. 9D–G). The musculature in the coxa, trochanter, prefemur, and tibia emits moderate autofluorescence when excited at 458, 488, 514 and 561 nm (Fig. 9D–G). Excitation at 633 nm results in strong autofluorescence of trichoid sensilla including socket pores, spurs, and the pretarsal claw, which indicates that these regions are strongly sclerotized (Fig. 9B, H). Na, Cl and Zn are absent in the locomotory leg 10 (Fig. 9I, J, L). Ca is present throughout the cuticle without a concentration gradient (Fig. 9K).

#### Scolopendra oraniensis and S. morsitans (*Scolopendromorpha*)

**Fig. 10:**
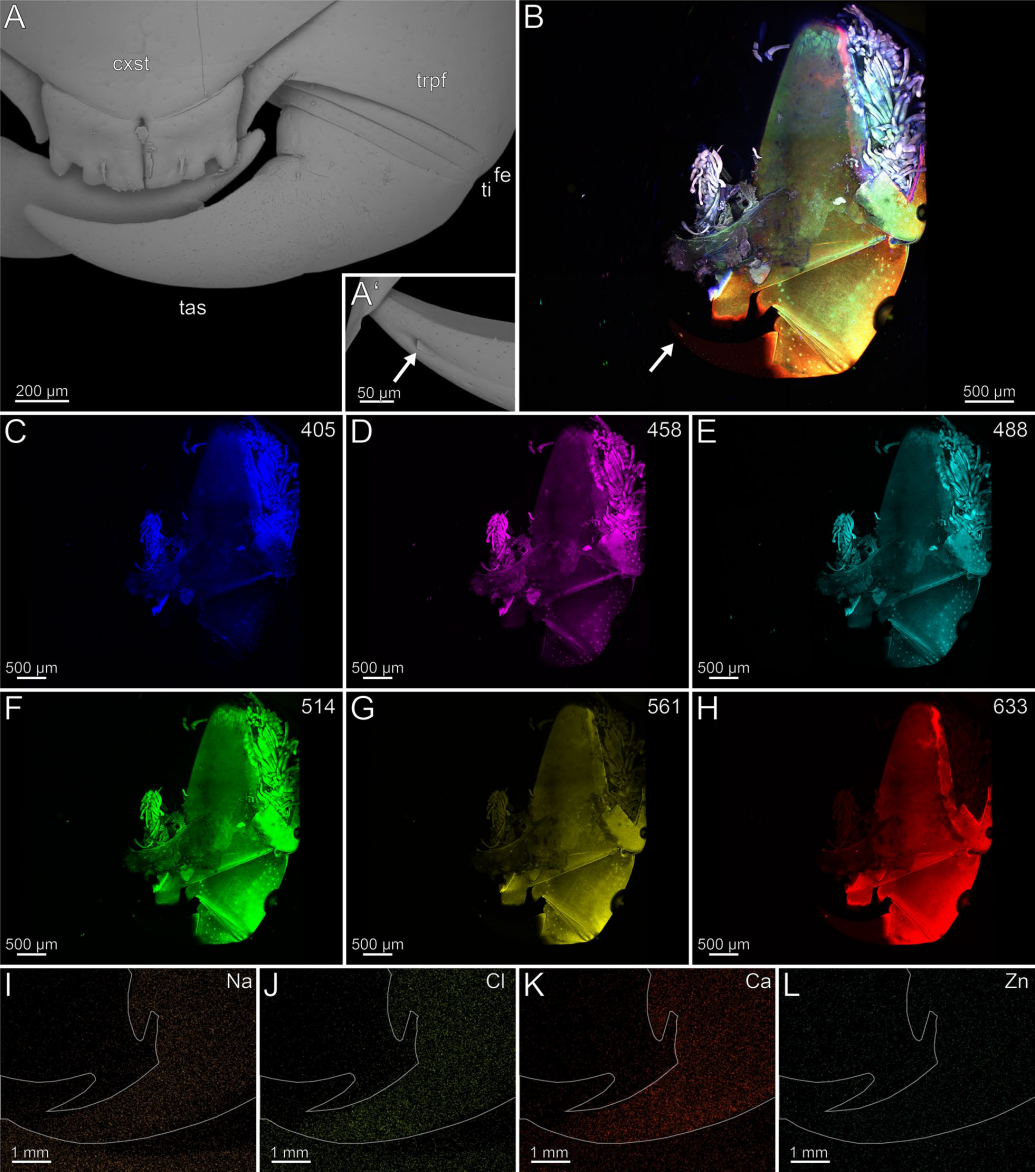
Forcipule of *Scolopendra oraniensis.* **A** Forcipule in ventral view (SEM). **A’** Tip of the tarsungulum with venom gland opening (arrow). Dorsal view. Sensilla coeloconica present. **B** Maximum intensity projection overlay (CLSM) excited at 405 nm, 458 nm, 514 nm and 633 nm. Ventral view. The arrow points to the venom gland opening. **C** Excitation at 405 nm. **D** Excitation at 458 nm. **E** Excitation at 488 nm. **F** Excitation at 514 nm.** G** Excitation at 561 nm.** H** Excitation at 633 nm.** I** EDX mapping of Na in the forcipule in dorsal view. The outline resembles the shape of the structure. **J** EDX mapping of Cl in the forcipule.** K** EDX mapping of Ca in the forcipule.** L** EDX mapping of Zn in the forcipule. **cxst** coxosternite. **fe** femur. **tas** tarsungulum. **ti** tibia. **trpfe** trochanteroprefemur

The coxosternites are medially fused without featuring a median suture (Fig. 10A). The coxosternite extends into an anteromedian protrusion with teeth. The proportions of the podomeres are approximately 20: 1: 1: 20 (trochanteroprefemur: femur: tibia: tarsungulum). The trochanteroprefemur is as wide as long, while femur and tibia are wider than long. The trochanteroprefemur is equipped with a median protrusion, and the trochanteroprefemur and the tarsungulum form a far laterally located shared joint (Fig. 10A). The forcipule is sparsely covered with sensilla trichodea. Only the distal tarsungulum possesses sensilla coeloconica. The venom gland opening is located subterminally and opens dorsal (Fig. 10A’). In *Scolopendra morsitans*, the cuticle of the trochanteroprefemur (41.3 ± 11.4 µm), tibia (23.1 ± 3.2 µm) and femur (25.9 ± 2.3 µm) are thinner than the cuticle of the tarsungulum (70.9 ± 7.8 µm). The tip of the tarsungulum (61.9 ± 7.0 µm) is slightly thinner than the base. There is no distinct difference in radiodensity between the cuticle of trochanteroprefemur, femur, tibia and the proximal tarsungulum. However, the cuticle of the tip of the tarsungulum is less radiodense than the cuticle of other podomeres.

The forcipule of *S. oraniensis* exhibits a moderate autofluorescence gradient from the base of the coxosternite to the femur, as well as strong autofluorescence in the tarsungulum (Fig. 10B). When excited at 405 and 458 nm, the forcipule emits weak autofluorescence (Fig. 10C). When excited at 405, 458, 488 and 514 nm, the pores of sensilla sockets in the cuticle and the exposed musculature at the base of the coxosternite emit weak autofluorescence (Fig. 10C–F). Excitation at 514 and 561 nm results in moderate autofluorescence in the cuticle of all podomeres, except the distal half of the tarsungulum (Fig. 10F, G). Excitation at 633 nm results in strong autofluorescence of the coxosternite, trochanteroprefemur, femur, tibia, and the proximal part of the tarsungulum (Fig. 10H). The distal part of the tarsungulum displays only weak autofluorescence when excited at 633 nm (Fig. 10H). Na, Cl and Ca are present throughout the cuticle, without a concentration gradient (Fig. 10I–K). Zn is absent (Fig. 10L). No distinct differences are present between an ethanol-preserved specimen and two freshly fixed specimens (one kept in captivity for over a year and the other caught in the wild and not fed until fixation). Compared to the ethanol-fixed specimen, in both freshly fixed specimens a slightly increased concentration of Na is present.

**Fig. 11:**
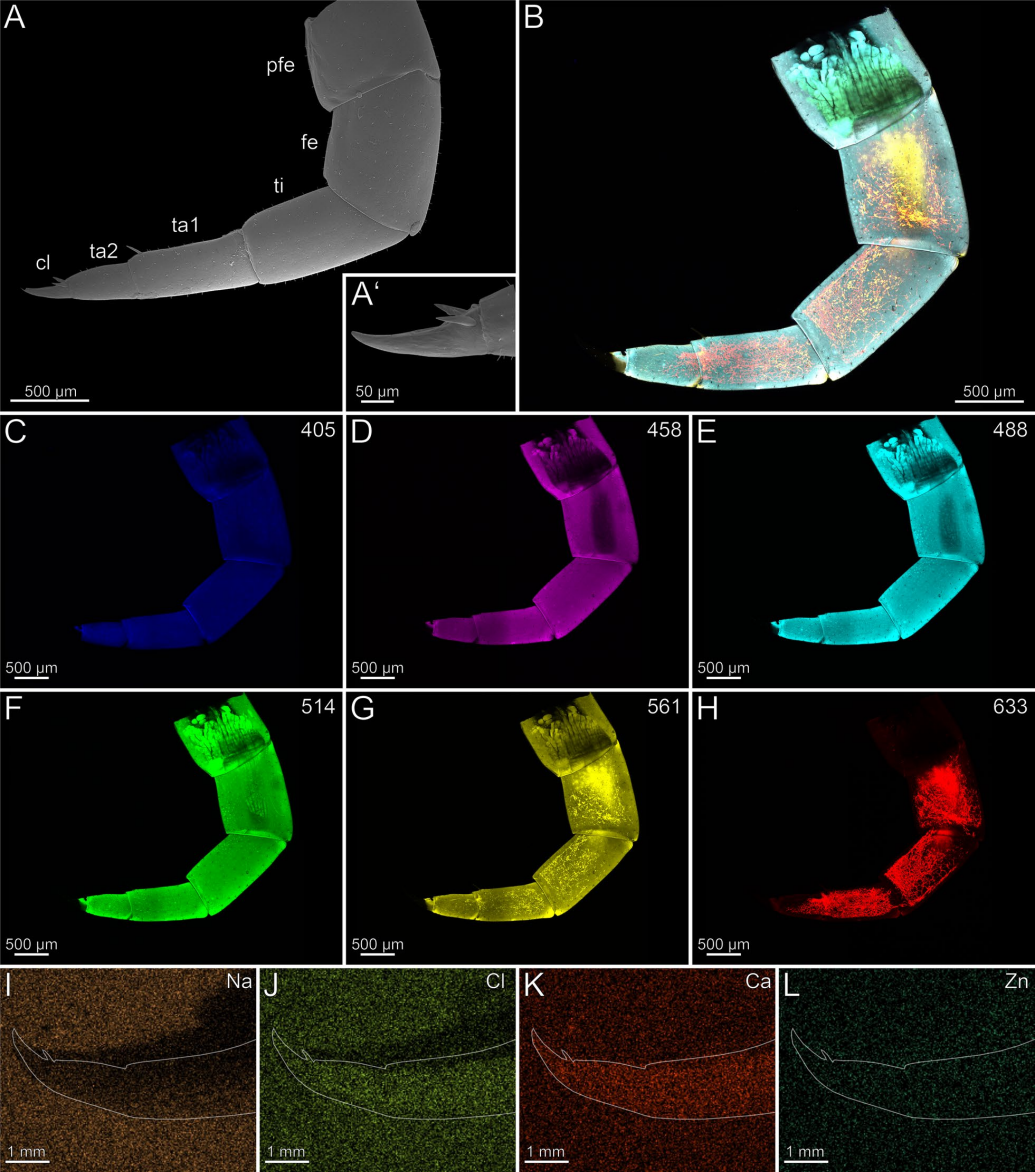
Locomotory leg 10 of *Scolopendra oraniensis ***A** Locomotory leg 10 in lateral view (SEM). **A’** Pretarsal claw and tarsus 2. Lateral view. Sensilla coeloconica present. **B** Maximum intensity projection overlay (CLSM) excited at 405 nm, 458 nm, 488 nm, 514 nm, 561 nm and 633 nm. Lateral view. **C** Excitation at 405 nm. **D** Excitation at 458 nm. **E** Excitation at 488 nm. **F** Excitation at 514 nm.** G** Excitation at 561 nm.** H** Excitation at 633 nm.** I** EDX mapping of Na in tibia, tarsus 1, tarsus 2 and pretarsal claw in lateral view. The outline resembles the shape of the structure. **J** EDX mapping of Cl in tibia, tarsus 1, tarsus 2 and pretarsal claw.** K** EDX mapping of Ca in tibia, tarsus 1, tarsus 2 and pretarsal claw.** L** EDX mapping of Zn in tibia, tarsus 1, tarsus 2 and pretarsal claw. **cl** pretarsal claw. **fe** femur. **pfe** prefemur. **ta1** tarsus 1. **ta2** tarsus 2. **ti** tibia. **tr** trochanter

In the locomotory leg 10, sensilla trichodea are present on all podomeres, except the pretarsal claw, where sensilla coeloconica are present (Fig. 11A, A’). There is no autofluorescence gradient in the locomotory leg 10 (Fig. 11B); the leg emits weak but uniform autofluorescence throughout all podomeres when excited at 405 and 458 nm (Fig. 11C, D). Excitation at 488, 514 and 561 nm results in moderate autofluorescence in the podomeres (Fig. 11E–G). When excited at 633 nm, the podomeres emit very weak autofluorescence, but tracheal tubules in femur, tibia, tarsus 1, and tarsus 2 emit strong autofluorescence (Fig. 11B, H). The pretarsal claw does not emit autofluorescence when excited at any wavelength. Na, Cl and Ca are present in the locomotory leg 10, but without a concentration gradient (Fig. 11I–K). Zn is absent (Fig. 11L).

#### Cryptops hortensis (*Scolopendromorpha*)

**Fig. 12:**
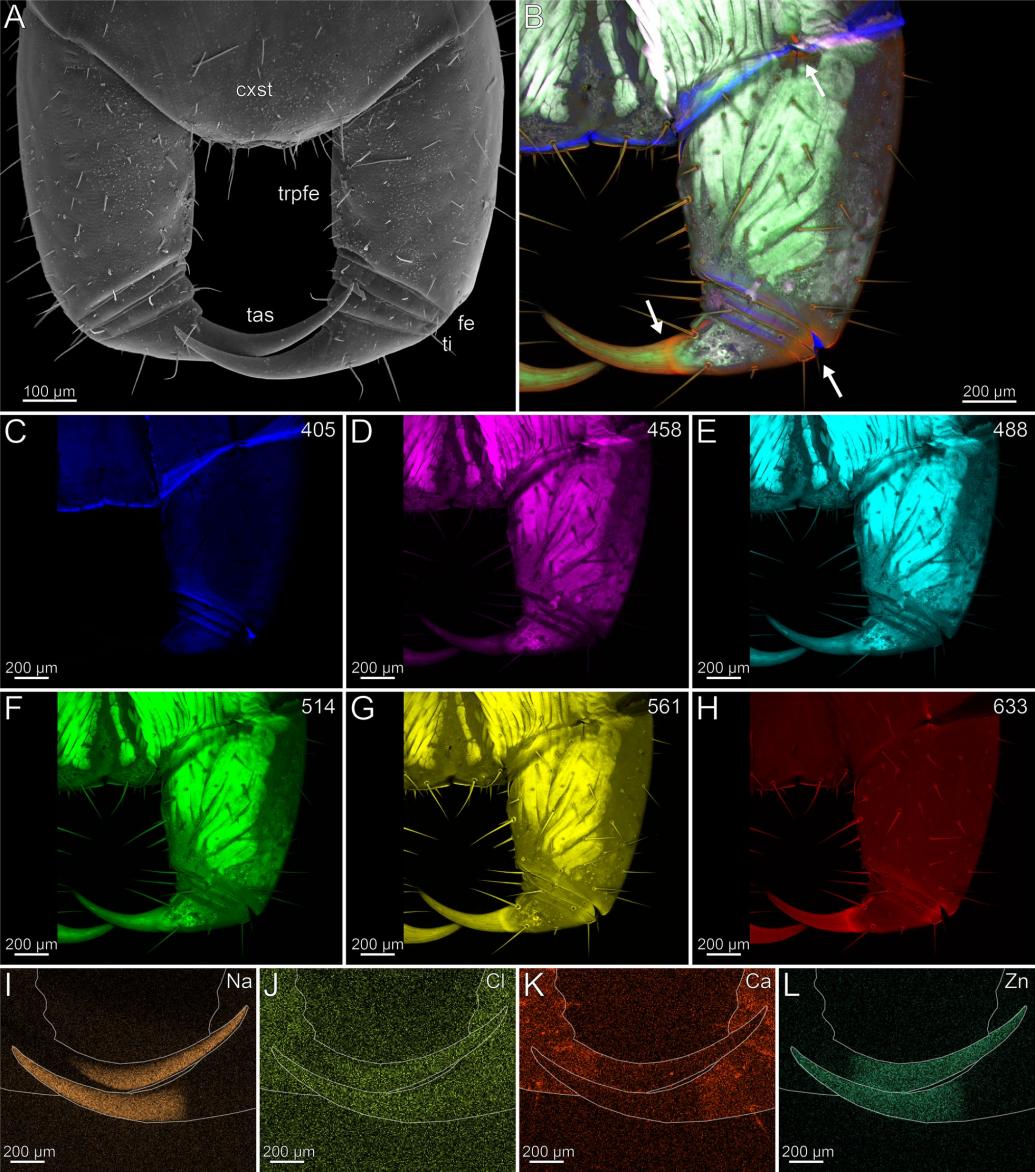
Forcipule of *Cryptops hortensis*. **A** Forcipule in ventral view (SEM). **B** Maximum intensity projection overlay (CLSM) excited at 405 nm, 458 nm, 514 nm and 633 nm. Ventral view. The arrows point at the joints and tarsungular suture. Sensilla coeloconica present. **C** Excitation at 405 nm. **D** Excitation at 458 nm. **E** Excitation at 488 nm. **F** Excitation at 514 nm.** G** Excitation at 561 nm.** H** Excitation at 633 nm.** I** EDX mapping of Na in the forcipule in ventral view. The outline resembles the shape of the structure. **J** EDX mapping of Cl in the forcipule.** K** EDX mapping of Ca in the forcipule.** L** EDX mapping of Zn in the forcipule. **cxst** coxosternite. **fe** femur. **tas** tarsungulum. **ti** tibia. **trpfe** trochanteroprefemur

The coxosternites are fused without a median suture (Fig. 12A). The proportions of the podomeres are approximately 10: 1: 1: 10 (trochanteroprefemur: femur: tibia: tarsungulum). The trochanteroprefemur and tarsungulum form a far lateral shared joint; the joint between the coxosternite and trochanteroprefemur is situated mediolaterally. The forcipule is sparsely covered with sensilla trichodea. Only at the distal tarsungulum sensilla coeloconica are present (Fig. 12A). The venom gland opening is located subterminally on the tarsungulum and opens dorsal. There is no distinct difference in radiodensity of the cuticle of trochanteroprefemur, femur, tibia and the proximal tarsungulum. The cuticle of the tarsungulum has a slightly higher radiodensity distally than proximally. The cuticle of the trochanteroprefemur (8.2 ± 0.9 µm) is thicker than the cuticle of femur (7.2 ± 0.4 µm) and tibia (7.5 ± 0.5 µm). The tarsungulum (10.2 ± 1.2 µm) exhibits the thickest cuticle. The cuticle at the tip of the tarsungulum (8.5 ± 1.5 µm) is slightly thinner.

**Fig. 13:**
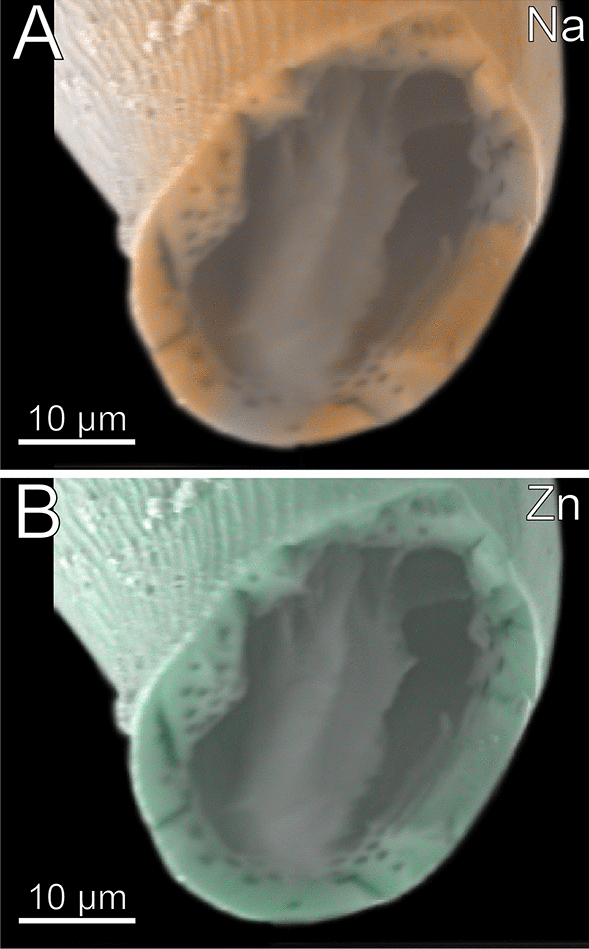
Cross-section of the tarsungulum of *Cryptops hortensis*. Frontal view. **A** EDX mapping of Na. **B** EDX mapping of Zn

The forcipule exhibits no autofluorescence gradient from coxosternite to tibia (Fig. 12B). However, the tarsungulum emits strong autofluorescence, especially at the suture at around one-third of the tarsungulum. When excited at 405 nm, coxosternite to tibia emit weak and uniform autofluorescence; the tarsungulum emits no autofluorescence (Fig. 12C). The interpodomeric membranes emit strong autofluorescence when excited at 405 nm. Excitation at 458, 488, 514 and 561 nm results in moderate autofluorescence in all podomeres (Fig. 12D–G). When excited at 458, 488, 514 and 561 nm, the musculature in the coxosternite, trochanteroprefemur as well as the venom gland in the tarsungulum emit moderate autofluorescence (Fig. 12D–G). When excited at 458, 488, 514, 561 and 633 nm, the sensilla and the pores of the sensilla sockets emit moderate autofluorescence. When excited at 633 nm, the joint between coxosternite and trochanteroprefemur, the shared joint of the tarsungulum and the trochanteroprefemur, and the distal part of the tarsungulum emit strong autofluorescence, which indicates that these regions are sclerotized (Fig. 12B, H). The tarsungulum exhibits a gradient of Na, Cl, and Zn towards its tip, starting at approximately one-thirds of its length with a distinct elemental border (Fig. 12I, J, L). At the proximal tarsungulum, Ca (also S and K, not shown) are present in slightly higher concentrations. Na, Cl, and Zn are absent (Fig. 12I–L). In the distal tarsungulum, Na, Cl and Zn are present in higher proportions (Fig. 12I, L). In cross-sections of the tarsungulum, Zn, Cl, and Na are present throughout the cuticle (Fig. 13A, B). The point measurements provide additional support for the presence of a graded distribution of Zn, but a gradient of Na and Cl is absent (Fig. 5C, D). Additionally, values of P + Pt are higher at the tip of the tarsungulum (Fig. 5D).

**Fig. 14:**
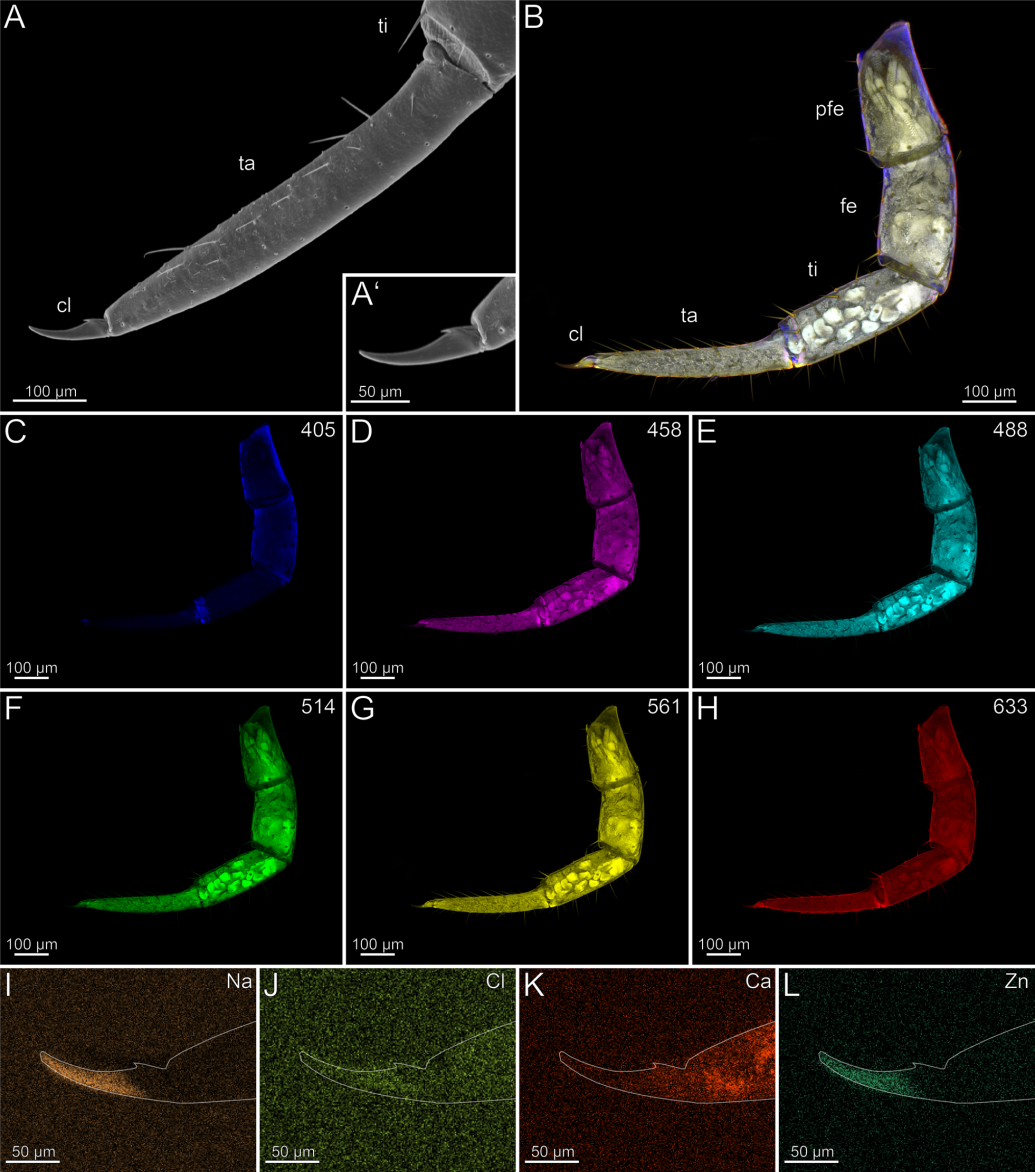
Locomotory leg 10 of *Cryptops hortensis.* **A** Locomotory leg 10 in lateral view (SEM). **A’** Pretarsal claw and tarsus. Lateral view. **B** Maximum intensity projection overlay (CLSM) excited at 405 nm, 458 nm, 488 nm, 514 nm, 561 nm and 633 nm. Lateral view. **C** Excitation at 405 nm. **D** Excitation at 458 nm. **E** Excitation at 488 nm. **F** Excitation at 514 nm.** G** Excitation at 561 nm.** H** Excitation at 633 nm.** I** EDX mapping of Na in tarsus and pretarsal claw in lateral view. The outline resembles the shape of the structure. **J** EDX mapping of Cl in tarsus and pretarsal claw.** K** EDX mapping of Ca in tarsus and pretarsal claw.** L** EDX mapping of Zn in tarsus and pretarsal claw. **cl** pretarsal claw. **fe** femur. **pfe** prefemur. **ta** tarsus. **ti** tibia. **tr** trochanter

In the locomotory leg 10, the tarsus possesses few sensilla trichodea, while sensilla coeloconica are only present on the pretarsal claw (Fig. 14A, A’). A sclerotization gradient is absent (Fig. 14B). When excited at 405 nm, prefemur, femur and the interpodomeric membranes between tibia and tarsus emit strong autofluorescence (Fig. 14C). Excitation at 458, 488, 514 and 561 nm results in autofluorescence of the musculature in prefemur, femur, and tibia (Fig. 14D–G). When excited at 514 and 561 nm, all podomeres emit moderate autofluorescence, while the pretarsal claw emits strong autofluorescence, which indicates that this region is sclerotized (Fig. 14F, G). When excited at 488, 514, 561 and 633 nm, sensilla and the pores of sensilla sockets emit moderate autofluorescence. When excited at 633 nm, the joints between tibia and tarsus, as well as tarsus and pretarsal claw emit strong autofluorescence, which indicates that this region is sclerotized (Fig. 14B, H). Na and Zn are present in higher concentrations in the distal pretarsal claw (Fig. 14I, L). Ca is present in the distal tarsus, but absent in the pretarsal claw (Fig. 14K). Cross-sections of the pretarsal claw reveals a high concentration of Zn and Na throughout the cuticle. An additional PDF file shows the EDX analysis in detail (Additional file 2).

#### Strigamia maritima (*Geophilomorpha*)

**Fig. 15:**
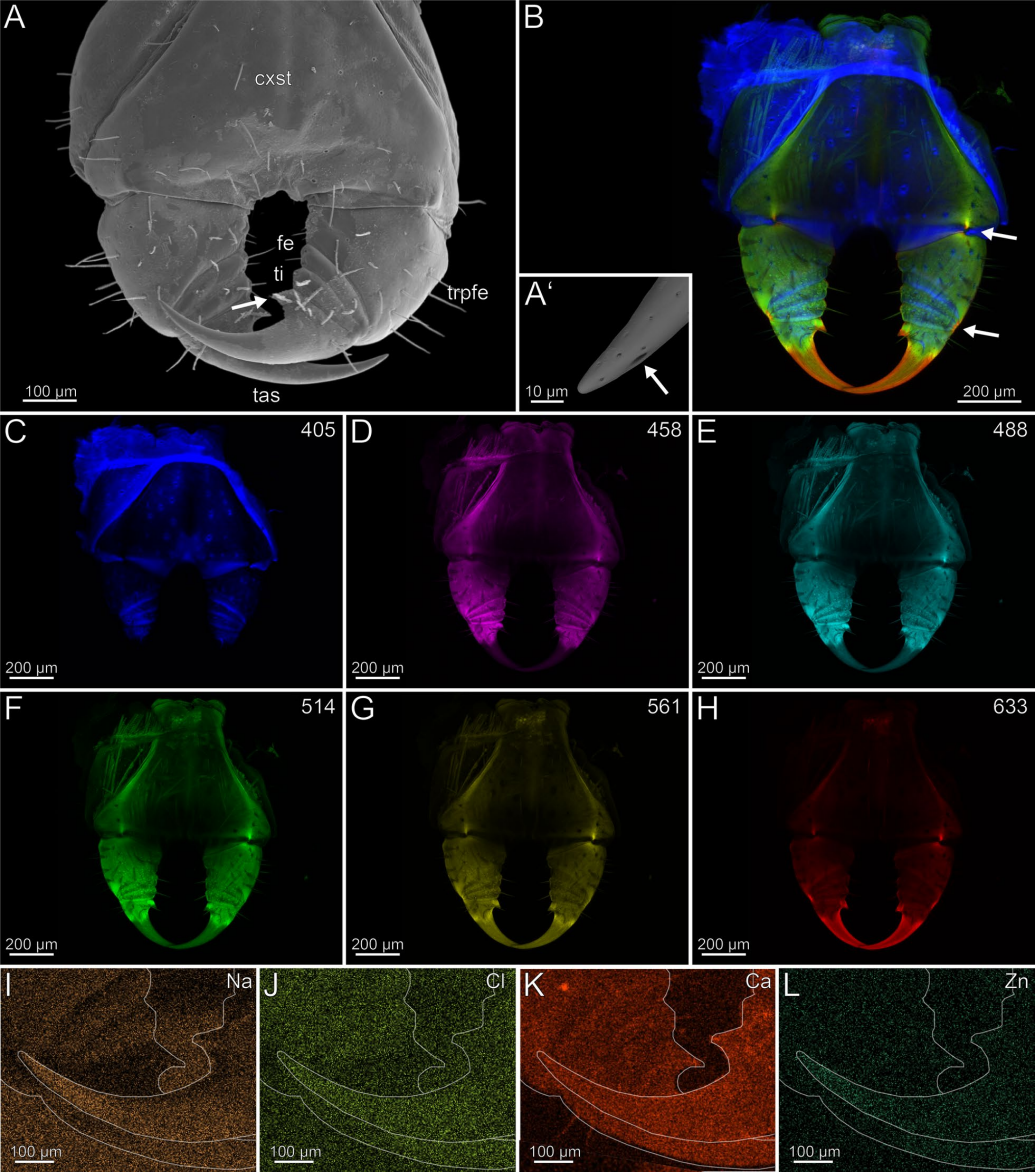
Forcipule of *Strigamia maritima.* **A** Forcipule in ventral view (SEM). The arrow points at the basal denticle of the tarsungulum. **A’** Tip of the tarsungulum. Dorsal view. Sensilla coeloconica present. The arrow points at the venom gland opening. **B** Maximum intensity projection overlay (CLSM) excited at 405 nm, 514 nm and 633 nm. Ventral view. The arrows point at the joints. **C** Excitation at 405 nm. **D** Excitation at 458 nm. **E** Excitation at 488 nm. **F** Excitation at 514 nm.** G** Excitation at 561 nm.** H** Excitation at 633 nm.** I** EDX mapping of Na in the forcipule in ventral view. The outline resembles the shape of the structure. **J** EDX mapping of Cl in the forcipule.** K** EDX mapping of Ca in the forcipule.** L** EDX mapping of Zn the forcipule. **cxst** coxosternite. **fe** femur. **tas** tarsungulum. **ti** tibia. **trpfe** trochanteroprefemur

The coxosternites of the forcipule are medially fused without a sutural ridge (Fig. 15A). The proportions of the podomeres are approximately 5: 1: 1: 6 (trochanteroprefemur: femur: tibia: tarsungulum). The trochanteroprefemur is as wide as it is long, femur and tibia are wider than long. The tarsungulum exhibits a basal denticle medially, and the venom gland opening is located subterminally opening dorsal (Fig. 15A’). The coxosternite and trochanteroprefemur possess a mediolateral joint, while the remaining podomeres form a shared lateral joint (Fig. 15A, B). The forcipule is sparsely covered with sensilla trichodea. Only at the distal tarsungulum, sensilla coeloconica are present (Fig. 15A, A’). The cuticle of the trochanteroprefemur (10.0 ± 2.0 µm) is thicker than the cuticle of the femur (7.7 ± 1.7 µm) and tibia (6.7 ± 1.1 µm), but not as thick as the cuticle of the tarsungulum (18.8 ± 6.9 µm). The tip of the tarsungulum (15.0 ± 3.3 µm) is slightly thinner. There is no distinct difference in radiodensity between the cuticle of the trochanteroprefemur and the proximal tarsungulum. The cuticle of the tip of the tarsungulum is less radiodense than its base.

The forcipule exhibits an autofluorescence gradient in the tarsungulum towards the tip (Fig. 15B). When excited at 405 nm, the cuticle emits weak autofluorescence, while the distal two-thirds of the tarsungulum emits no autofluorescence. The interpodomeric membranes and the membrane around the coxosternite emit strong autofluorescence, when excited at 405 nm, which indicates that these regions are not sclerotized (Fig. 15C). When excited at 458, 488 and 514 nm, the distal part of the trochanteroprefemur, femur, tibia, and the base of the tarsungulum emit moderate autofluorescence (Fig. 15D–F). When excited at 405, 458, 488, 514, 561 and 633 nm, sensilla and the pores of the sensilla sockets emit moderate autofluorescence. Excitation at 561 and 633 nm results in strong autofluorescence in the joint between coxosternite and trochanteroprefemur, the joint between trochanteroprefemur and tarsungulum, the tarsungulum, and the basal denticle of the tarsungulum (Fig. 15G, H). In the forcipule, Na and Zn are present at the distal two-thirds of the tarsungulum (Fig. 15I, L). Zn is only present in the distal tarsungulum, while Na is present in all podomeres, but in lower concentrations compared to the tarsungulum (Fig. 15I, L). Cl and Ca are present within the cuticle of all podomeres without any specific localisation (Fig. 15J, K). Cross-sections of the tarsungulum reveal a low concentration of Na and Cl, while Zn is absent. An additional image shows Na, Cl, Ca and Zn mapped onto the cross-section of the tarsungulum (Additional file 3).

**Fig. 16:**
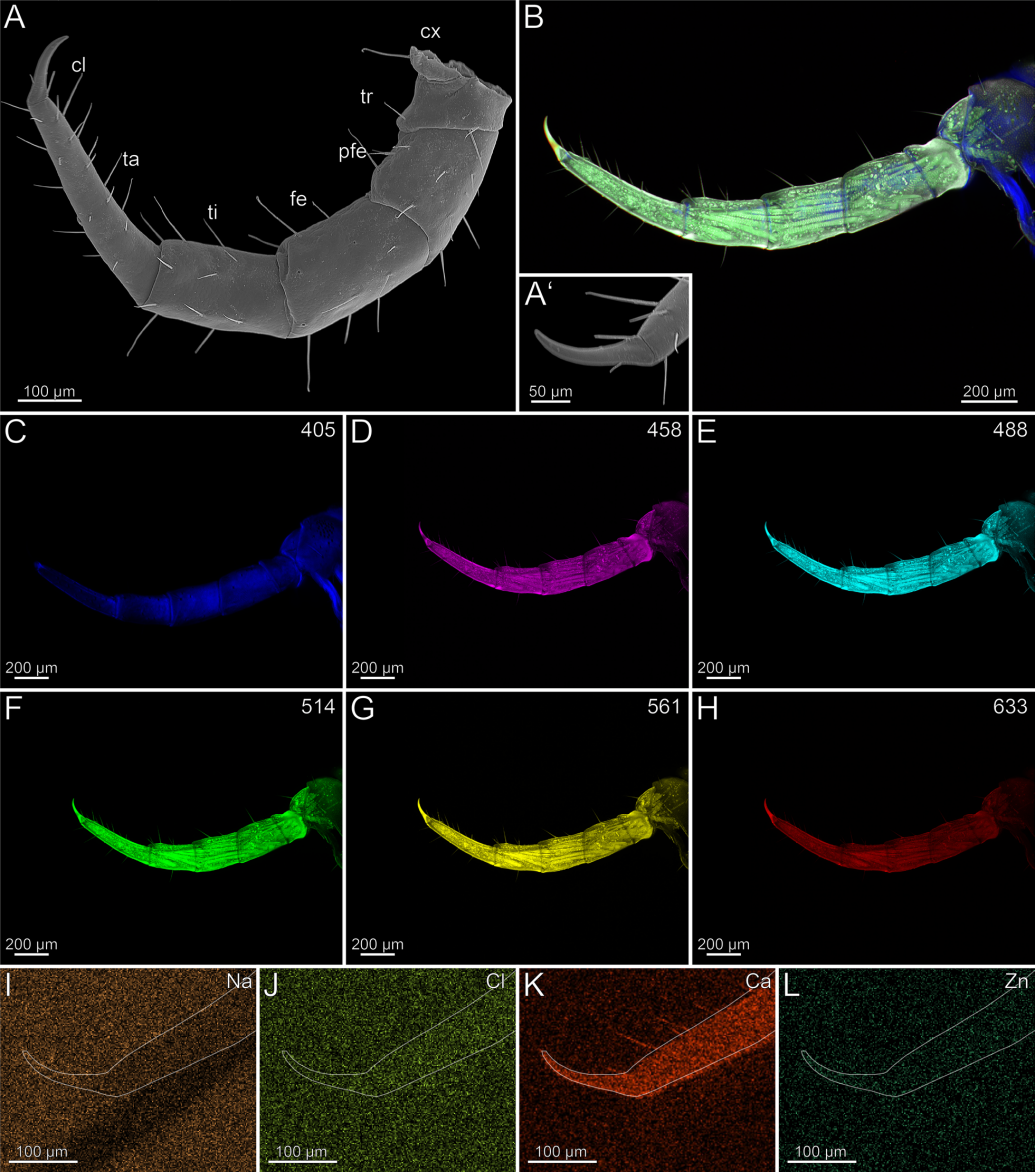
Locomotory leg 10 of *Strigamia maritima.* **A** Locomotory leg 10 in lateral view (SEM). **A’** Pretarsal claw and tarsus. Lateral view. **B** Maximum intensity projection overlay (CLSM) excited at 405 nm, 458 nm, 514 nm and 633 nm. Lateral view. **C** Excitation at 405 nm. **D** Excitation at 458 nm. **E** Excitation at 488 nm. **F** Excitation at 514 nm.** G** Excitation at 561 nm.** H** Excitation at 633 nm.** I** EDX mapping of Na in tarsus and pretarsal claw in ventrolateral view. The outline resembles the shape of the structure.** J** EDX mapping of Cl in tarsus and pretarsal claw.** K** EDX mapping of Ca in tarsus and pretarsal claw.** L** EDX mapping of Zn tarsus and pretarsal claw. **cl** pretarsal claw. **cx** coxa. **fe** femur. **pfe** prefemur. **ta** tarsus. **ti** tibia. **tr** trochanter

In the locomotory leg 10, sensilla trichodea are present on all podomeres, except the pretarsal claw, where sensilla coeloconica are present (Fig. 16A, A’, B). A pronounced autofluorescence and sclerotization gradient is absent (Fig. 16B). When excited at 405 nm, the leg emits weak autofluorescence, while the pretarsal claw emits no autofluorescence (Fig. 16C). Excitation at 458, 488, 514 and 561 nm results in moderate autofluorescence in all podomeres except the coxa (Fig. 16D–G). Additionally, the musculature, sensilla and the pores of the sensilla sockets emit moderate autofluorescence when excited at 458, 488, 514, 561 and 633 nm (Fig. 16D–H). When excited at 633 nm, the pretarsal claw emits strong autofluorescence, which indicates that this region is sclerotized (Fig. 16H). Na, Cl and Zn are absent in the locomotory leg (Fig. 16I, J, L). Ca is present throughout the cuticle, but without a concentration gradient or spots (Fig. 16K).

#### Haplophilus subterraneus (*Geophilomorpha*)

**Fig. 17:**
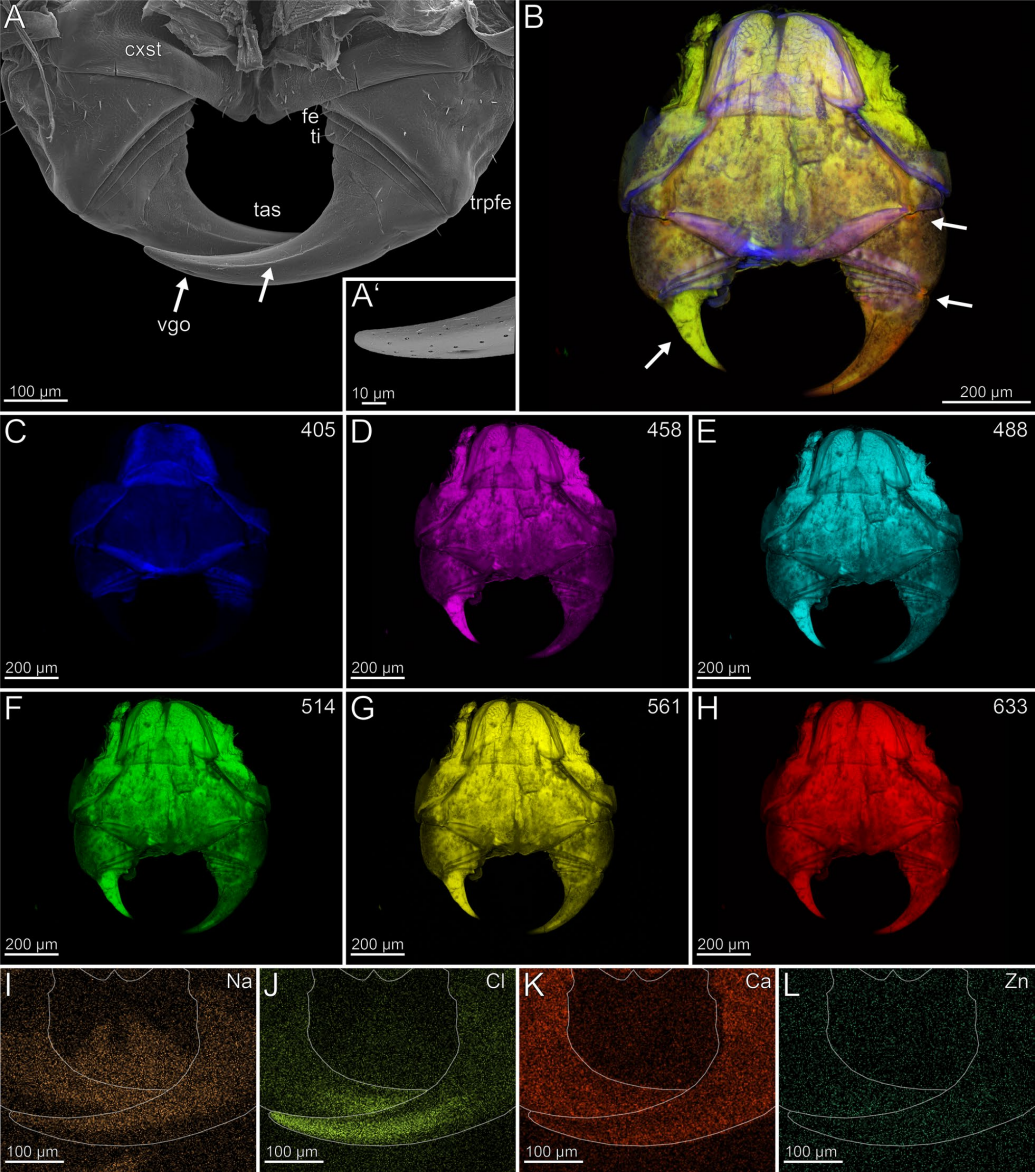
Forcipule of *Haplophilus subterraneus.* **A** Forcipule in dorsal view (SEM). The arrows point at the tarsungular dorsal ridge. **A’** Tip of the tarsungulum with venom gland opening. Dorsolateral view. Sensilla coeloconica present. **B** Maximum intensity projection overlay (CLSM) excited at 405 nm, 514 nm and 633 nm. Ventral view. The left arrow point to the regenerated tarsungulum, the right arrows to the joints. **C** Excitation at 405 nm. **D** Excitation at 458 nm. **E** Excitation at 488 nm. **F** Excitation at 514 nm.** G** Excitation at 561 nm.** H** Excitation at 633 nm.** I** EDX mapping of Na in the forcipule in ventral view (EDX). The outline resembles the shape of the structure. **J** EDX mapping of Cl in the forcipule.** K** EDX mapping of Ca in the forcipule.** L** EDX mapping of Zn in the forcipule. **cxst** coxosternite. **fe** femur. **tas** tarsungulum. **ti** tibia. **trpfe** trochanteroprefemur. **vgo** venom gland opening

The coxosternites of the forcipule are medially fused without a sutural ridge (Fig. 17A, B). The proportions of the podomeres are approximately 10: 1: 1: 20 (trochanteroprefemur: femur: tibia: tarsungulum). The trochanteroprefemur, femur, tibia, and tarsungulum form a shared lateral joint (Fig. 17A, B). The distal part of the tarsungulum features a dorsal ridge (Fig. 17A). The forcipule is sparsely covered with sensilla trichodea. Only at the distal tarsungulum, sensilla coeloconica are present. (Fig. 17A). The venom gland opening is located subterminally and opens dorsal (Fig. 17A’).

The forcipule exhibits a sclerotization gradient towards the tip of the intact (right) tarsungulum, while the regenerated (left) tarsungulum emits moderate autofluorescence (Fig. 17B). When excited at 405 nm, the cuticle emits weak autofluorescence. The tarsungulum emits almost no autofluorescence, while the interpodomeric membranes emit strong autofluorescence (Fig. 17C). When excited at 458, 488, 514 and 561 nm, the podomeres emit moderate autofluorescence (Fig. 17D–G). The left (regenerated) tarsungulum emits stronger autofluorescence compared to the right (intact) one (Fig. 17D–H). Excitation at 633 nm results in strong autofluorescence at the distal tarsungulum and the joints of the coxosternite and trochanteroprefemur, as well as the common joint of trochanteroprefemur and tarsungulum, which indicates that these regions are strongly sclerotized (Fig. 17H). In the tarsungulum, a Cl gradient is present in the distal two-thirds with an increasing concentration (Fig. 17J). Na and Ca are present throughout the cuticle without any concentration spot or gradient (Fig. 17I, K). Zn is absent (Fig. 17L). In cross-section of the tarsungulum, Cl is present throughout the cuticle. An additional image shows Cl mapped onto the cross-section of the tarsungulum (Additional file 4).

**Fig. 18:**
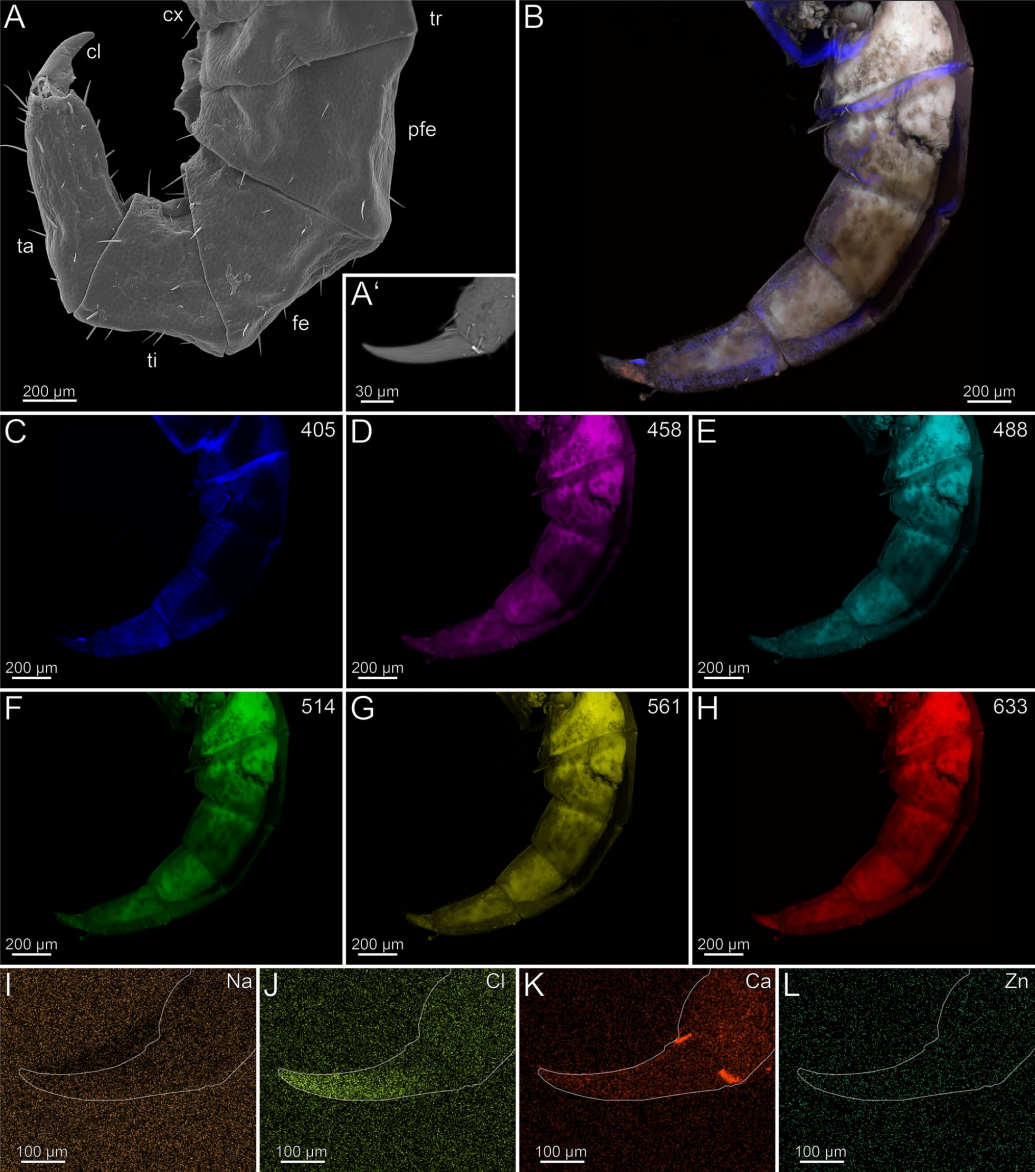
Locomotory leg 10 of *Haplophilus subterraneus.* **A** Locomotory leg 10 in lateral view (SEM). **A’** Pretarsal claw and tarsus. Lateral view. **B** Maximum intensity projection overlay (CLSM) excited at 405 nm, 458 nm, 514 nm, 561 nm and 633 nm. Lateral view. **C** Excitation at 405 nm. **D** Excitation at 458 nm. **E** Excitation at 488 nm. **F** Excitation at 514 nm. **G** Excitation at 561 nm.** H** Excitation at 633 nm.** I** EDX mapping of Na in tarsus and pretarsal claw in lateral view. The outline resembles the shape of the structure. **J** EDX mapping of Cl in tarsus and pretarsal claw.** K** EDX mapping of Ca in tarsus and pretarsal claw.** L** EDX mapping of Zn in tarsus and pretarsal claw. **cl** pretarsal claw. **cx** coxa. **fe** femur. **pfe** prefemur. **ta** tarsus. **ti** tibia. **tr** trochanter

In the locomotory leg 10, sensilla trichodea are present on all podomeres, except the pretarsal claw, where sensilla coeloconica are present (Fig. 18A, A’). There is no sclerotization gradient in the locomotory leg 10 (Fig. 18B). When excited at 405 nm, the interpodomeric membranes emit weak autofluorescence (Fig. 18C). When excited at 458, 488, 514, 561 and 633 nm, moderate autofluorescence is emitted in the cuticle of all podomeres, including the pretarsal claw (Fig. 18D–H). A Cl gradient is present in the pretarsal claw, with the concentration increasing distally (Fig. 18J). No other elemental gradients or increased concentrations were observed (Fig. 18I, K, L; the Ca spots in Fig. 18K are contaminations).

### Mechanical properties of selected centipede forcipules

We detected two different pathways in cuticular reinforcement, (a) without zinc in the tarsungulum in Scutigeromorpha and Lithobiomorpha, and (b) with zinc in the tarsungulum in Craterostigmomorpha and Epimorpha (Scolopendromorpha and Geophilomorpha). Therefore, we have chosen two representative species (*Lithobius forficatus* without zinc, and *Cryptops hortensis* with zinc) for further analyses gaining data on the mechanical parameters *E*, *H* and breaking forces and stresses. The Young’s modulus *E* describes the stiffness of a solid material, when it is subjected to stress (force per area). It correlates with the ability of the material to transmit force and relates to the puncturing function and material resistance to failure. Higher Young’s modulus values characterise a stiffer material, which means that it requires more force to achieve deformation or strain [53]. Hardness (*H*) describes the resistance to indentation and abrasion. Higher *H* values characterise a harder material, which means that it requires more force to achieve an indentation or wear.

**Fig. 19:**
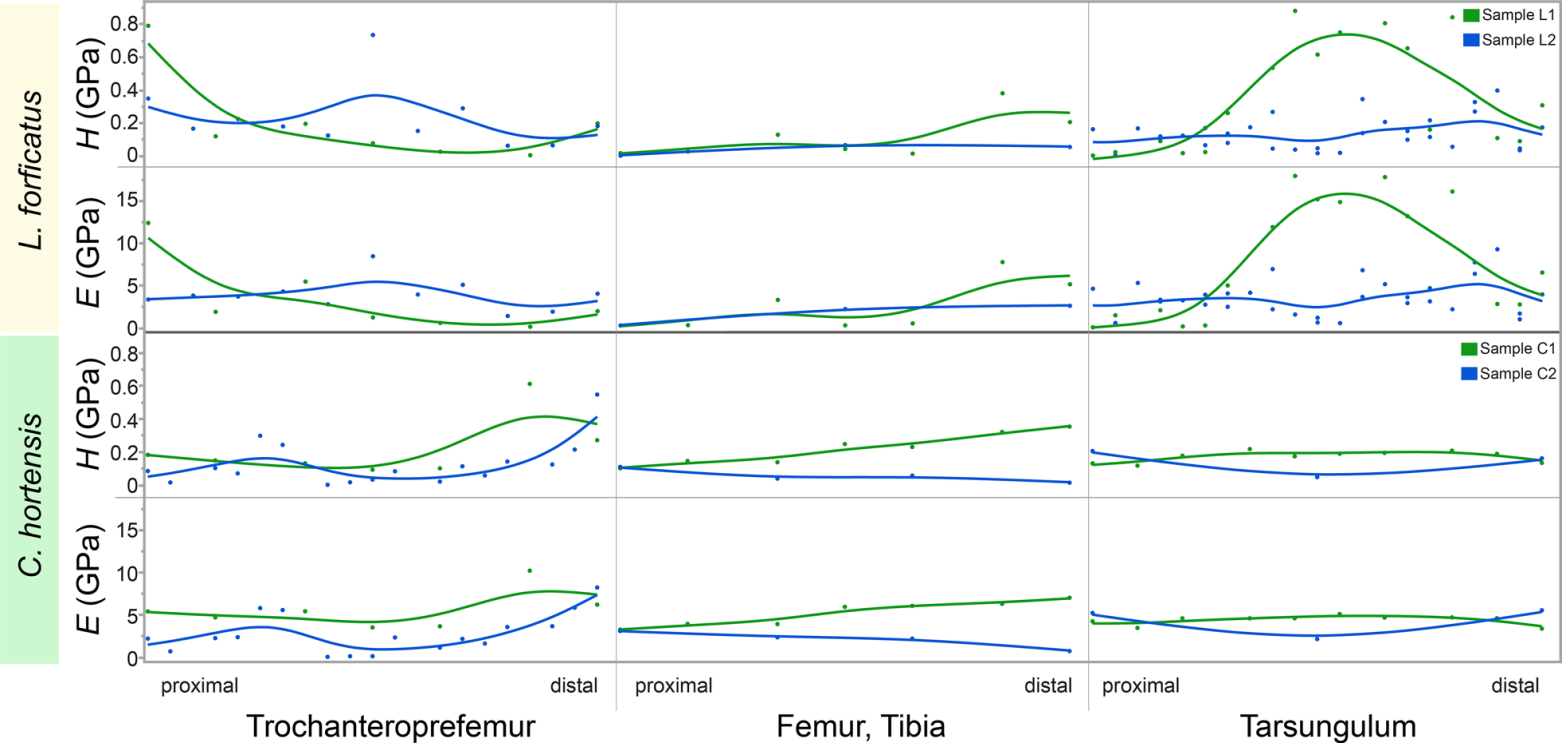
Hardness (*H*) and Young’s modulus (*E*), both given in GPa, of the forcipule of *Lithobius forficatus* (*n* = 2) and *Cryptops hortensis* (*n* = 2). *H* and *E* values are displayed in relation to the locality on trochanteroprefemur, femur and tibia, as well as tarsungulum. *H* and *E* correlate with each other in *L. forficatus* (Spearman’s rank correlation, correlation coefficient: 0.916, *p* < 0.001) and *C. hortensis* (Spearman’s rank correlation, correlation coefficient: 0.949, *p* < 0.001). *H* of the tarsungulum of *L. forficatus* is statistically not different to the *H* of other podomeres (Mann–Whitney rank sum test,* U* = 683, *T* = 1095, *p* = 0.979) and *E* of the tarsungulum is statistically not different to the *E* of other podomeres (Mann–Whitney rank sum test,* U* = 538, *T* = 944, *p* = 0.118). *H* of the tarsungulum of *C. hortensis* is statistically not different than the *H* of other podomeres (Mann–Whitney rank sum test,* U* = 168, *T* = 378, *p* = 0.171) and *E* of the tarsungulum is statistically not different than the *E* of the podomeres (Mann–Whitney rank sum test,* U* = 190, *T* = 356, *p* = 0.391). The tarsungula of *L. forficatus* and *C. hortensis* are statistically not different in hardness *H* (Mann–Whitney rank sum test,* U* = 260, *T* = 468, *p* = 0.316) and elastic modulus *E* (Mann–Whitney rank sum test,* U* = 257, *T* = 471, *p* = 0.292)

*H* and *E* correlate with each other in *L. forficatus* (Spearman’s rank correlation, correlation coefficient: 0.916, *p* < 0.001) (Fig. 19). In the forcipule of *L. forficatus*, *H* values range from 0.01 to 0.88 GPa (mean ± standard deviation: 0.21 ± 0.22 GPa) and *E* values range from 0.11 to 17.99 GPa (4.39 ± 4.25 GPa). *H* of the tarsungulum (0.25 ± 0.26 GPa) is statistically not different to the *H* of other podomeres (0.16 ± 0.17 GPa) (Mann–Whitney rank sum test, *U* = 683, *T* = 1095, *p* = 0.979). *E* of the tarsungulum (3.69 ± 5.97 GPa) is statistically not different to the *E* of other podomeres (1.15 ± 2.50 GPa) (Mann–Whitney rank sum test, *U* = 538, *T* = 944, *p* = 0.118). The values of *H* and *E* do not increase towards tip of the tarsungulum, but in one of the measured specimens (sample L2) increased *H* and *E* values are present in the distal one-third of the tarsungulum.

In the forcipule of *Cryptops hortensis*, *H* values range from 0.003 GPa to 0.61 GPa (0.16 ± 0.12 GPa) and *E* values range from 0.07 to 10.25 GPa (3.95 ± 2.13 GPa). *H* of the tarsungulum (0.17 ± 0.05 GPa) is statistically not different than the *H* of other podomeres (0.16 ± 0.14 GPa) (Mann–Whitney rank sum test, *U* = 168, *T* = 378, *p* = 0.171). *E* of the tarsungulum (4.40 ± 0.90 GPa) is statistically not different than the *E* of the podomeres (3.79 ± 2.42 GPa) (Mann–Whitney rank sum test, *U* = 190, *T* = 356, *p* = 0.391). *H* and *E* correlate with each other in *C. hortensis* (Spearman’s rank correlation, correlation coefficient: 0.949, *p* < 0.001). Thus, the tarsungula of *L. forficatus* and *C. hortensis* are statistically not different in hardness *H* (Mann–Whitney rank sum test, *U* = 260, *T* = 468, *p* = 0.316) and elastic modulus *E* (Mann–Whitney rank sum test, *U* = 257, *T* = 471, *p* = 0.292). All measured *H* and *E* means are listened in Additional file 5

### Resistance to structural failure

**Fig. 20:**
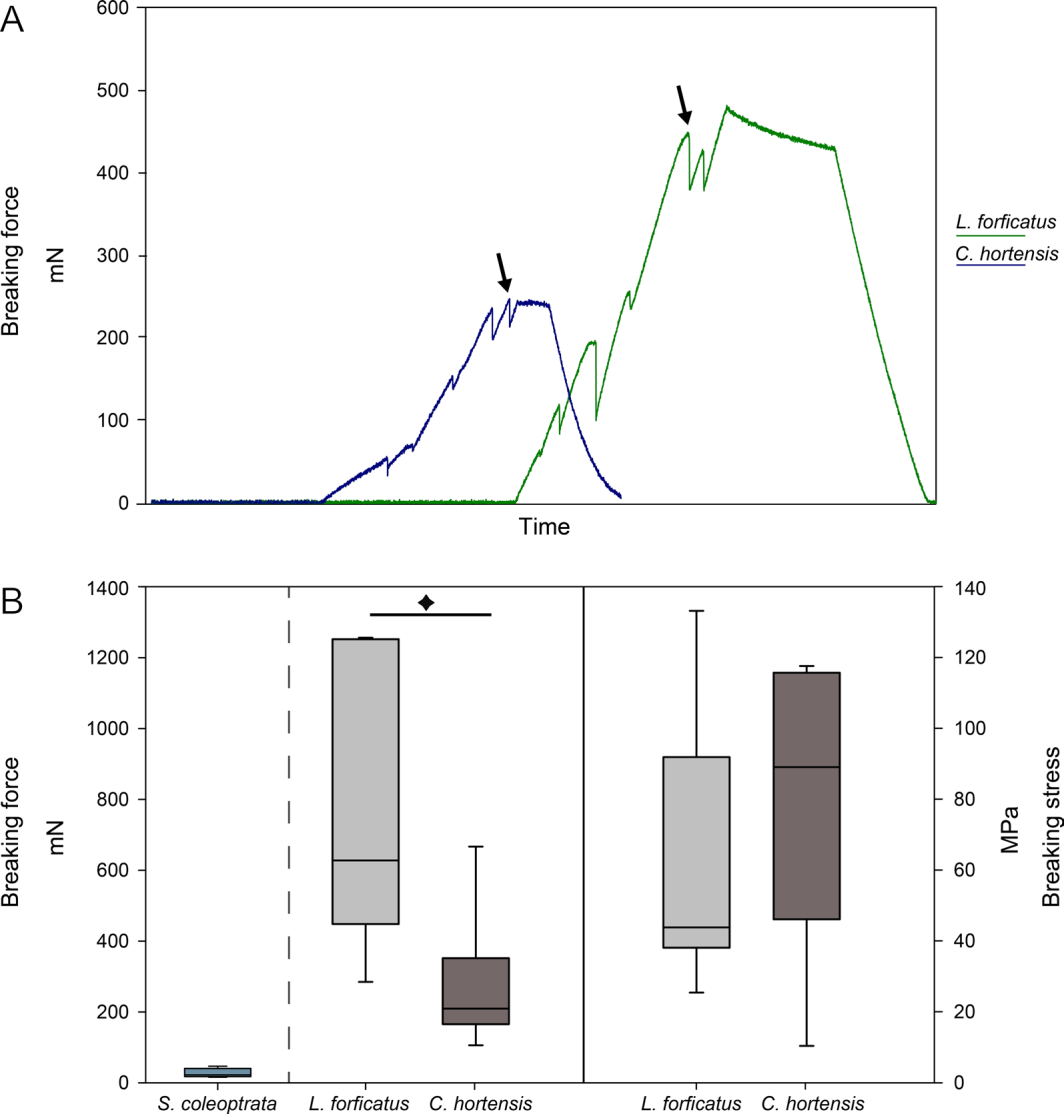
Results from breaking force measurements of forcipules of *Scutigera coleoptrata, Lithobius forficatus* and *Cryptops hortensis*. **A** Exemplary force–time-curves for breaking force measurements of *L. forficatus* (green) and *C. hortensis* (blue). Arrows point to the moment of fracture of the tarsungulum. **B** Left: Breaking forces, given in mN, in *S. coleoptrata* (*n* = 5 tarsungula), *L. forficatus* (*n* = 7 tarsungula) and *Cryptops hortensis* (*n* = 6 tarsungula). Differences between the forces in *L. forficatus* and *C. hortensis* are statistically significant (t-test, t = 2.879, *p* = 0.015, marked with a star). Right: Breaking stress, given in MPa. Data presented as box-whisker-plots. Boxes indicate 25th and 75th percentiles and whiskers indicate 10th and 90th percentiles. The line represents the median

To compare the biological relevance of different incorporated elements in the tarsungular cuticle, the compressive force required to break the structure was measured and the corresponding stress was determined. The tarsungula of *L. forficatus* and *C. hortensis* broke with a linear fracture at approximately one-third of their length, aligning with the location of the incomplete suture of the tarsungulum (Fig. 1E, F). However, when applying force to the tip of the tarsungulum of *S. coleoptrata*, their forcipules did not break at all, but underwent plastic deformation without leaving a clear breakage point (Fig. 1D). This deformation occured instantaneously upon force application and no cross-sectional area was measurable. The tarsungula of *L. forficatus* and *C. hortensis* both did not strongly deform while force was applied, but instead instantaneously broke at a certain load (Fig. 20A).

The mean lengths of the assessed forcipules are 4 mm in *Lithobius forficatus*, approximately 2 mm in *Cryptops hortensis*, and 3 mm in *Scutigera coleoptrata*. The cuticle thicknesses of the tarsungula are: 14.8 ± 1.6 µm (mean ± standard deviation), with 10.0 ± 2.8 µm at the tip (*S. coleoptrata*); 32.2 ± 3.8 µm, with 33.6 ± 9.1 µm at the tip (*L. forficatus*); and 10.2 ± 1.2 µm, with 8.5 ± 1.5 µm at the tip (*C. hortensis*). The tarsungula of *L. forficatus* endured significantly higher loading forces (t-test, *t* = 2.879, df = 11, *N*_L. forficatus_ = 7, *N*_c. hortensis_ = 6, *p*_*two-tailed*_ = 0.015) with 781.09 ± 390.91 mN compared to *C. hortensis* with 270.77 ± 199.81 mN (Fig. 20B). The force measured during loading of the tarsungulum of *S. coleoptrata* (*N* = 5) was 33.96 ± 13.64 mN resulting only in deformation without any fracture. The cross-sectional areas at the point of breakage in *L. forficatus* was 14,797.9 ± 8,672.5 µm^2^ and in *C. hortensis* 4,357.6 ± 3,152.8 µm^2^. The respective cross-sectional areas were used for the calculations of the breaking stress.

While the resulting breaking stress of the forcipules of *L. forficatus* was 62 ± 38 MPa, breaking stress in *C. hortensis* was 79 ± 41 MPa. The breaking stresses did not differ significantly between *L. forficatus* and *C. hortensis* (t-test, *t* = -0.799, df = 11, *N*_L. forficatus_ = 7, *N*_C. hortensis_ = 6, *p*_*two-tailed*_ = 0.441) (Fig. 20B). All measured forces, cross-sectional areas and resulting stresses are listed in Additional file 6.

## Discussion

### Transformations and sclerotization in the centipede forcipule and locomotory leg

**Fig. 21:**
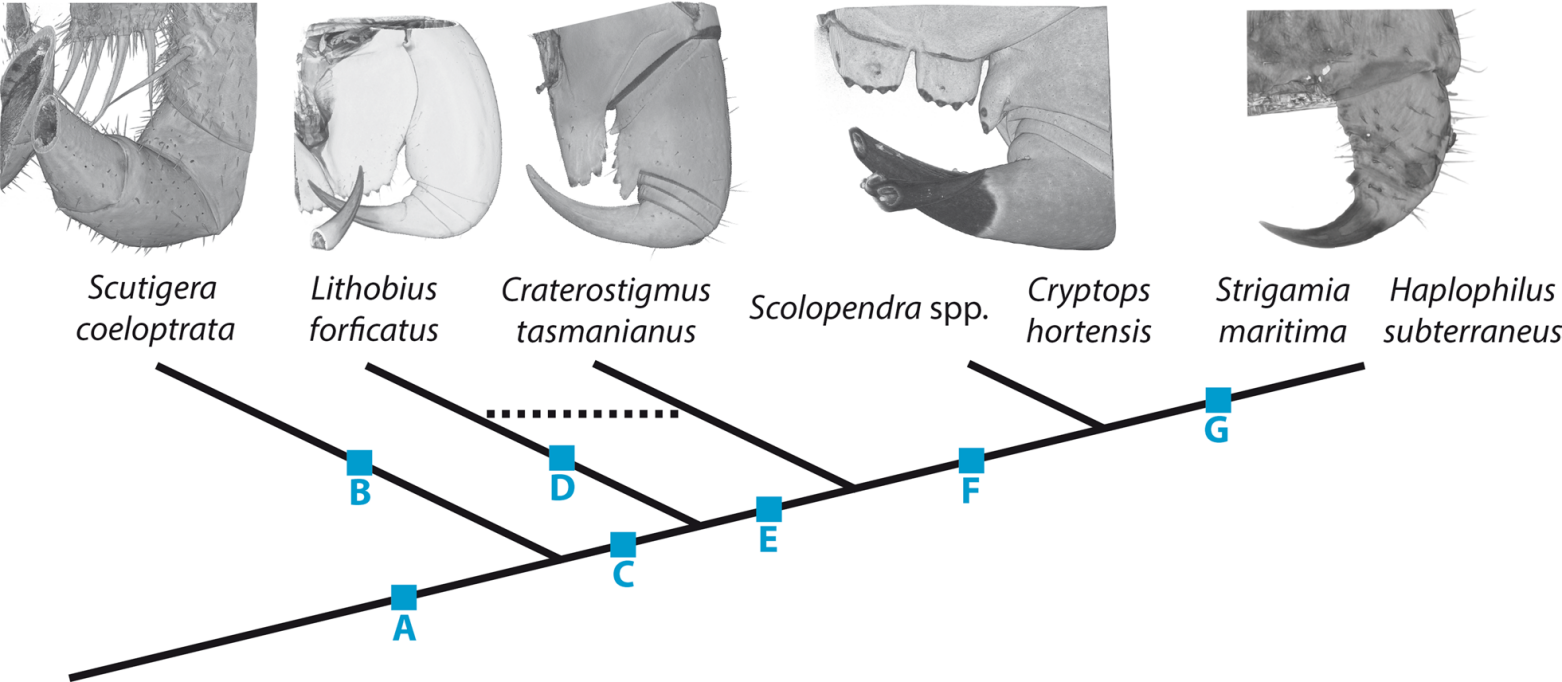
Evolutionary scenario of morphological characters of the centipede forcipule (including characters listed by Edgecombe [55] and Haug et al. [28]). Note that the phylogenetic positions of Lithobiomorpha and Craterostigmomorpha are ambiguous (compare Benavides et al. [58]). **A (Chilopoda)** First trunk segment appendages transformed into forcipules housing venom glands. Three specific types of coeloconica sensilla. Complete tarsungular suture present. Ca and Cl present in the cuticle of the tarsungulum (without any gradient). Moderate sclerotized tarsungulum. Venom gland opening located subterminally and opening dorsally. Joints of telopodite positioned medially. **B (*Scutigera coleoptrata*):** Coxosternite medially unfused (potentially plesiomorphic). Coxosternite with four elongate spine-bristles and a corresponding spine-bristle on the forcipular trochanteroprefemur (see Fig. 2B). **C (Pleurostigmophora):** Coxosternite medially fused featuring a suture. Significantly shorter forcipular femur and tibia. Coxal apodemes on the forcipules. Incomplete tarsungular suture. Strongly sclerotized tarsungulum and joints. Joints of telopodite positioned laterally. **D (*Lithobius forficatus*):** Porodont on the lateral part of the forcipular dental margin (see Fig. 4B). Ca as tarsungular gradient. Two differently radiodense layers at the tarsungular tip. **E (Phylactometria):** Rigid forcipules (pleurite arching over the coxosternite, sclerotized hinge joint between coxosternite and trunk). Na and Zn present in the tarsungular cuticle. **F (Epimorpha)**: Coxosternite medially fused without visible suture. Shared lateral joints from trochanteroprefemur to tarsungulum. Zn present in the tarsungulum featuring a gradient. **G (Geophilomorpha)**: Zn (potentially plesiomorphic) or Cl present in the tarsungulum featuring a gradient

As demonstrated by Haug et al. (2014), taxon-specific characteristics in forcipule morphology are evident in centipedes (Fig. 21). In Scutigeromorpha, the forcipule takes up a leg-like appearance, while in other centipede taxa, it has undergone pronounced adaptations evolving into a rigid piercing structure. Unlike Scutigeromorpha, where the coxosternites are medially separated, Pleurostigmophora exhibit medially fused coxosternites. Furthermore, the coalescent coxosternite displays a longitudinal median sutural ridge in Lithobiomorpha and Craterostigmomorpha, a character that is absent in Epimorpha (Fig. 21). Additionally, in the course of evolution, modifications in length and size have occurred in the podomeres of the forcipular telopodite. Unlike Scutigeromorpha and Lithobiomorpha, where the femur and tibia are only moderately shorter than the trochanteroprefemur and the tarsungulum, species within Craterostigmomorpha and Epimorpha exhibit highly condensed femora and tibiae [28]. Notably, Craterostigmomorpha displays a mixture of lithobiomorph and scolopendromorph characteristics [54] (Fig. 21). This is evident in the fusion of the coxosternite with a subtle median sutural ridge, the formation of complete podomere rings with separate joints of femur and tibia (like in Lithobiomorpha), and the presence of two median serrated projections on the coxosternite (like in many Scolopendromorpha), thus confirming Dohle's [54] earlier observations. This ambiguity also reflects the conflict in the phylogenetic position of Craterostigmomorpha based on morphological and molecular analyses [34, 55–58]. Epimorpha are further characterised by a combined lateral hinge joint between trochanteroprefemur and tarsungulum. While in Scutigeromorpha, a tarsungular suture is present at around two-third of the tarsungulum, it is externally not clearly visible in Pleurostigmophora (see also below). In all examined species, the venom gland opening is located subterminal and always opens dorsal.

Autofluorescence experiments showed that in Scutigeromorpha, a subtle gradient of sclerotization towards the tarsungulum is evident in the forcipular elements, while moderate sclerotization is present at the joints between the podomeres. In contrast, in Pleurostigmophora a progressive increase in sclerotization is evident, with moderate sclerotization (excitations at 488, 514 and 561 nm) from trochanteroprefemur to tibia and stronger sclerotization in the tarsungulum (mostly excited at 633 nm). The sclerotization of the lateral joints of the telopodite intensifies to a higher degree in *C. tasmanianus*, and reaches even greater levels in the common telopodal joints of Scolopendromorpha and Geophilomorpha. In the case of *C. tasmanianus* and *S. oraniensis*, only minimal or faint autofluorescence in the tarsungulum was observed, which possibly is associated with their very thick tarsungular cuticle (~ 45 µm in *C. tasmanianus*, ~ 60 µm in *S. morsitans*). Although microCT analysis was performed in a different species of *Scolopendra*, we assume that material properties of the tarsungula in these closely related species is comparable. The lack of autofluorescence, however, indicates the limitations of this method in visualising thicker and more strongly sclerotized cuticular structures.

Except for *S. coleoptrata*, in all other examined species the cuticle's sclerotization gradient in the locomotory leg 10 is comparatively lower than that of the forcipule in the same species. The relatively weak sclerotized tarsus of *S. coleoptrata* is likely attributed to the length of the legs and to the transformation of the flexible tarsi, which enable a plantigrade stance and serve in entangling prey [29, 59]. Notably, the pretarsal claw of the locomotory leg in Pleurostigmophora possesses the strongest sclerotization, while the distal podomere is slightly stronger sclerotized than proximally. The stronger sclerotization present in the pretarsal claw of the locomotory leg, the distal section of the forcipular tarsungulum, and the lateral joints of the forcipular telopodite is plausibly linked to heightened interaction and exposure to the external environment. The leg’s pretarsal claw is in direct contact with the substrate during locomotion and thus prone to increased wear and tear [60]. The forcipule, especially the tip of the tarsungulum, but also the joints of the forcipular telopodite, likewise have to endure substantial stress during prey capture and skin penetration, and inherently necessitate robust structural support [7, 53].

### Material composition and its influence on the mechanical behaviour of structures

The cuticle thickness of the tarsungulum differs in different centipedes. In Lithobiomorpha, there are no differences between the tip and base, while in Scutigeromorpha and Phylactometria, the cuticle gets thinner towards the tip. With the exception of the tarsungulum, the radiodensity across the forcipule was uniform in all examined species. In Scutigeromorpha, the radiodensity was consistent across the forcipule. In Craterostigmomorpha, Scolopendromorpha and Geophilomorpha the tip was less radiodense compared to the base of the tarsungulum, while in *C. hortensis* the tip was more radiodense. The cuticle of the tip of the tarsungulum of *L. forficatus* is double-layered, with a dense inner layer and a distinct border to a less dense outer layer. The higher radiodensity in the tip of the tarsungulum in *C. hortensis* is unlikely influenced by the Zn concentration, as compared to other species with Zn incorporation (*C. tasmanianus, S. maritima*), where the radiodensity was not increased. It is possible, that the orientation, fine structure and density of microfibrils, and stronger sclerotization of the cuticle lead to a higher radiodensity. This is also likely to be the cause of the more radiodense inner layer of the cuticle of the tarsungulum of *L. forficatus*.

In Scolopendromorpha and Geophilomorpha, the concurrent presence of Zn and Cl within the cuticle of the tarsungulum and the pretarsal claw of the locomotory leg suggests a functional role akin to that observed in structures with high mechanical demand of other arthropods, including spider chelicerae, scorpion stinger and chelicerae, ant mandibles and mole cricket claws [1, 10, 44, 61]. Transition metals, such as Zn, exhibit the capacity to increase hardness, stiffness, and abrasion resistance of the arthropod cuticle, rendering it less susceptible to blunting and diminishing the force, energy, and muscle effort required to puncture rigid objects [5]. This augmented mechanical performance proves especially advantageous for smaller organisms that necessitate the penetration, cutting, and grasping of objects that would otherwise remain inaccessible in the absence of elemental enrichment. It is important to note that even larger animals can derive the energy-saving benefits stemming from reduced force demands [5]. Previous research has indicated a notable two- to threefold enhancement in hardness within Zn enriched cuticles, signifying that Zn integration amplifies the mechanical attributes of the cuticle [1]. The presence of Zn exclusively along the cutting edges of the tarsungula in *C. tasmanianus* and spanning the distal segment of the tarsungulum in *C. hortensis* and *S. maritima* corresponds with the pattern observed in other arthropod piercing structures, where element enrichment of the cuticle tends to concentrate in the piercing regions [12]. The coexistence of Zn and Cl in *S. maritima*, in contrast to the exclusive presence of Cl in *H. subterraneus*, suggests that halogens without the addition of Zn may not correlate with higher values of *E* and *H* [62]. This implies that the combination of Zn and Cl might be pivotal for achieving the sought-after mechanical enhancements within the cuticle of the forcipule.

Maintaining the physical integrity of their primary prey-capturing organ is of paramount importance for centipedes, which is additionally aided by their regular molts and the ability to regenerate legs and forcipules [63]. Progressively regenerated forcipules may exhibit smaller dimensions, incomplete podomeres, fewer sensilla, and potentially lack a venom gland [64]. The characteristics of the partially regenerated forcipule in *H. subterraneus* (Fig. 17B) indicates that in contrast to previous reports [65], appendage regeneration is also present in Geophilomorpha.

Among the studied species of Scutigeromorpha, Lithobiomorpha, and Scolopendridae, the absence of elemental enrichment with metals in the forcipule implies that they likely evolved different strategies for cuticle reinforcement. The tarsungula of *S. oraniensis* and *L. forficatus* display a darkened cuticle, which is often associated with tanning/ sclerotization (Fig. 1A) [62]. Sclerotization is the process that stabilises proteinaceous structures in arthropods through quinone-protein reactions, leading to progressive dehydration, compact arrangement, and reinforcement of polymer composites [66]. This could potentially constitute as a strengthening strategy for the tarsungulum. Furthermore, mechanical attributes of the cuticle can be significantly influenced by the secondary and tertiary structure of matrix proteins, along with the cross-linking of this matrix through sclerotization [17]. The interplay between proteins and chitin, coupled with metal-free tanning processes, might strenghten the centipede cuticle without the incorporation of minerals [17]. However, in certain instances, hardening and sclerotization mechanisms can even surpass the mechanical properties achieved through the incorporation of metals [62]. For example, mandibles of beetle larvae (*Pseudotaenia frenchi* (Blackburn, 1891)) lack a transition metal enriched cuticle, but exhibit higher *H* and *E* values in contrast to those of their adults that possess manganese incorporation [62].

In the instance of Scutigeromorpha, the tarsungulum has a circular cross-section and lacks a distinct cutting edge, exhibiting comparably lower sclerotization in comparison to other taxa. This observation suggests that Scutigeromorpha may have adopted an alternative or maintained a plesiomorphic strategy to attain a robust and enduring forcipule. It is plausible that factors beyond hardness and stiffness, which are characteristic of transition metal enriched cuticle, contribute to the reinforcement of the forcipule [62]. While Ca is present in the tarsungula of all examined species*,* the presence of a Ca gradient within the tarsungulum of Lithobiomorpha is intriguing. The reinforcement of cuticle with Ca compounds is a widely known mechanism in crustaceans and some insects, associated with increased hardness through the deposition of calcium salts, such as calcium phosphate and calcium carbonate [67, 68]. As the detected P did not correlate with the presence of Ca, it is unlikely that Ca is deposited as a phosphorus salt, but rather is cross-linked with the cuticle matrix, as also suggested in ovipositors of wasps [69], or deposited as calcium carbonate or calcite, as found in larvae of the black soldier fly [68]. However, a more detailed investigation is required to unravel mechanisms of calcium incorporation and its role in strengthening the cuticle.

### Mechanical properties of calcium and zinc enriched forcipules

In the experimental evaluation of the diverging strategies in cuticular composition in *L. forficatus* (Ca exhibiting a tarsungular gradient) and *C. hortensis* (Zn exhibiting a tarsungular gradient), no significant differences in breaking stress, the Young’s modulus and hardness were detected. The measured breaking force in the two species was significantly different and the force needed to break a tarsungulum of *L. forficatus* was almost three times higher than the force needed to break a tarsungulum of *C. hortensis*. However, correcting for the tarsungular cuticle thickness in *L. forficatus*, the stress that it can withstand is not different in both species. This is because the ratio of radius to cuticle thickness of the load bearing structure influences the capability of plastic and elastic deformation, and determines the failure resistance [70]. The breaking experiments revealed that neither Ca nor Zn influenced the strength of the tarsungula. In contrast, the forcipule of *S. coleoptrata* did not break and the force measured until deformation were eight times lower than those observed in *C. hortensis*, even though the forcipule is large. Notably, the forcipule of *S. coleoptrata* is less sclerotized, the podomeres are rather long, the tarsungulum has a circular cross-section and there is a larger freedom of movement [28]. Although the tarsungular cuticle of *L. forficatus* only exhibits Ca as a gradient, the forces endured (781.09 ± 390.91 mN) are similar to the forces endured by e.g., the jaws of an antlion larva (700.00 ± 195.92 mN), which exhibits Ca, Mn, Fe, Cu and Zn in its piercing mouthparts [26].

Surprisingly, hardness and Young’s modulus of the cuticle did not increase towards the tip of the tarsungulum, although Ca or Zn was present in a higher concentration at the tarsungulum tip. This is in contrast to findings in other cutting and piercing tools of arthropods [5, 13, 26, 71]. In many arthropod mandibles, the occurrence of Ca and/or Zn have been proven to increase the cuticles *H* and *E* and therefore improve both wear and failure resistance [1, 5, 12, 26, 61, 72]. Additionally, these increased material properties along the masticatory margin usually correlate with an increased concentration of transition metals such as Zn [5], which is not the case in the centipede forcipule. In comparison to other arthropods, the *H* values of the centipede’s forcipule (0.16–0.21 GPa) are within average of the piercing mouthparts of cicadas (*H*: 0.16 GPa) [71], the tip of a spider chelicerae (*H* from base to tip: 0.6–0.16 GPa) [7], or the scorpion chela (*H*: 0.9–2.3 GPa) [73]. However, *E* values in centipedes (3.95–4.39 GPa) are higher than that of cicada mandibles (*E*: 2.16 GPa) [71] or the scorpion stinger (*E*: 0.8–1.8 GPa) [74], in the range of the scorpion chela (*E*: 3.3–9.5 GPa) [73], but lower than of spider chelicerae (*E* from base to tip: 13–20 GPa) [7], or the antlion larva jaw (*E*: 3.47–20.88 GPa) [26].

The centipede tarsungulum, the spider chelicerae and the scorpion stinger all decrease in their cross-sectional profile from proximal to distal. Although similar in their structural gradient, *H* and *E* values of the tarsungula decrease towards the tip, while *H* and *E* increase in the chelicerae and stinger [7, 74]. The increase in both values (*H* and *E*) in one specimen of *L. forficatus* is presumably an artefact due to the indentation around the strongly sclerotized venom channel embedded in the cuticle. Additionally, as the tip of the Berkovich indenter is a few µm in size, indenting the thin cuticle of *C. hortensis* (6.37 to 12.25 µm) is at the limit of the reliability of the method [75]. Centipedes prey on a wide food spectrum, depending on their size, life cycle stage, habitat structure and also prey-to-body-size ratio [35, 36]. It seems unlikely that elemental enrichment of the cuticle of the tarsungulum depends on prey choice. Our results, however, indicate that the variation of elemental enrichment in the cuticle of the centipede forcipule might contribute to optimised mechanical stability in light of size differences across centipede lineages.

### The forcipular tarsungulum is a fusion of the tarsus and pretarsal claw

The centipede tarsungulum is defined as the ultimate (forth) element of the forcipule in Pleurostigmophora [76]. In Scutigeromorpha, Bonato et al. [76] define the fourth element as tarsus and the ultimate (fifth) articulated element as ungulum. Alternative definitions postulate that the forcipular tarsungulum, often referred to as tarsus or claw, is a fusion of two ancestral leg components—the tarsus and the pretarsal claw (ungulum or claw)—as described by Verhoeff [77], Snodgrass [78], and Dugon et al. [31]. Our results in combination with previously published data [31, 79], corroborate this later hypothesis. Chemoreceptive sensilla coeloconica found at the distal tarsungulum likely play a role in prey analysis during piercing and venom injection. These sensilla exhibit a short peg in an oval to circular pit. While three distinct types varying in shape, size, and distribution were identified in several species, they are consistently present across all five higher taxa, serving as a synapomorphic feature [79]. The proximal part of the tarsungulum (up to the suture) bears longer sensilla trichodea. The pretarsal claw of the locomotory leg in investigated taxa also features sensilla coeloconica, with longer trichoid sensilla exclusively present on the podomeres of the telopodite.

In *S. coleoptrata*, a continuous transverse suture (referred to as 'tarsal suture' by Dugon et al. [31]) is positioned at the tarsungulum's midpoint. While absent in all other examined centipedes, where the tarsungulum is fused [28], an incomplete suture is consistently present more proximally, externally visible along the closing-axis of the forcipule, as depicted in SEM and LM images by Dugon et al. [31], Maruzzo & Bonato [80], and Haug et al. [28] and in this contribution. Autofluorescence experiments conducted across multiple species (*L. forficatus, S. oraniensis, C. hortensis, S. maritima*) revealed a sclerotization gradient in the tarsungulum distal to this incomplete suture (primarily under 633 nm excitation). This transition region also aligns with the distribution of sensilla coeloconica. Elemental analyses further support these findings, with transition metal and/or halogen incorporation displaying a distinct border within the tarsungulum's cuticle (*C. hortensis, S. maritima, and H. subterraneus*), mirroring autofluorescence experiment outcomes.

Hence, the tarsungulum's evolutionary transformation in centipedes starts from a bipartite condition, separate in Scutigeromorpha and fused (though detectable) in Pleurostigmophora. A comparison to the serial homologous locomotory leg suggests a fusion proximal to the border marked by (1) a sclerotization gradient, (2) transition metal and/or halogen incorporation, and (3) the presence of sensilla coeloconica. Despite a lack of developmental data, we propose that this fusion likely aligns with the incomplete suture, potentially equivalent to the boundary between the locomotory leg's tarsus and pretarsal claw.

## Conclusion

Our study reveals parallels in the evolution of material composition and properties of the forcipules in centipedes. The forcipules transformed from an elongated leg-like appearance in Scutigeromorpha into a compact and rigid piercing structure in Epimorpha. Our data supports their serial homology to the locomotory leg. The forcipule’s tarsungulum is a fusion of tarsus and pretarsal claw supported by sclerotization gradients, elemental incorporation and the presence of specialised sensilla. Like other arthropods that use specific organs as tools, the centipede forcipule has to endure substantial stress during prey capture, skin penetration and venom injection. A sclerotization gradient is present in the pleurostigmophoran forcipule and the tarsungulum may be enriched with calcium, zinc or chlorine. Calcium or zinc incorporation, leads to comparable hardness, elasticity and the capability to endure breaking forces like in piercing structures of chelicerates and insects, but the locality of elemental incorporation does not increase *H* and *E* in centipedes, suggesting that they followed their own pathways in the evolutionary transformation of piercing tools. Thus, the performance of the forcipule as a highly effective injecting needle crowns centipedes as top predators in terrestrial environments.

### Supplementary Information

Additional file 1. Results from EDX measurements in atomic % of all examined species. **Sheet 1** EDX results for the locomotory leg 10 and the forcipule of *Scutigera coleoptrata*, *Lithobius forficatus*, *Craterostigmus tasmanianus*, *Scolopendra oraniensis*, *Cryptops hortensis*, *Strigamia maritima*, and *Haplophilus subterraneus*. **Sheet 2** Results from EDX point measurements for *Lithobius forficatus*. **Sheet 3** Results from EDX point measurements for *Cryptops hortensis*.

Additional file 2. PDF document with EDX mapping of all analysed elements in the cross-section of the pretarsal claw of *Cryptops hortensis*. Frontal View. The results of the analysis in atomic % and weight %.

Additional file 3. Cross-section of the tarsungulum of *Strigamia maritima*. Frontal view. **A** EDX mapping of Na in the tarsungular cross-section. The arrow points to the venom channel. **B** EDX mapping of Cl in the tarsungular cross-section. **C** EDX mapping of Ca in the tarsungular cross-section. **D** EDX mapping of Zn in the tarsungular cross-section.

Additional file 4. EDX mapping of Cl in the cross-section of the tarsungulum *Haplophilus subterraneus*. Arrow points to the venom channel.

Additional file 5.Mean hardness (*H*) and Young’s modulus (*E*) of the measured localities of the forcipules of *Lithobius forficatus* and *Cryptops hortensis*. **Sheet 1** Results for sample 1 of *L. forficatus*. **Sheet 2** Results for sample 2 of L. forficatus. **Sheet 3** Results for sample 1 of *C. hortensis*. **Sheet 4** Results for sample 2 of *C. hortensis*.

Additional file 6. Table with all forces, cross-sectional areas and resulting stresses measured in the forcipules of *Lithobius forficatus* and *Cryptops hortensis*.

## Data Availability

Raw data for EDX (Additional file 1,2), nanoindentation (Additional file 5) and breaking stress (Additional file 6) are provided in the additional files. The remaining data are available from the corresponding author upon reasonable request.

## Abbreviations

CLSM: Confocal laser scanning microscopy
E: Young’s modulus
EDX: Energy-dispersive X-ray spectroscopy
H: Hardness
LM: Light microscopy
microCT: Micro computed tomography
SEM: Scanning electron microscopy
