# Supplementary material for: Material composition and mechanical properties of the venom-injecting forcipules in centipedes
Source: Front Zool. 2024 Aug 23;21:21. doi: 10.1186/s12983-024-00543-1 (PMC11342574; doi:10.1186/s12983-024-00543-1)
Supplement: Supplementary file 2 — Additional file 2. PDF document with EDX mapping of all analysed elements in the cross-section of the pretarsal claw of Cryptops hortensis. Frontal View. The results of the analysis in atomic % and weight %. [file 12983_2024_543_MOESM2_ESM.pdf]

Cryptops hortensis - cross-section of pretarsal claw

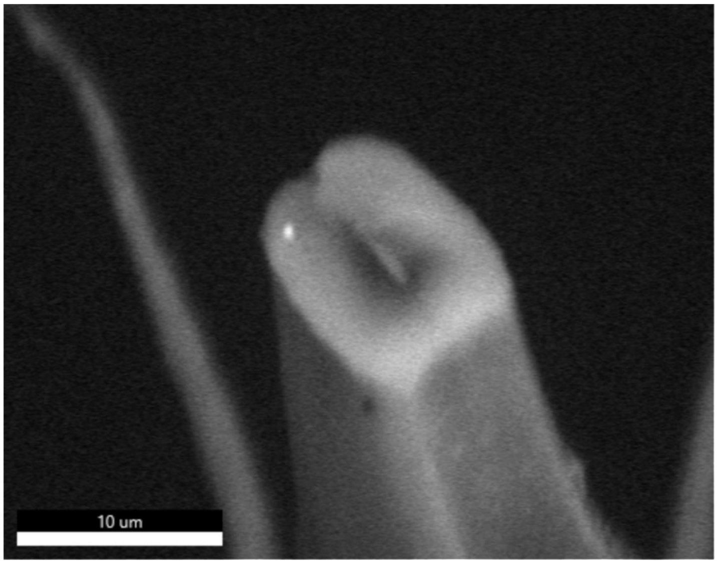

Image

Live Map 1

ElementOverlay

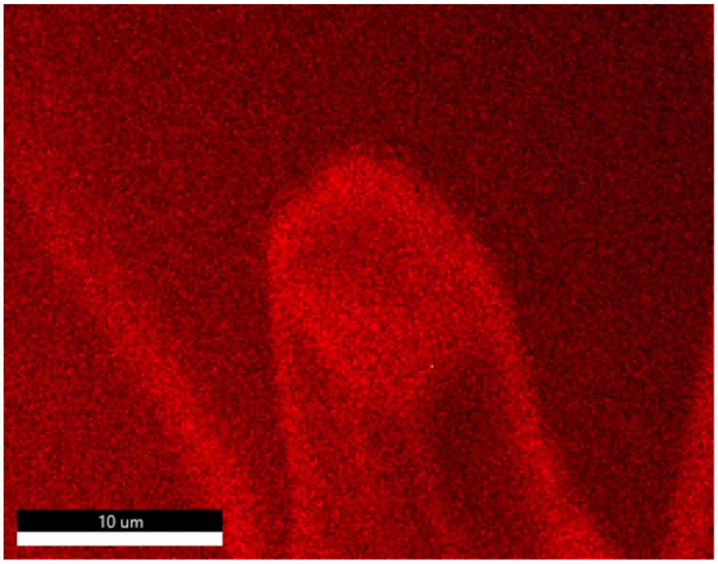

47% C K
2% N K
13% O K
7% NaK
2% MgK
2% AlK
2% SiK
3% P K
5% S K
2% ClK
2% K K
3% CaK
1% MnK
1% FeK
1% CoK
1% NiK
1% CuK
3% ZnK
1% AuL

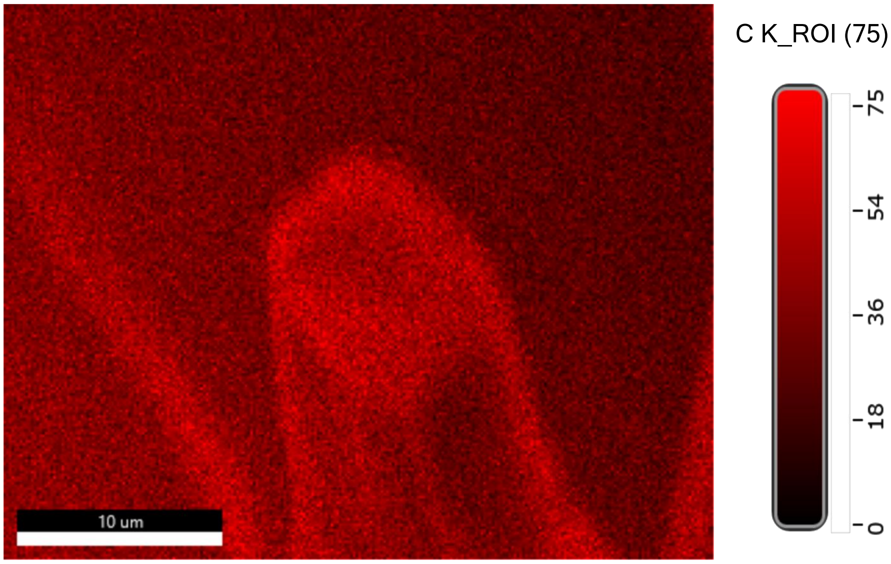

C K_ROI (75)

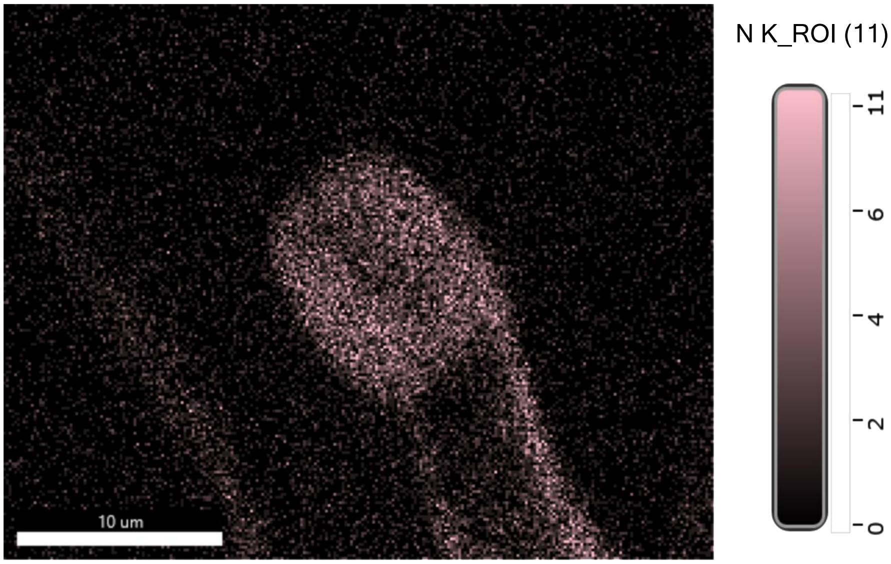

N K_ROI (11)

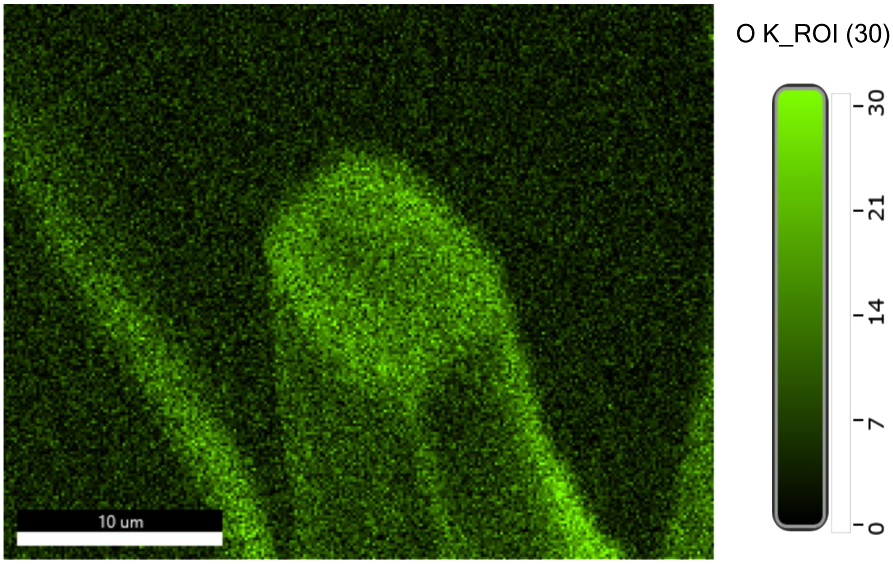

O K_ROI (30)

NaK_ROI (28)

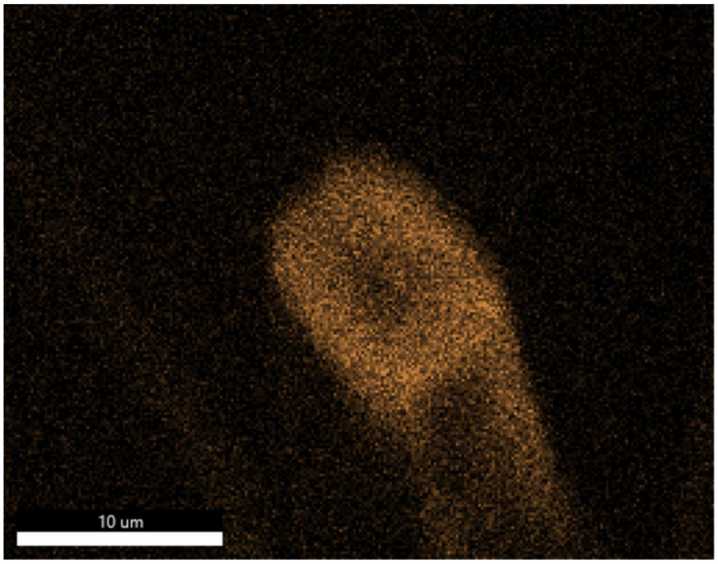

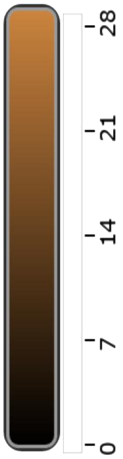

MgK_ROI (10)

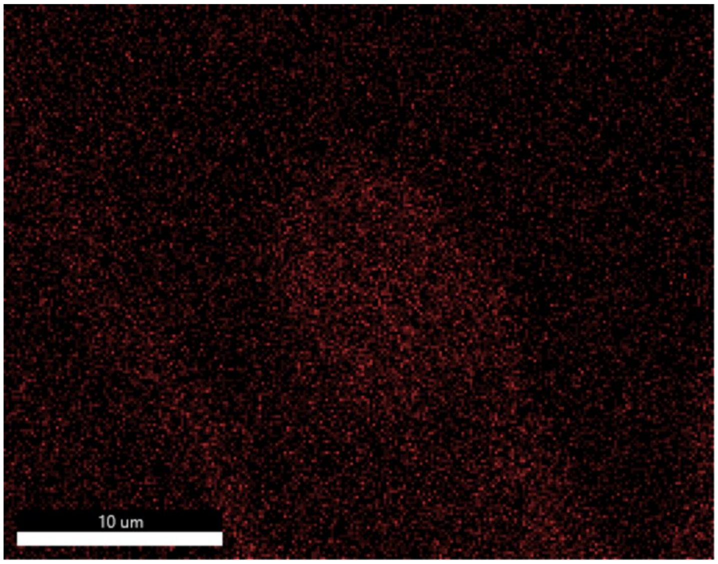

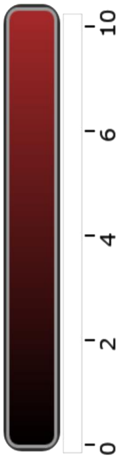

AlK_ROI (9)

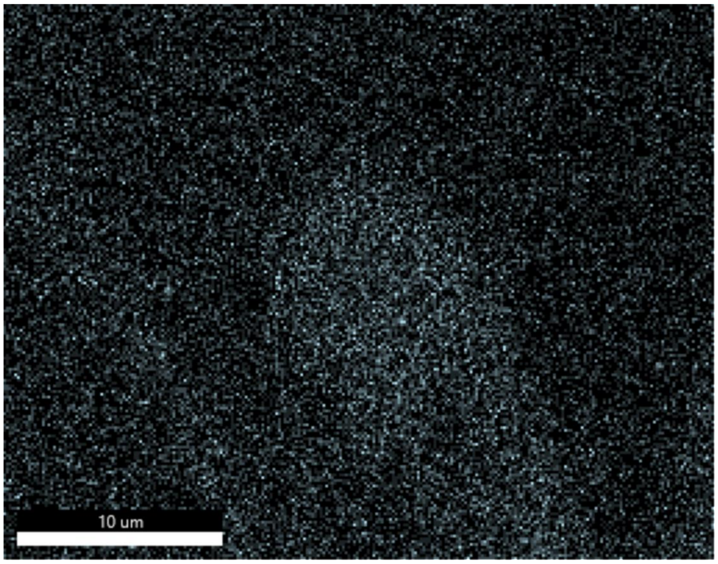

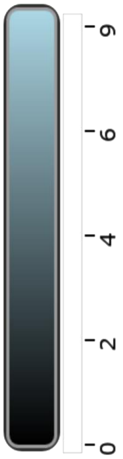

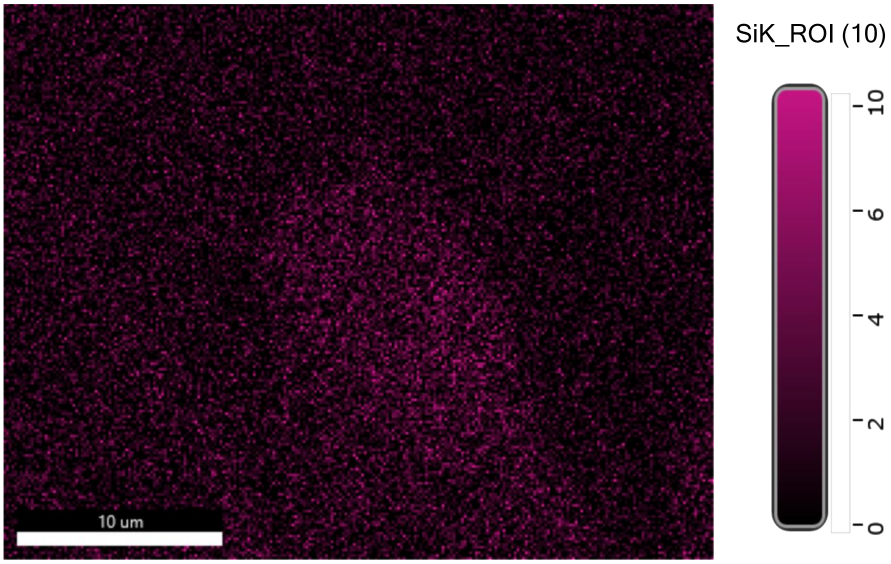
SiK_ROI (10)
10 um
10
6
4
2
0

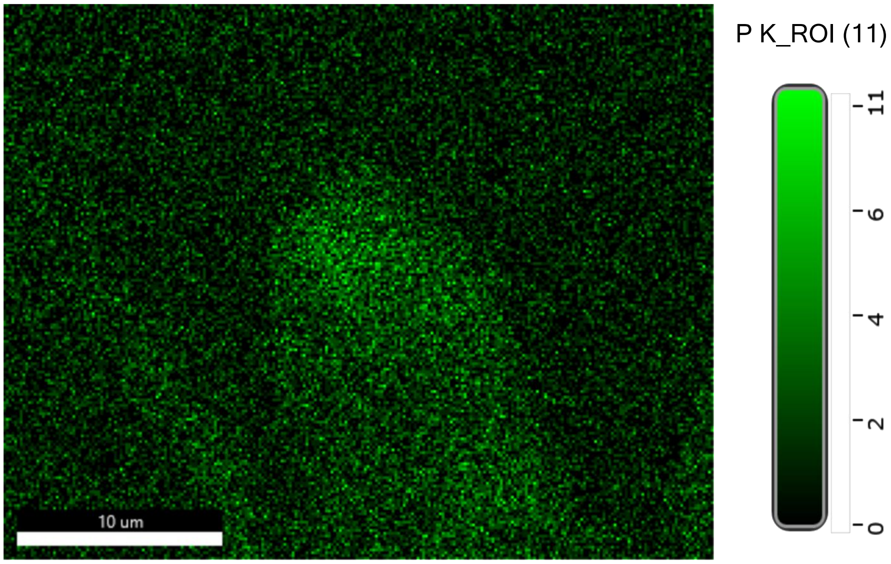
P K_ROI (11)
10 um
11
6
4
2
0

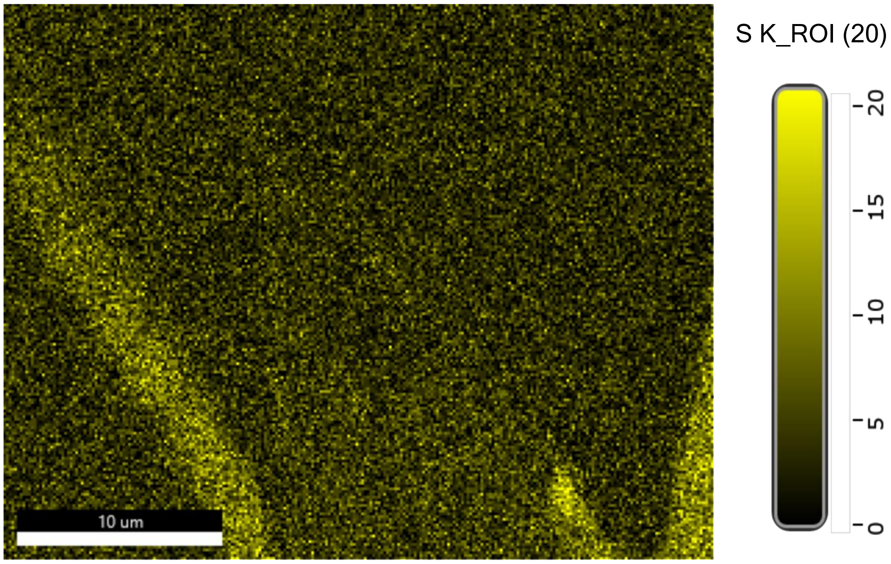
S K_ROI (20)
10 um
20
15
10
5
0

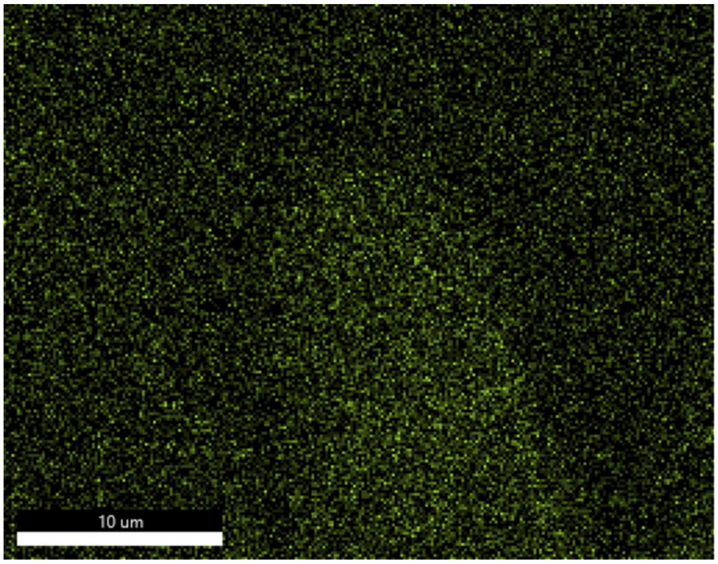

ClK_ROI (11)

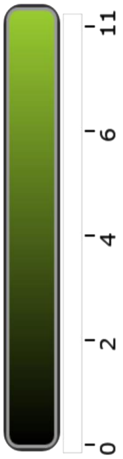

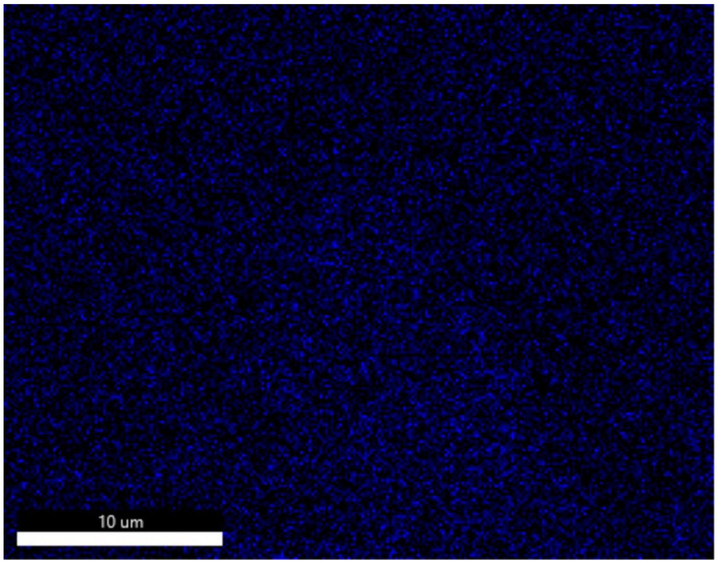

K K_ROI (10)

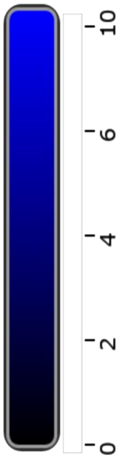

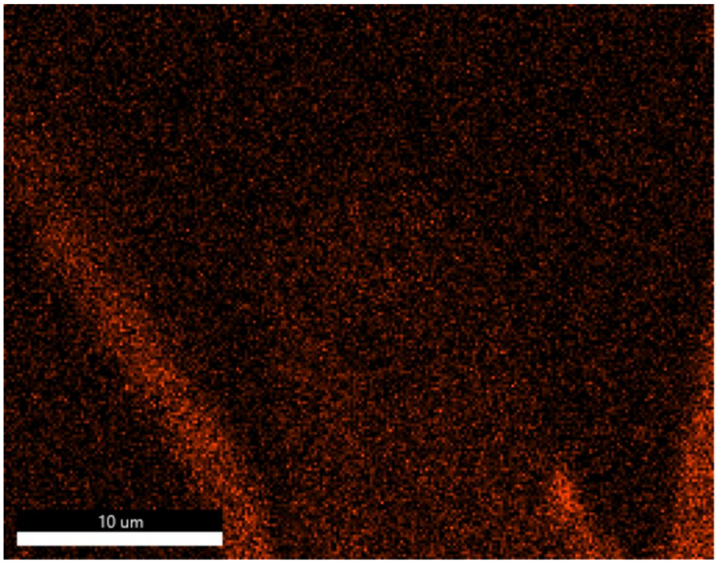

CaK_ROI (14)

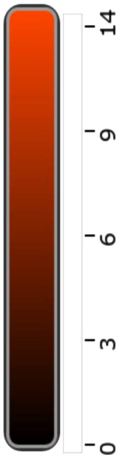

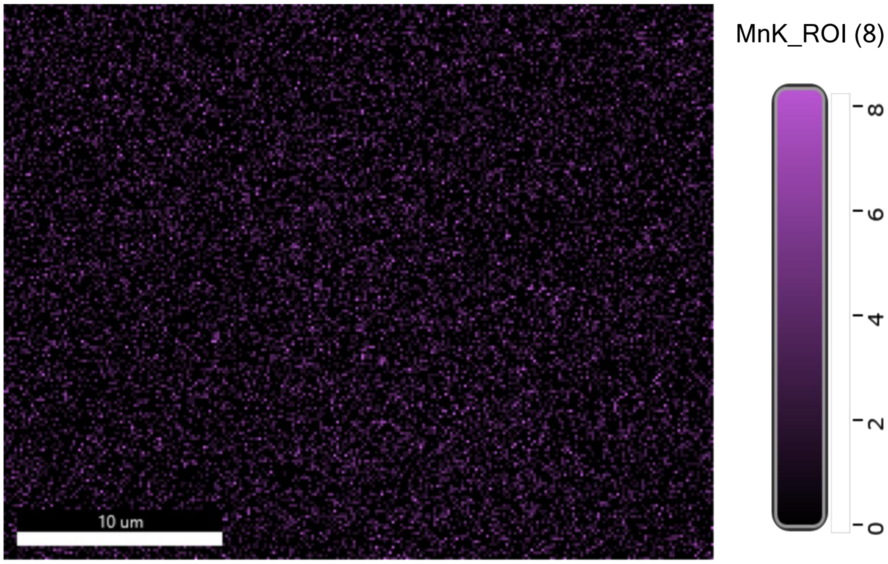
MnK_ROI (8)
10 um
8
6
4
2
0

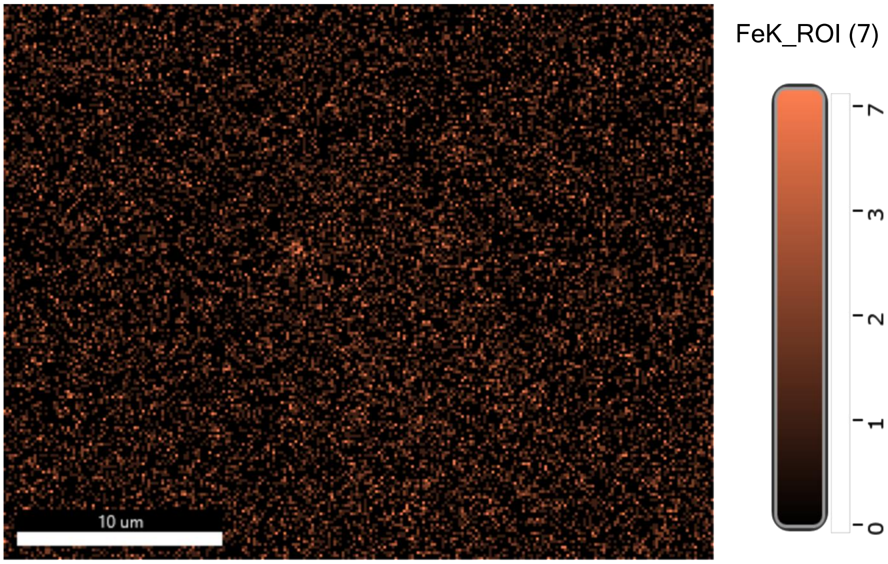
FeK_ROI (7)
10 um
7
3
2
1
0

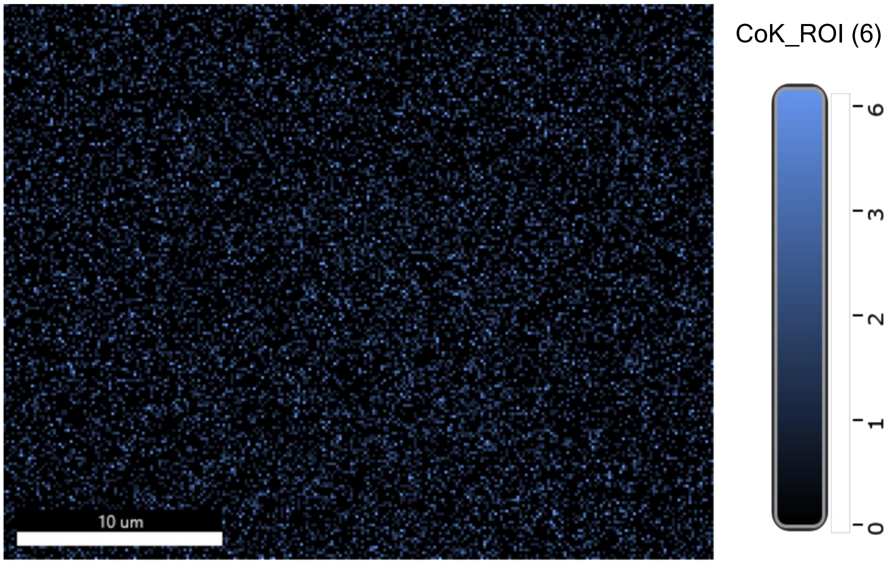
CoK_ROI (6)
10 um
6
3
2
1
0

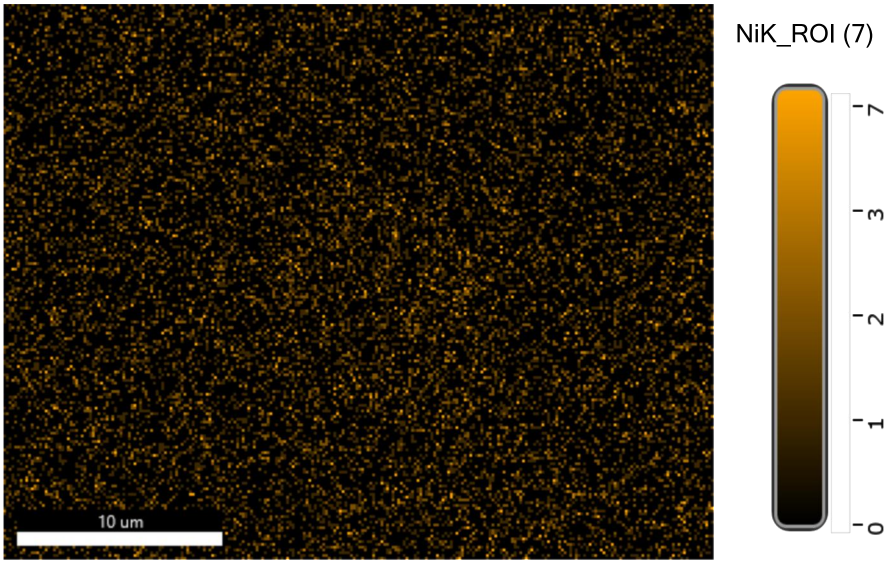
10 um
NiK_ROI (7)
7
3
2
1
0

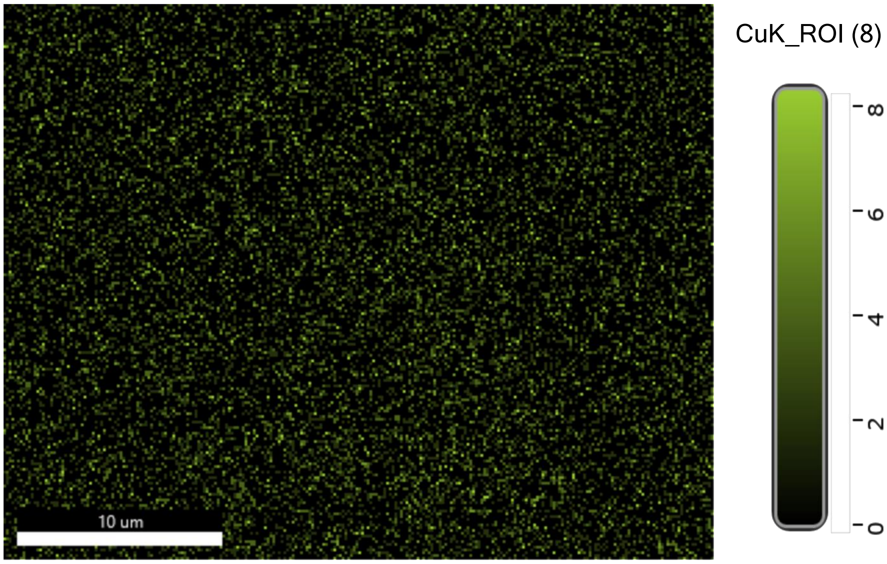
10 um
CuK_ROI (8)
8
6
4
2
0

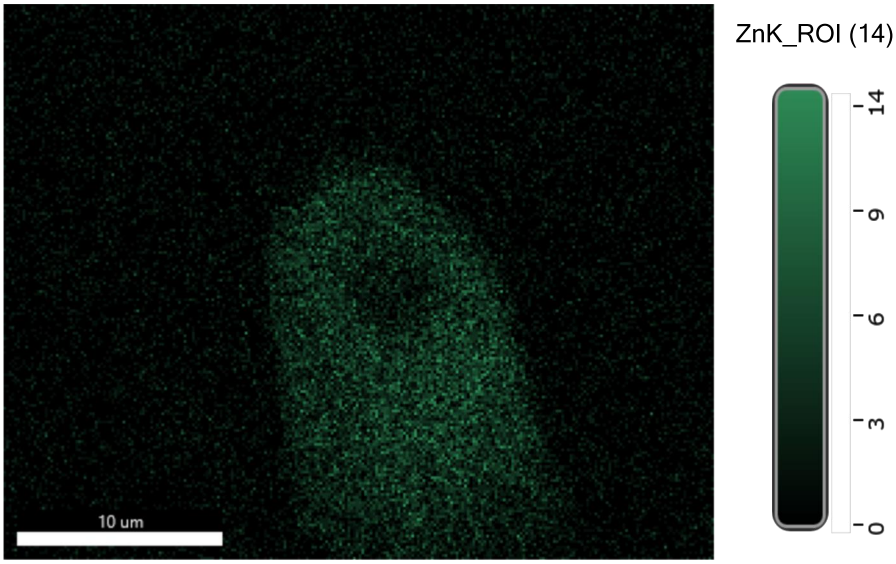
10 um
ZnK_ROI (14)
14
9
6
3
0

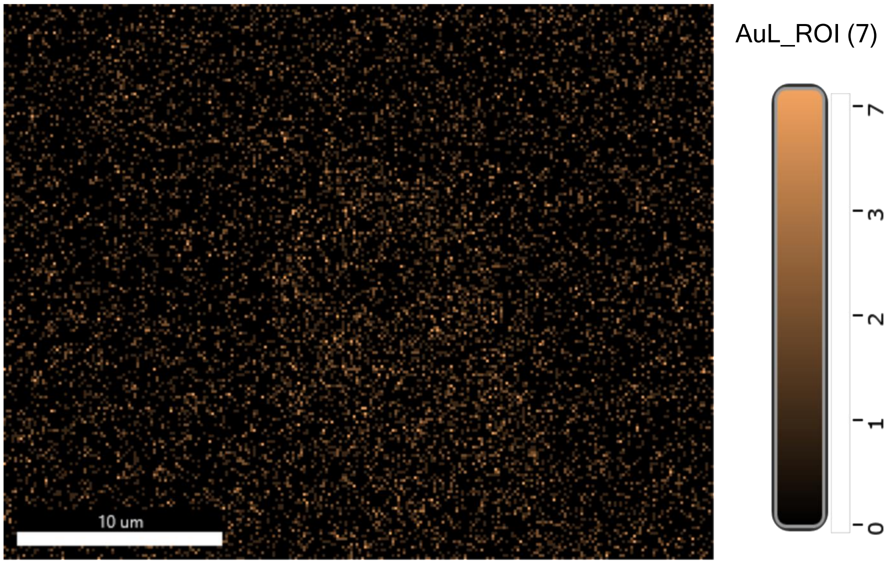
10 um
AuL_ROI (7)
7
3
2
1
0

EDAX APEX

kV: 20 Mag: 3700 Takeoff: 35 Live Time(s): 327.7 Amp Time(µs): 7.68 Resolution:(eV) 127.4

Sum Spectrum

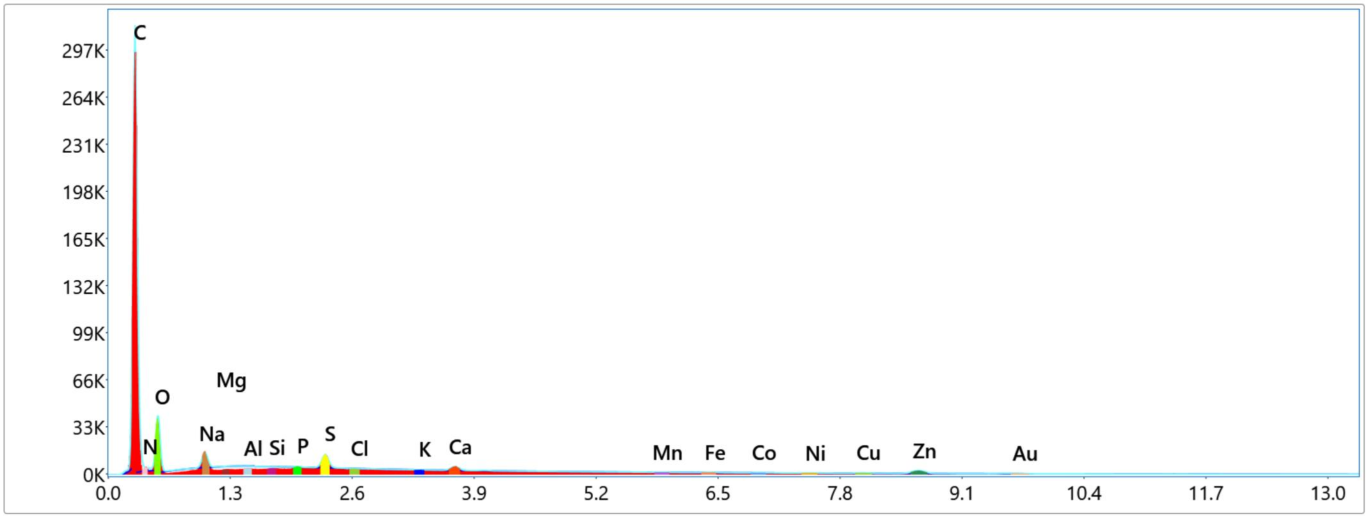

Det: Octane Plus

eZAF Quant Result - Analysis Uncertainty: 9.73 %

Element	Weight %	MDL	Atomic %	Net Int.	Error %	R	A	F
C K	75.98	0.18	82.38	3718.57	9.28	0.9354	0.1743	1.0000
N K	0.00	0.00	0.00	0.00	99.99	0.9398	0.0287	1.0000
O K	20.49	0.09	16.67	643.71	11.03	0.9434	0.0646	1.0000
Na K	0.38	0.03	0.21	74.50	9.75	0.9523	0.3071	1.0016
Mg K	0.00	0.00	0.00	0.00	99.99	0.9550	0.4506	1.0025
Al K	0.00	0.00	0.00	0.00	99.99	0.9575	0.5960	1.0041
Si K	0.00	0.00	0.00	0.00	99.99	0.9598	0.7129	1.0062
P K	0.02	0.01	0.01	8.20	25.37	0.9621	0.8006	1.0096
S K	0.55	0.01	0.22	255.01	3.23	0.9642	0.8587	1.0115
Cl K	0.00	0.00	0.00	0.00	99.99	0.9661	0.8956	1.0169
K K	0.00	0.00	0.00	0.58	80.64	0.9699	0.9463	1.0325
Ca K	0.31	0.02	0.10	92.68	4.44	0.9717	0.9612	1.0388
Mn K	0.05	0.03	0.01	8.37	29.83	0.9797	0.9909	1.1576
Fe K	0.08	0.03	0.02	12.34	22.74	0.9812	0.9932	1.1934
Co K	0.05	0.03	0.01	6.27	37.48	0.9827	0.9948	1.2448

Element	Weight %	MDL	Atomic %	Net Int.	Error %	R	A	F
Ni K	0.05	0.04	0.01	6.03	35.68	0.9841	0.9952	1.2892
Cu K	0.17	0.04	0.03	16.58	16.19	0.9856	0.9974	1.2752
Zn K	1.38	0.05	0.28	108.45	4.78	0.9870	0.9976	1.2471
Au L	0.49	0.21	0.03	8.85	37.28	0.9926	1.0004	1.0790
