# Supplementary figures and images for: Material composition and mechanical properties of the venom-injecting forcipules in centipedes
Source: Front Zool. 2024 Aug 23;21:21. doi: 10.1186/s12983-024-00543-1 (PMC11342574; doi:10.1186/s12983-024-00543-1)

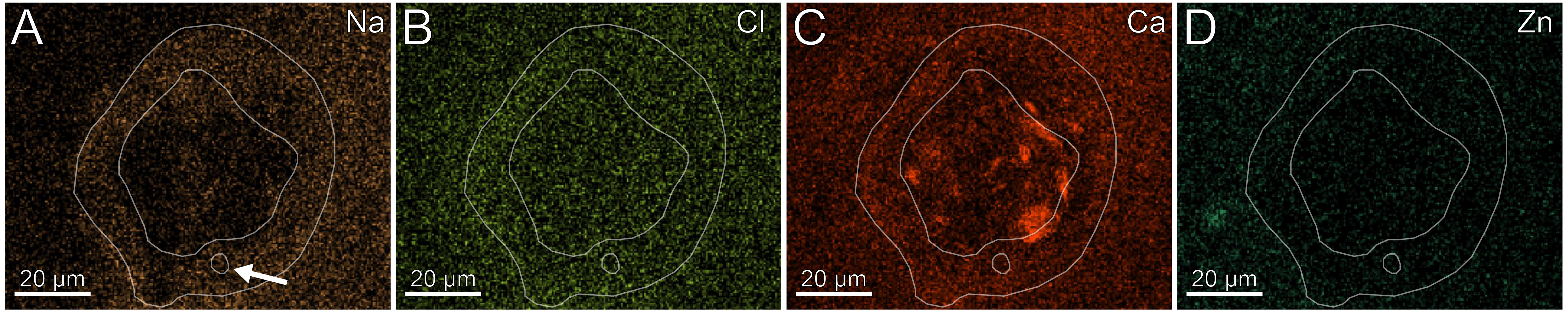

Supplement: Supplementary file 3 — Additional file 3. Cross-section of the tarsungulum of Strigamia maritima. Frontal view. A EDX mapping of Na in the tarsungular cross-section. The arrow points to the venom channel. B EDX mapping of Cl in the tarsungular cross-section. C EDX mapping of Ca in the tarsungular cross-section. D EDX mapping of Zn in the tarsungular cross-section. [file 12983_2024_543_MOESM3_ESM.tif]

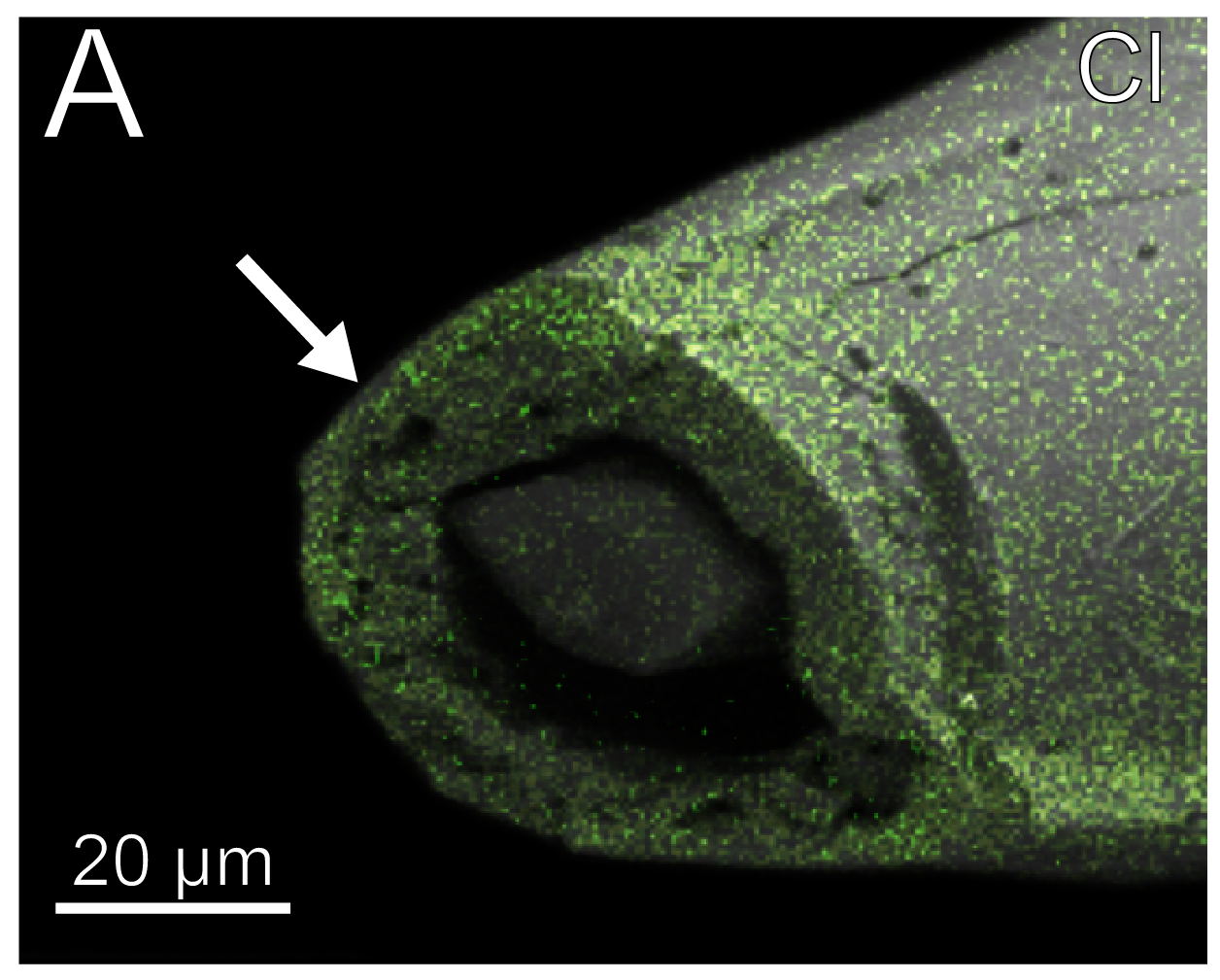

Supplement: Supplementary file 4 — Additional file 4. EDX mapping of Cl in the cross-section of the tarsungulum Haplophilus subterraneus. Arrow points to the venom channel. [file 12983_2024_543_MOESM4_ESM.tif]
